# Lgi3-4 proteins modulate the K_V_1.5 channelosome and are potential therapeutic targets for atrial fibrillation

**DOI:** 10.1093/cvr/cvag049

**Published:** 2026-03-19

**Authors:** Paula G Socuéllamos, Álvaro Macías, Ángela de Benito-Bueno, Francisco M Cruz, María Redondo-Moya, María José Coronado, Elvira Ramil, Silvia Rosado, Elsa Carolina Rios-Rosado, María Valencia-Avezuela, Laura Andrés-Delgado, José Antonio Blázquez, Alberto Forteza-Gil, Marta Gutiérrez-Rodríguez, José Jalife, Carmen Valenzuela

**Affiliations:** Instituto de Investigaciones Biomédicas Sols-Morreale CSIC-UAM, Arturo Duperier, 4, Madrid 28029, Spain; Centro Nacional de Investigaciones Cardiovasculares (CNIC), Melchor Fernández Almagro, 3, Madrid 28029, Spain; Centro Nacional de Investigaciones Cardiovasculares (CNIC), Melchor Fernández Almagro, 3, Madrid 28029, Spain; Instituto de Investigaciones Biomédicas Sols-Morreale CSIC-UAM, Arturo Duperier, 4, Madrid 28029, Spain; Centro Nacional de Investigaciones Cardiovasculares (CNIC), Melchor Fernández Almagro, 3, Madrid 28029, Spain; Instituto de Investigaciones Biomédicas Sols-Morreale CSIC-UAM, Arturo Duperier, 4, Madrid 28029, Spain; Institute of Health Research Puerta de Hierro-Segovia de Arana, IDIPHISA, Madrid, Spain; Institute of Health Research Puerta de Hierro-Segovia de Arana, IDIPHISA, Madrid, Spain; Institute of Health Research Puerta de Hierro-Segovia de Arana, IDIPHISA, Madrid, Spain; Departamento de Cirugía Cardiaca, Hospital Universitario Puerta de Hierro, Madrid, Spain; Instituto de Investigaciones Biomédicas Sols-Morreale CSIC-UAM, Arturo Duperier, 4, Madrid 28029, Spain; Departamento de Anatomía, Histología y Neurociencias, Facultad de Medicina, Universidad Autónoma de Madrid, Madrid, Spain; Departamento de Cirugía Cardiaca, Hospital Universitario La Paz, Madrid, Spain; Área Quirúrgica, Complejo Asistencial Universitario de Salamanca, Salamanca, Spain; Departamento de Cirugía Cardiaca, Hospital Universitario Puerta de Hierro, Madrid, Spain; Departamento de Biomiméticos, Instituto de Química Médica (IQM-CSIC), Madrid, Spain; Centro Nacional de Investigaciones Cardiovasculares (CNIC), Melchor Fernández Almagro, 3, Madrid 28029, Spain; Departments of Internal Medicine and Molecular and Integrative Physiology, University of Michigan, Ann Arbor, MI, USA; Centro de Investigación Biomédica en Red de Enfermedades Cardiovasculares (CIBERCV), Madrid, Spain; Instituto de Investigaciones Biomédicas Sols-Morreale CSIC-UAM, Arturo Duperier, 4, Madrid 28029, Spain; Centro de Investigación Biomédica en Red de Enfermedades Cardiovasculares (CIBERCV), Madrid, Spain

**Keywords:** Lgi proteins, K_V_1.5 channelosome, *I*_Kur_, Atrial fibrillation

## Abstract

**Aims:**

We investigated the role of Lgi3-4 proteins in cardiac electrophysiology, with a specific focus on *I*_Kur_, and their potential contribution to the pathophysiology of atrial fibrillation (AF).

**Methods and results:**

In human atrial tissue and heterologous cells, Lgi3 and Lgi4 interacted with K_V_1.5 channels. In a mouse model of AAV9-mediated cardiac-specific Lgi4 expression, sinoatrial and atrioventricular conduction were impaired, resulting in a prolonged QRS interval. Action potential repolarisation was delayed in atrial and ventricular cardiomyocytes from Lgi4 mice. In HEK293 cells, Lgi3-4 impaired K_V_1.5/K_V_β association, partially reversing the K_V_β-induced inactivation and reducing *I*_Kur_ amplitude. These results correlated with the reduced K_V_1.5 membrane expression and *I*_Kur_ density observed in Lgi4 cardiomyocytes and HEK293 cells. Notably, Lgi4 protein expression was lower in atrial tissue from patients with AF than in sinus rhythm patients. The reduced Lgi4 protein levels in AF were also associated with an altered colocalisation with K_V_1.5 channels, suggesting potential disruption in their functional interactions.

**Conclusion:**

We conclude that Lgi3-4 proteins are new components of the cardiac K_V_1.5 channelosome. They modulate *I*_Kur_ by interfering with the K_V_1.5-K_V_β interaction. Significantly, Lgi4 is dysregulated differently in paroxysmal vs. permanent AF. The results identify Lgi3-4 proteins as novel regulators of the K_V_1.5 channelosome, opening new pathways for investigating the role of *I*_Kur_ dysfunction in the mechanism of atrial fibrillation.

## Graphical Abstract

**Graphical Abstract:**
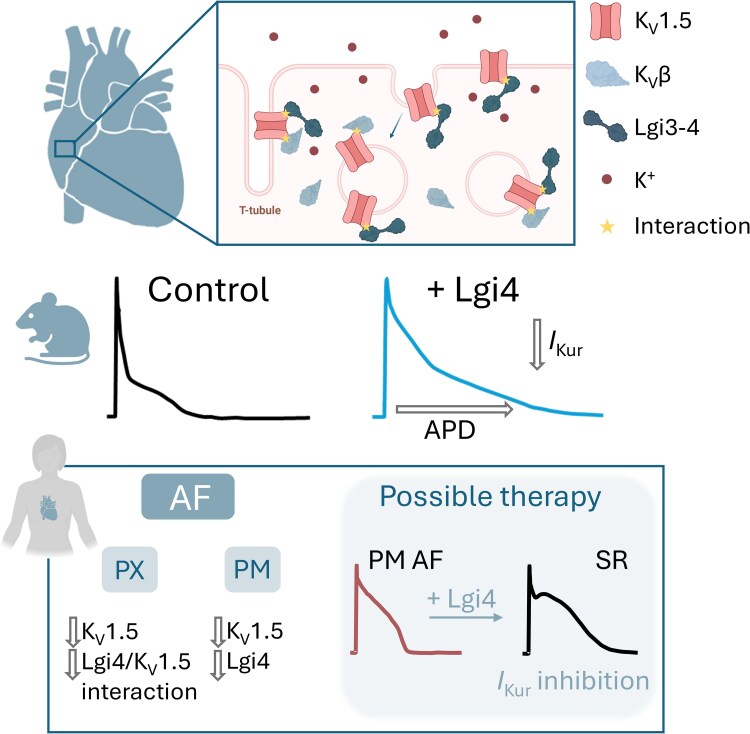

**Time of primary review: 47 days**

**See the editorial comment for this article ‘Tuning atrial repolarization by modulation of K_v_1.5 channelosome via Lgi3-4 proteins’, by U. Ravens *et al.*, https://doi.org/10.1093/cvr/cvag072.**

## 1. Introduction

Atrial fibrillation (AF) is the most common sustained arrhythmia and a leading cause of stroke.^1–3^ K_V_1.5 channels, coded by *KCNA5,* generate the ultrarapid outward potassium current (*I*_Kur_), an atrial-selective K^+^ current crucial for the initial phase of human atrial repolarisation.^3–5^  *I*_Kur_ is altered in AF patients and is a potential therapeutic target due to its atrial selectivity. Both gain- and loss-of-function mutations in *KCNA5* have been associated with AF susceptibility.^6,7^ Furthermore, K_V_1.5 expression is reduced in the atria of AF patients compared with those in sinus rhythm (SR).^5,8,9^ Anti-arrhythmic drugs are the first treatment option, but their effects are suboptimal, lacking sufficient efficacy and safety due to cardiac and extracardiac side effects.^3,10,11^ Even though several promising selective K_V_1.5 blockers have been developed in the last few years, no clinical trials have demonstrated a reduction of AF burden by K_V_1.5 inhibition. Therefore, whether the *I*_Kur_ block is effective in the pharmacological AF conversion to SR, or in reducing AF burden, remains unanswered. Thus, new therapeutic approaches are urgently needed and may be developed, for example, by deciphering the molecular basis of *I*_Kur_ and its regulation in AF.

In human cardiomyocytes, K_V_1.5 channels assemble and form macromolecular complexes (channelosomes) with several K_V_β subunits (K_V_β1.2, K_V_β1.3 and K_V_β2.1) that induce: 1) fast and partial N-type *I*_Kur_ inactivation (only in the case of K_V_β1.x); 2) a greater degree of C-type inactivation, 3) slower deactivation, and 4) a negative shift of the activation curve.^12–14^ Also, unlike heterologous systems,^15^ in mouse ventricular cardiomyocytes,^16^ K_V_β2.1 acts as a chaperone for KV1.5, increasing *I*_Kur_. However, the exact composition of these channelosomes remains undeciphered. Numerous interactors have been described recently, such as the membrane-associated guanylate kinase (MAGUK) family (i.e. SAP97),^17,18^ KChIP2^19^ or four-and-a-half Lim protein (FHL-1).^20^ Changes in the myocardial ion channels or their interactors may produce dramatic alterations in action potential waveforms, synchronisation, propagation and rhythmicity, thereby predisposing the heart to life-threatening arrhythmias.^3,21^

The Lgi protein family includes four members (Lgi1-4) encoded by four genes (*LGI1-4*). These proteins consist of a leucine-rich repeat (LRR) domain in the N-terminal and an epilepsy-associated or epitempin (EPTP) domain in the C-terminal.^22,23^ Recent studies demonstrated that Lgis have complementary roles in different parts of the nervous system and cannot be replaced by another member of the Lgi family.^24,25^ Lgi1-4 proteins play critical regulatory functions by interacting with different members of the ADAM (A Disintegrin And Metalloproteinase) family, ADAM23, ADAM22 and ADAM11.^26^ Lgi1 modulates synaptic transmission as the extracellular binding partner of ADAM22/23. Also, Lgi1 assembles with K_V_1.1, K_V_β1 and K_V_1.4 in neurons, selectively removing the N-type inactivation induced by the K_V_β1 subunit,^27^ and modulates the trafficking of K_V_1.1, K_V_1.2 and K_V_4.2 channels, tuning the action potential firing.^(28–30)^ Numerous mutations and deletions in the *LGI1* gene have been linked to autosomal dominant lateral temporal (lobe) epilepsy (ADLTE).^22,28,30–33^ Additionally, antibodies against Lgi1 have been associated with autoimmune encephalitis,^28,34–36^ which can produce bradyarrhythmia even though Lgi1 is not expressed in cardiomyocytes.^37^ Lgi3 modulates the K_V_1 channel complex localisation at the juxtaparanodes (JXP) in myelinated axons in the central nervous system (CNS). This effect is mediated by ADAM23.^24,38^ A cohort of patients carrying mutations in Lgi3 that display facial myokymia and developmental delay has been recently described.^38^ Lgi4 is a vital regulator of myelination in the peripheral nervous system via its interaction with ADAM22, and its dysfunction results in peripheral hypomyelination.^25,39–42^ Mutations in Lgi4 have been linked to arthrogryposis multiplex congenita, with a very early mortality rate.^42,43^ However, the role of these proteins in the heart remains undeciphered.

Here, we explored the role of Lgi3-4 proteins in cardiac electrophysiology, specifically looking at their impact on *I*_Kur_ and how they might contribute to AF development. Using three different biological systems and a multidisciplinary approach, we demonstrate for the first time that Lgi3-4 proteins are new components of the K_V_1.5 channelosome. We discovered that Lgi3-4 modulate *I*_Kur_ by interfering with the interaction between K_V_1.5 and the K_V_β subunits. Additionally, we found strong evidence that Lgi4 is dysregulated differently in paroxysmal vs. permanent AF, shedding new light on the mechanisms underlying this most common type of cardiac arrhythmia.

## 2. Methods

### 2.1 Sex as a biological variable

Human cardiac samples from patients in SR, paroxysmal (PX) and permanent (PM) AF were included from both sexes, but we did not include sex as a variable in the analysis because we could not assure an equal distribution due to the small number of samples in each group. Regarding mouse models, our study examined male mice because male animals exhibited less variability in phenotype.

### 2.2 Human samples

The Cardiac Surgery Service of the Health Research Institute of La Paz University Hospital (IdiPAZ) and Puerta de Hierro University Hospital provided human right atrial appendages. Ventricular tissue was kindly provided by the Departamento de Anatomía, Histología y Neurociencias of the Universidad Autónoma de Madrid (UAM).

### 2.3 Mice

C57BL/6J mice (5–6-weeks-old) were obtained from Charles River Laboratories. Mice were reared and housed following CNIC institutional guidelines and regulations. The mice had free access to food and water.

### 2.4 Adeno-associated virus vector production, purification, and mouse model generation

AAV vectors were generated encoding Lgi4 (NM_139284.3), followed by tdTomato reporter, or only tdTomato for the Control, under the control of the cardiomyocyte-specific cTnT (cardiac troponin T) proximal promoter. Vectors were packaged into AAV serotype 9 and produced by the triple-transfection method, using HEK293T cells as described previously.^44,45^ Mice were anaesthetized with ketamine (60 mg/kg) and xylazine (20 mg/kg) with a single administration via the intraperitoneal route. Thereafter, 3.5 × 10^10^ virus particles were inoculated through the femoral vein in a final volume of 50 μL as previously described.^46^ All experiments were performed 8 to 10 weeks after infection.

### 2.5 Cardiomyocyte isolation

Mouse atrial and ventricular cardiomyocytes (CMs) were isolated as previously described.^47^ Mice were euthanized in a CO_2_ chamber.

### 2.6 Cell culture

All cell lines were obtained from the American Type Culture Collection (Rockville, MD, USA). They were grown in their corresponding supplemented medium at 37°C in a 5% CO_2_ humidified atmosphere.

### 2.7 Patch clamp

The whole-cell patch-clamp technique, internal and external solutions, ion currents, action potential acquisition, and data analysis were similar to those previously described for cell lines^14,48^ and mouse cardiomyocytes.^47,49^

### 2.8 Protein extraction, immunoprecipitation and immunoblotting

HEK293 cells were homogenized with Lysis Buffer 1. Human atria tissue was mechanically homogenized with a Polytron (ULTRA-TURRAX^®^ T10 Basic Disperser, IKA® Works) with Lysis Buffer 2. Protein content was determined by using a BCA Pierce Kit. We used Protein A or G Sepharose^®^ beads incubated with the corresponding antibodies for coimmunoprecipitation assays. Protein extracts were separated by SDS-PAGE (8% acrylamide/bisacrylamide) gels, transferred to 0.45 μm PVDF membranes and incubated with different antibodies (See Supplementary material).

### 2.9 Immunofluorescence

Different immunofluorescence protocols were used in COS-7 cells,^14^ cardiomyocytes^47^ and human atria slices, as detailed in the Supplementary material.

### 2.10 Surface ECG recordings

Mice were anaesthetized using isoflurane inhalation (0.8%–1.0% volume in oxygen) and maintained at 37°C on a heating plate. Four-lead surface ECGs were recorded for 5 min using subcutaneous limb electrodes connected to an MP36R amplifier (BIOPAC Systems). Data acquisition and analysis were performed using the AcqKnowledge software.

### 2.11 In vivo intracardiac recording and stimulation

Mice were anesthetized with a single administration of ketamine (60 mg/kg) and xylazine (20 mg/kg) via the intraperitoneal route. An octopolar catheter (Science) was inserted through the jugular vein and advanced into the right atrium and right ventricle as previously described.^50^ Atrial and ventricular arrhythmia inducibility was assessed by applying consecutive S1 and S2 pulse trains at 10 and 25 Hz, respectively.

### 2.12 Statistical analyses

Data are expressed as mean ± SEM of n experiments, where N represents the number of patients or animals, and n represents the number of individual cells, IF images, or cell lysates. Comparisons were performed between different experimental groups by an unpaired two-tailed Student’s *t*-test or using multiple comparison Mann–Whitney’s test. When more than two experimental groups were compared, one-way or two-way ANOVA with Tukey’s or Šídák’s multiple comparison test, respectively, were used. Contingency analysis was performed with a chi-square test. Differences were considered significant when *P* < 0.05.

### 2.13 Study approval and ethics statement

The investigation conforms to the World Medical Association Declaration of Helsinki principles of medical research involving human subjects. All human samples were obtained with the appropriate informed consent, and their use was approved by the Ethics Committee of the La Paz University Hospital (PI-2550). Approval was also obtained from the Ethics Committee of the Puerta de Hierro University Hospital (PI-158-22), the Ethics Committee in Human and Animal Experimentation CEEHA and the Biosecurity Committee of the CSIC (PI-2550 and 046/2023). All procedures were done under the 1527/2010 (15 November 2010) and the 1716/2011 (18 November 2011) Royal Decrees, as well as the 9/2014 (4 July 2014) Royal Decree-Law. All animal procedures conformed to Directive 2010/63/EU guidelines of the European Parliament on the protection of animals used for scientific purposes and to Recommendation 2007/526/EC, enforced in Spanish law under Real Decreto 53/2013. Animal protocols followed the Spanish National Center for Cardiovascular Research (CNIC) Institutional Ethics Committee recommendations and were approved by the Animal Experimentation Committee (Scientific Procedures) of Comunidad de Madrid (PROEX 111.4/20 and PROEX 226.5/23).

### 2.14 Data availability

The data that support the findings of this study are available from the corresponding author upon reasonable request.

## 3. Results

### 3.1 Lgi protein expression and co-localisation with K_V_1.5 channels in the human heart

**Figure 1:**
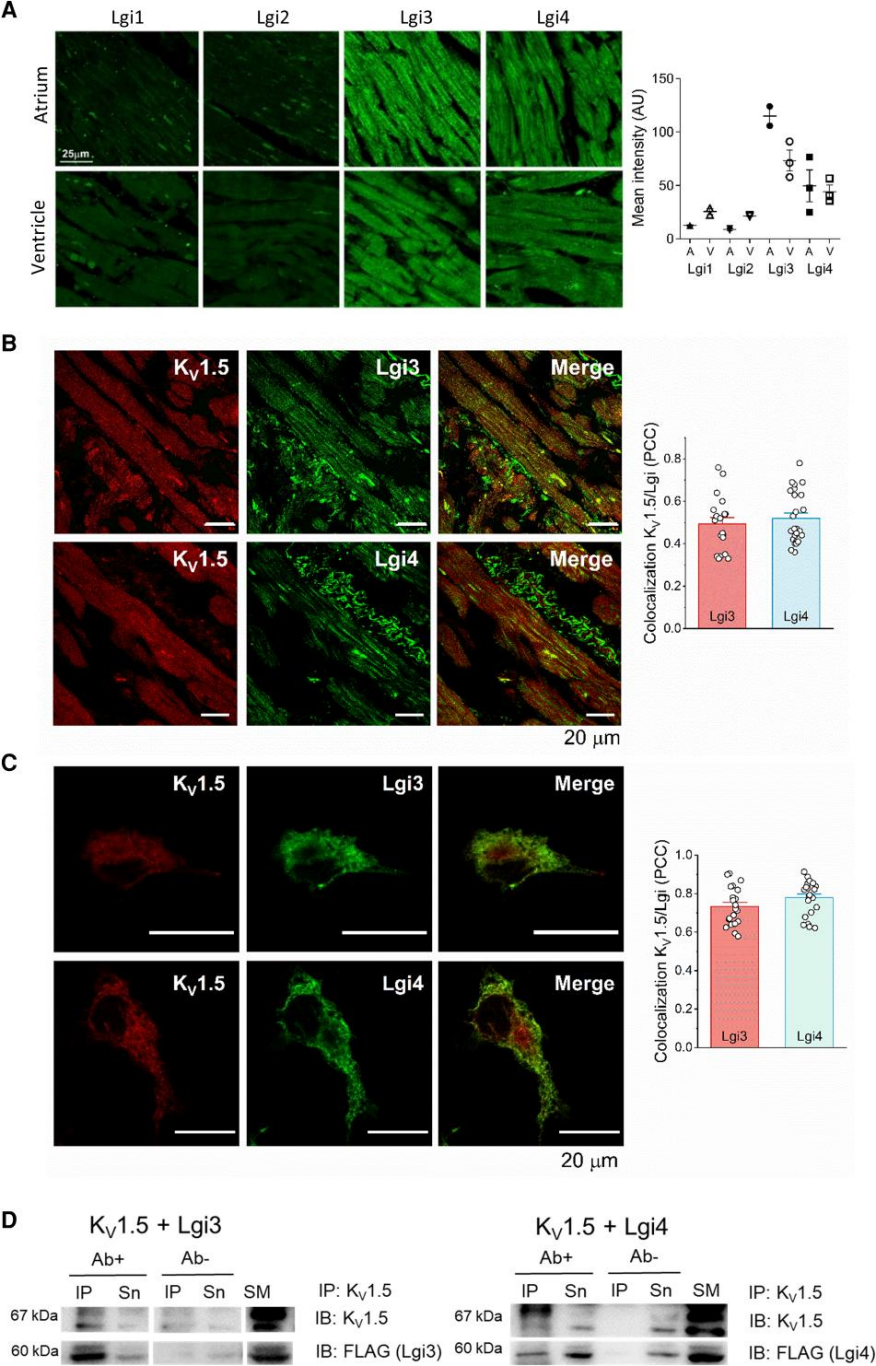
Lgi3-4 are expressed in human myocardium and interact with K_V_1.5. *A*) Confocal images of the immunodetection of Lgi1-4 in human atrium and ventricle slices obtained from healthy patients. Right graph: quantification of the Lgis expression in atrium (A) and ventricle (V). The intensity mean was measured as the integrated density. Values represent mean ± SEM of N = 2–3 donors, n = 9. Scale bar: 25 µm. *B*) Representative confocal images of the immunodetection of K_V_1.5 along with Lgi3 (upper panels) and Lgi4 (lower panels) in human atrium from patients in SR (N = 4–5, n = 20–25). Pearson’s correlation coefficient (PCC) between either Lgi3-4 and K_V_1.5 is shown in the right graph. Values represent mean ± SEM. Scale bar: 20 µm. *C*) Representative confocal images of COS7 cells cotransfected with K_V_1.5 and Lgi3 (upper panels) or Lgi4 (lower panels), with the PCC shown in the right bar graph. Data represent mean ± SEM of n = 22–23. Scale bar: 20 µm. *D*) Coimmunoprecipitation of K_V_1.5 with Lgi3 (left) or Lgi4 (right) coexpressed in HEK293 cells, using the anti-K_V_1.5 antibody (IP: K_V_1.5) and immunoblotting (IB) K_V_1.5 and FLAG (Lgi3-4) (n = 3). SM, Starting Material; IP, Immunoprecipitated; Sn, Supernatant; IB, Immunoblotting. In all experimental conditions, at least three independent experiments were performed. Note that the lanes were run on the same gel but were noncontiguous.

To determine the expression pattern of Lgi proteins in human myocardium, we performed immunofluorescence analysis in samples from the right atrium and right ventricle. Only Lgi3 and Lgi4 were expressed in human atrial and ventricular cardiomyocytes; Lgi1 and Lgi2 were nearly absent (*Figure 1A*). Positive controls of Lgi1-4 antibodies in brain slices demonstrated that the absence of Lgi1-2 in cardiac samples was not due to a lack of antibody efficiency (see Supplementary material online, *Figure S1*). Double staining of Lgi3-4 and K_V_1.5 revealed the colocalisation of Lgi3 (upper panels) and Lgi4 (lower panels) with K_V_1.5 in the human atrium (*Figure 1B*) and COS-7 cells (*Figure 1C*) with Pearson’s correlation coefficients (PCC) of about 0.5 and 0.8, respectively. For this analysis, the cardiomyocytes were selected within a region of interest. Lgi3-4 were expressed mainly in the endoplasmic reticulum (ER) and plasma membrane (*Figure 1C*, Supplementary material online, *Figure S2*), as previously reported.^39^ Coimmunoprecipitation studies confirmed the interaction between Lgi3-4 and K_V_1.5 (*Figure 1D*).

### 3.2 Cardiac-specific Lgi4 mice have abnormal ECGs and cardiac conduction

**Figure 2:**
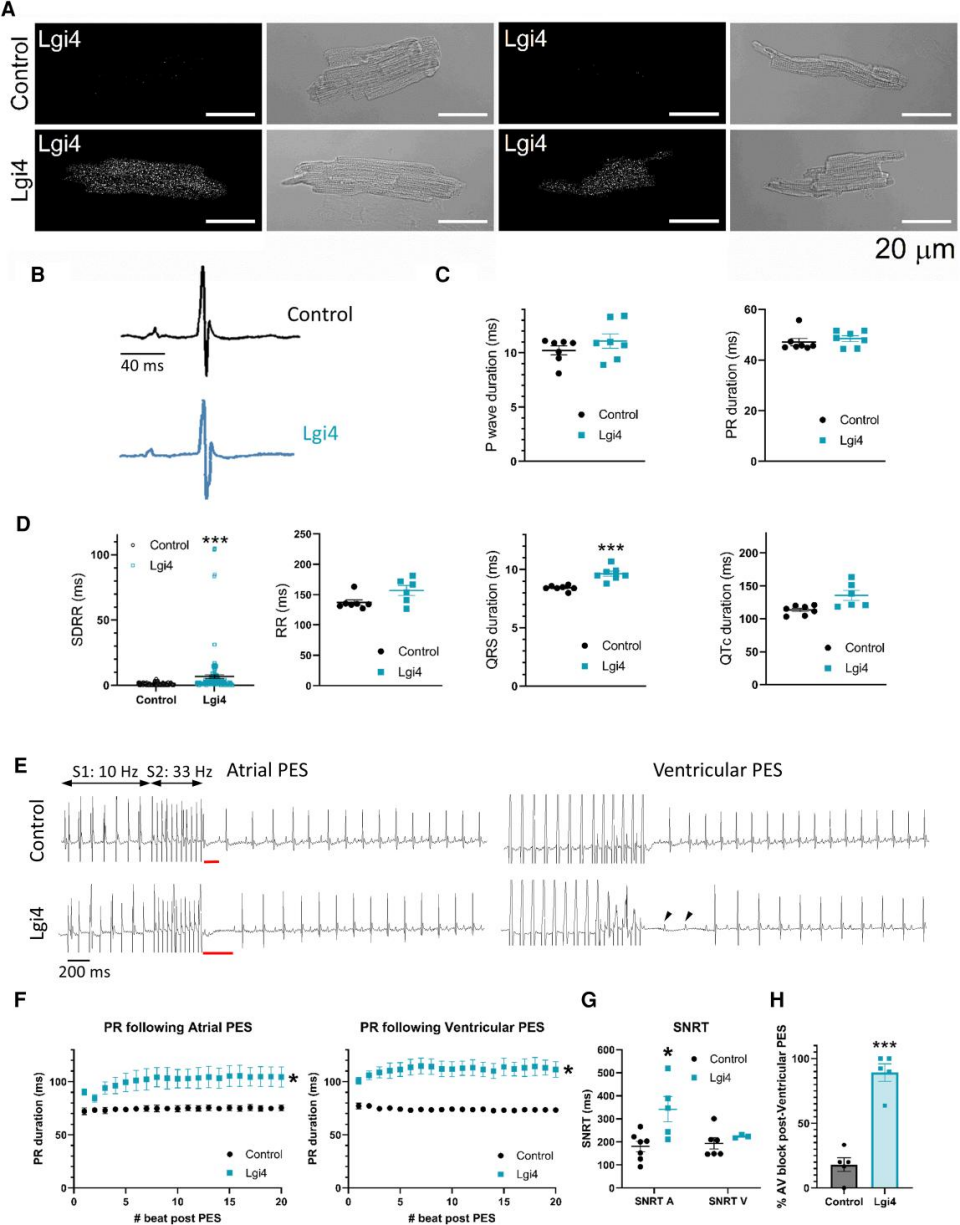
Lgi4 mice have abnormal ECGs and cardiac conduction. *A*) Representative examples of the immunodetection of Lgi4 in permeabilized mice ventricular CMs infected with Control (upper panels) or with Lgi4 expressing (lower panels) AAV9 (N = 3, n = 30). The corresponding bright field images are shown next to each immunostaining. Scale bar: 20 µm. *B*) Representative ECG traces of Control (black) and Lgi4 (blue) mice. *C*) P wave, RR, PR, QRS and QTc intervals in control (black) and Lgi4 (blue) mice. Note that the QRS is prolonged in the ECG from Lgi4 mice. Scale bar: 40 ms. *D*) Beat-to-beat variability is represented as the standard deviation of the RR (SDRR). Data are represented as mean ± SEM of N = 7 (Non-paired *t*-test ***P < 0.001). *E*) Original lead-II ECG recordings during and after atrial (A) and ventricular (V) PES in Control (above) and Lgi4 (below) mice. The Sinus node recovery time (SNRT) is shown in re,d and AV blocks are indicated with arrows. Scale bar: 200 ms. *F*) PR duration after atrial (left) and ventricular (right) stimulation in Control (black) and Lgi4 (blue) mice. *G*) SNRT after atrial (A) and ventricular (V) stimulation in Control (black) and Lgi4 (blue) mice. Each point represents the mean of approximately 10 PES in each mouse. *H*) Percentage of atrial-ventricular block following ventricular PES in Control (Black) and Lgi4 (blue) mice. Data are represented as mean ± SEM (N = 5–7). Non-paired *t*-test *P < 0.05, ***P < 0.001.

We investigated the in vivo contribution of Lgi proteins to cardiac electrical function. We first analysed the expression pattern of Lgi3 and Lgi4 in mouse atrial and ventricular tissues (see Supplementary material online, *Figure S3A*) and isolated ventricular CMs (see Supplementary material online, *Figure S3B*). Lgi3, but not Lgi4, was detected and widely distributed in both atria and ventricles. To study the modulation of *I*_Kur_ by Lgi4 in a physiological context, we used adeno-associated virus (AAV) technology^46^ to generate a mouse model with cardiac-specific expression of Lgi4 under the control of the cTnT promoter (see Supplemental Methods for details). We demonstrated Lgi4 expression in Lgi4-transduced CMs (*Figure 2A*). Cardiac Lgi3 expression was unaltered in the Lgi4-expressing mice (see Supplementary material online, *Figure S4*).

We first investigated the effects of cardiac-specific Lgi4 expression on surface-ECG lead II parameters (*Figure 2B*). Lgi4 expression did not modify the P wave, PR, and QTc intervals. However, the QRS interval was significantly longer in Lgi4 than in control mice (*Figure 2C*). In addition, RR variability was increased in the Lgi4 model, as shown by the increased RR standard deviation (SDRR) (*Figure 2D*), without changing the mean RR interval duration (*Figure 2C*).

To determine whether cardiac Lgi4 expression is arrhythmogenic in anesthetized mice, we used transvenous intracardiac programmed electrical stimulation (PES) in Lgi4 and control mice (*Figure 2E*). No atrial or ventricular arrhythmias were detected in either group. However, the PR interval was progressively prolonged after atrial and ventricular PES in Lgi4 compared with control mice (*Figure 2F*), which may indicate that atrioventricular (AV) node or Purkinje fiber conduction was altered. In addition, the sinus node recovery time (SNRT) after high-frequency PES stimulation of the atria was prolonged in Lgi4 mice compared to control mice (*Figure 2G*), revealing an alteration in sinoatrial conduction or prolongation of the atrial refractory period. Additionally, in contrast to the control, ventricular PES led to AV prolongation and block in Lgi4 mice (*Figure 2H*), again suggesting that Lgi4 affects AV or Purkinje fiber conduction. These results, together with the substantially increased RR variability, indicate that cardiac expression of Lgi4 is detrimental to the cardiac conduction system.

### 3.3 Lgi4 modulates action potential properties in mouse cardiomyocytes

**Figure 3:**
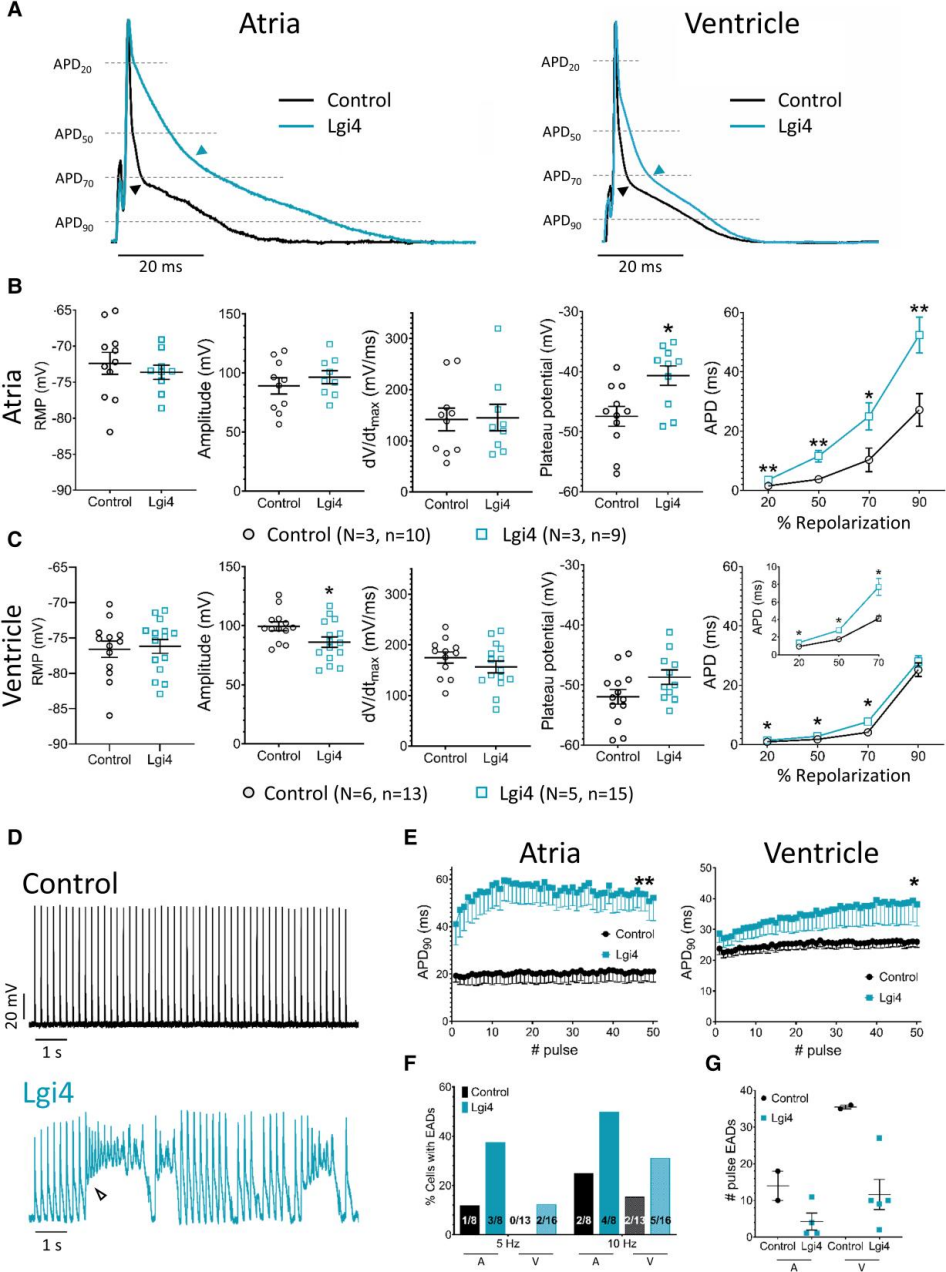
Lgi4 modulates early repolarisation and induces APD prolongation and EADs at high stimulation frequencies. *A*) Representative normalized recordings of APs registered at 1 Hz of stimulation in Control (black) and Lgi4 (blue) mice atrial (left) and ventricular (right) CMs. Black and blue arrows indicate the plateau potential in each condition. Scale bar: 20 ms. *B*) The resting membrane potential (RMP), maximal amplitude of the AP (Amplitude), maximum upstroke velocity (dV/dt_max_), plateau potential, and the APD measured at the 20, 50, 70 and 90% of repolarisation in atrial CMs stimulated at 1 Hz are represented. Note that APD_20_ to APD_90_ are far longer in CMs from Lgi4 mice than in Control ones. *C*) Quantification of the resting membrane potential (RMP), maximal amplitude of the AP (Amplitude), maximum upstroke velocity (dV/dt_max_), plateau potential, and the APD measured at the 20, 50, 70 and 90% of repolarisation in ventricular CMs stimulated at 1 Hz. APD_20_ to APD_70_ are longer in CMs from Lgi4 mice than in Control ones. *D*) Original recordings of APs generated when stimulated at 5 Hz in Control (black) and Lgi4 (blue) ventricular CMs. Note the EADs in Lgi4 CMs. Scale bar: 1 s in the *x-axis* and 20 mV in the *y-axis*. *E*) APD_90_ variability in Control (black) and Lgi4 (blue) when atrial (left) and ventricular (right) CMs are stimulated at 5 Hz. *F*) Percentage of cells exhibiting EADs when stimulated at 5 and 10 Hz, and *G*) Number of the pulse in which the first EADs appear at 10 Hz, respectively, in control (black) and Lgi4 (blue) atrial (A) and ventricular (V) CMs. Values are represented as mean ± SEM (N = 3–6, n = 9–15). (Two-tailed t-tests (*A–C*), mixed effects analysis and chi-square test (*E*, *F*). *P < 0.05).

We next studied the effects of Lgi4 on action potential (AP) depolarisation and repolarisation in CMs from the atria and the ventricles.^51^ As shown in *Figure 3*, Lgi4 expression did not modify the resting membrane potential (RMP), AP amplitude and maximal upstroke velocity (dV/dt_max_) in atrial (*Figure 3B*) and ventricular (*Figure 3C*) CMs. Conversely, the plateau potential, measured as the shifting point during repolarisation, was more depolarized in Lgi4-expressing CMs, especially in the atria (*Figure 3B*). Additionally, APD (measured at 20 to 90% repolarisation) was significantly more prolonged in Lgi4 than in control atrial CMs (*Figure 3B*). In contrast, it was only mildly prolonged in ventricular CMs (from 20 to 70% repolarisation), consistent with a slower early repolarisation. However, APD_90_, which accounts for the total duration of repolarisation, was not modified in ventricular CMs (*Figure 3C*). Changes in the AP waveform in Lgi4-transduced CMs can have different functional consequences.^52^ As illustrated by the representative recordings in *Figure 3D*, unlike control (top), during the application of a train of pulses at 5 Hz, Lgi4 CMs showed progressive APD prolongation, the effect being greater in atrial than ventricular CMs (*Figure 3E*). In addition, as shown by the lower panels of *Figure 3D* and *F*, during the 5-Hz train, some Lgi4 CMs exhibited excessive APD prolongation and early afterdepolarisations (EADs) that interfered with the stimulation. In *Figure 3F*, the percentage of control and Lgi4 CMs exhibiting excessive APD prolongation plus EADs increased substantially when stimulated at 10 Hz compared to 5 Hz. In *Figure 3G*, the first pulse with such features occurred earlier in Lgi4 than in control CMs. The APD was progressively more prolonged as stimulation frequencies increased from 1 to 10 Hz in atrial and ventricular CMs are shown in Supplementary material online, *Figure S5*

### 3.4 Lgi4 reduces *I*_Kur_ and K_V_1.5 membrane expression in ventricular CMs

**Figure 4:**
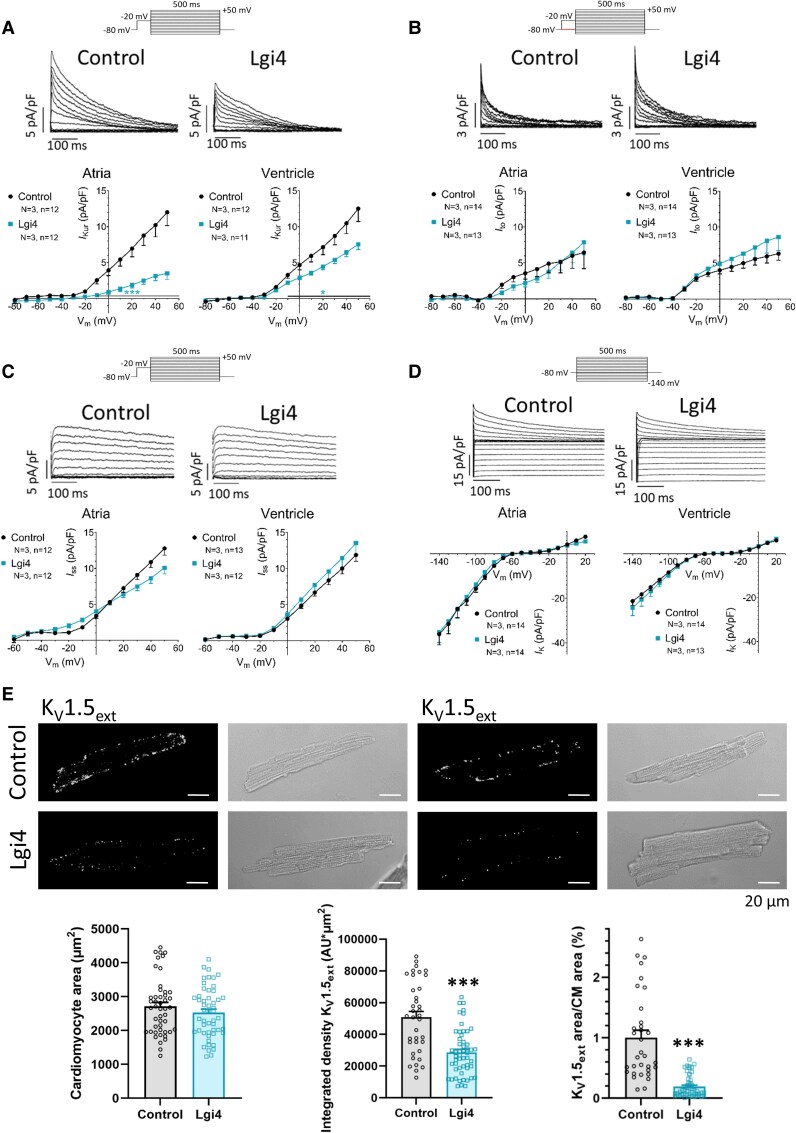
Lgi4 decreases *I*_Kur_ current amplitude and K_V_1.5 plasmalemmal expression in mice CMs. Original current traces of the outward potassium currents *I*_Kur_ (*A*), *I*_to_ (*B*) and *I*_ss_ (*C*) and the inward K^+^ current (*D*) recorded in Control (left) and Lgi4 (right) mice CMs, together with the current-amplitude (I-V) relationship for each current measured at the maximum peak amplitude in *I*_Kur_ (*A*), *I*_to_ (*B*), and *I*_ss_ (*C*) or at the end of the pulse in the inward K^+^ current (*D*) in mice atrial (left) and ventricular (right) CMs Control (black) and Lgi4 (blue). The voltage protocols used to record each current are represented above their corresponding current traces. Briefly, *I*_to_ was measured as the difference of the current obtained by subtracting records with an inactivating pre-pulse voltage-clamp protocol (black) from those without the inactivating pre-pulse (red). *I*_Kur_ is calculated by subtracting the currents recorded with the inactivating pre-pulse in the presence of 200 μM 4-aminopyridine (4-AP) from those recorded in its absence, and the remaining 4-AP-resistant current is *I*_ss_. Note that only *I*_Kur_ is reduced in Lgi4 CMs, and that such reduction is greater in atrial than in ventricular CMs. Data are represented as mean ± SEM (N = 3, n = 11–14). A non-paired *t*-test was performed comparing each condition with its corresponding control. *P < 0.05. Scale bar: 100 ms at *x* axis and the pA/pF indicated in each *y* axis. (*E*) Representative confocal images of the immunodetection of extracellular K_V_1.5 (plasmalemmal) in non-permeabilized mice ventricular CMs, Control and Lgi4. The corresponding bright field images are shown next to each immunostaining. Scale bar: 20 µm. Images clearly show that K_V_1.5 is less expressed in the plasmalemma in Lgi4 CMs than in Control ones. The quantification of the CMs area (left), the integrated density of K_V_1.5_ext_. Both analyses show that K_V_1.5 membrane expression is decreased in Lgi4 CMs. Data are represented as mean ± SEM (N = 4, n = 33–53). Non-paired *t*-test ***P < 0.001.

We hypothesized that the prolonged repolarisation in Lgi4 CMs is due to a decrease in *I*_Kur_. Thus, we measured the repolarising K^+^ currents in our AAV-mediated mouse models. Voltage-clamp experiments showed a significant *I*_Kur_ reduction in Lgi4 compared to control CMs (*Figure 4A*), *I*_Kur_ being more dramatically reduced in atrial than ventricular CMs (*Figure 4B*). *I*_to_, generated after the activation of K_V_4.3/K_V_4.2,^53^ was not modified in Lgi4 CMs. To study a possible differential Lgi4 modulation on each K_V_4 channel subtype, we transfected CHO-K1 cells with K_V_4.3/Lgi3-4 or K_V_4.2/Lgi3-4 (see Supplementary material online, *Figure S6A*–*D*). The experiments revealed that Lgi3-4 increased the current amplitude of K_V_4.3 channels, but not K_V_4.2 channels, without altering their voltage dependence. In additional experiments, both Lgi3 and Lgi4 colocalized with K_V_4.3 with a PCC ∼0.4 in human atrium (see Supplementary material online, *Figure S6E*) and PCC ∼ 0.6 in COS-7 cells (see Supplementary material online, *Figure S6F*). Next, we showed that Lgi4 failed to modify K_V_4.3 expression and cellular distribution (see Supplementary material online, *Figure S7A*), despite their colocalisation with K_V_4.3 (see Supplementary material online, *Figure S7B* and *C*). *I*_ss_, generated by the activation of K_V_2.1, and the inward current, mainly driven by Kir2.1 activation, were not modified in Lgi4 compared to control CMs (*Figure 4C* and *D*). Immunofluorescence assays demonstrated reduced sarcolemmal K_V_1.5 expression in non-permeabilized Lgi4 CMs (*Figure 4E*), which explains the reduction of *I*_Kur_. While Lgi4 did not modify total K_V_1.5 expression, it did shift its cellular distribution from the plasmalemma to intracellular compartments (see Supplementary material online, *Figure S8*).

Previous reports demonstrated that adrenergic stimulation modulates *I*_Kur_ in human atria.^54–57^ Therefore, we compared the effects of isoprenaline on APs and *I*_Kur_ of Lgi4 vs. control ventricular CMs. Supplementary material online, *Figure S9A* shows representative AP traces recorded at 2 Hz before and after 1 μM isoprenaline perfusion for 5–10 min (discontinuous lines), as previously described.^54^ Isoprenaline did not change the RMP, maximal AP amplitude, or upstroke velocity (upper panels). Conversely, early AP repolarisation (APD_50_ to APD_70_) became longer in the presence of isoprenaline in control CMs, but not in Lgi4 CMs (lower panels). At the same time, isoprenaline shifted the plateau potential towards more negative levels in Lgi4 CMs but not in control CMs. Slower repolarisation could be due to an increase in *I*_CaL_ or a decrease in *I*_Kur_. Isoprenaline increases both current amplitudes in human atrial myocytes.^54,58^ However, isoprenaline did not change the *I*_Kur_ amplitude in control CMs, whereas it increased it in Lgi4 CMs (see Supplementary material online, *Figure S9C*), thus explaining the hyperpolarisation of its plateau potential. Since Lgi4 CMs exhibit less K_V_1.5 in plasmalemma, perhaps isoprenaline induces plasmalemmal expression of K_V_1.5, similarly to other ion channels.^59^ These changes in *I*_Kur_ amplitude explain the similarity of isoprenaline effects on APs in control and Lgi4 CMs, as well as the occurrence of EADs (see Supplementary material online, *Figure S9B* and *C*).

### 3.5 Lgi3-4 decrease the amplitude of K_V_1.5/K_V_β current, but not K_V_1.5 alone

**Figure 5:**
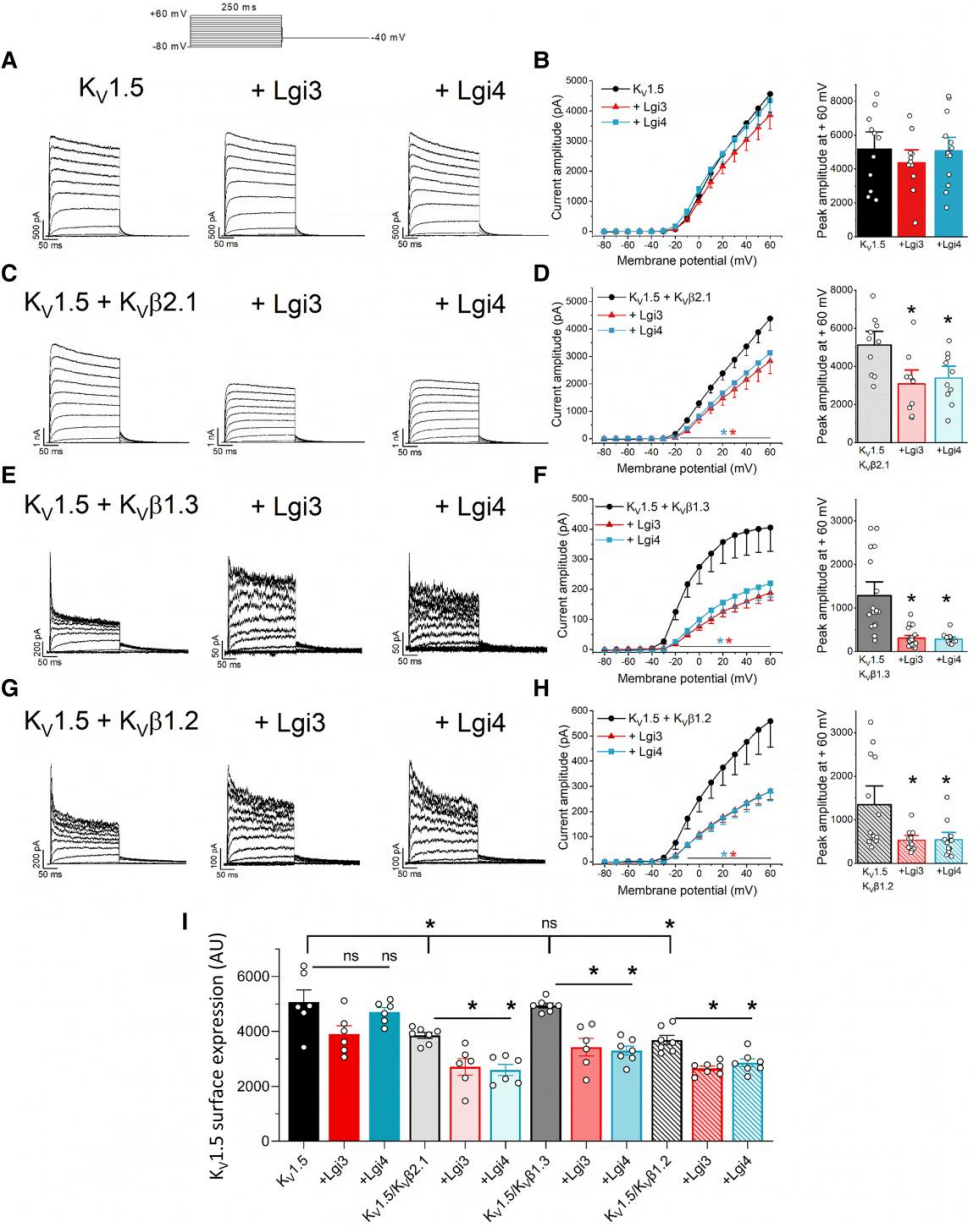
Electrophysiological effects of Lgi3-4 on K_V_1.5 currents. *Left panels*: Representative current traces elicited after the application of the I-V protocol shown in the upper panel in HEK293 cells transfected with K_V_1.5 with or without Lgi3 or Lgi4 (*A*) and in the presence of K_V_β2.1 (*C*), K_V_β1.3 (*E*) or K_V_β1.2 (*G*) subunits. Note that K_V_1.5/K_V_β currents are more similar to K_V_1.5 currents alone when Lgi3 or Lgi4 are expressed. Scale bar: 50 ms in the *x-axis* and the indicated nA or pA in each *y-axis*. *Right panels*: I-V relationships measured at the end of the 250 ms depolarising-pulse and current amplitude at +60 mV measured at the peak in K_V_1.5 with or without Lgi3 (red) or Lgi4 (blue) (n = 11–14) (*B*), and in the presence of: K_V_β2.1 (n = 10) (*D*), K_V_β1.3 (n = 14–24) (*F*) or K_V_β1.2 (n = 10–13) (*H*) subunits. (*I*) Membrane expression of K_V_1.5 in HEK293 cells transfected with K_V_1.5-HA with or without Lgi3 (red) or Lgi4 (blue) and in the presence of K_V_β2.1, K_V_β1.3 or K_V_β1.2 subunits (n = 6–7). Values represent mean ± SEM of the indicated n. (Non-paired *t*-test *P < 0.05).

**Figure 6:**
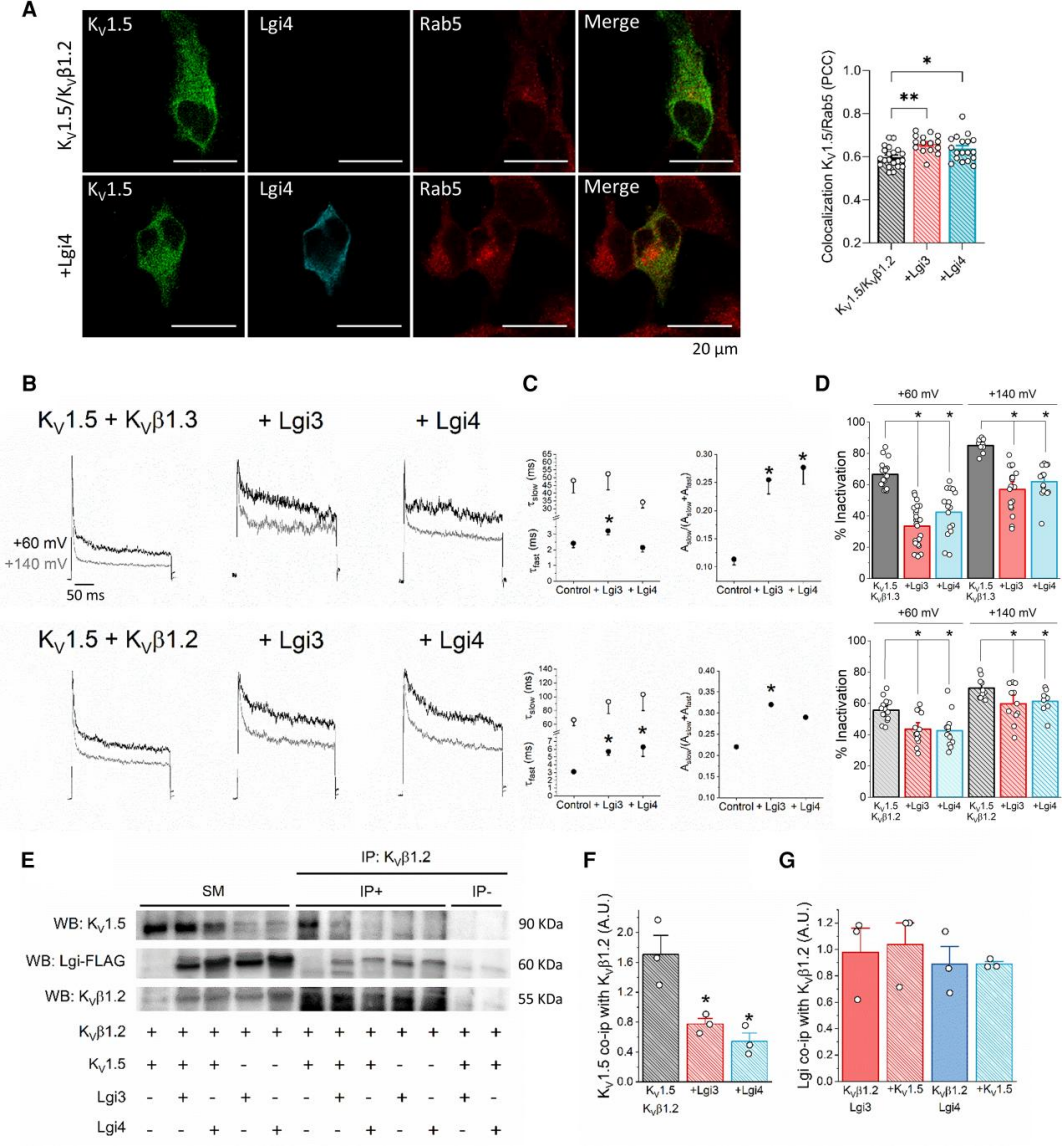
Lgi3-4 increases K_V_1.5 internalisation, decreases the N-type inactivation induced by K_V_β1 and hinders the association between K_V_1.5 and K_V_β. *A*) Representative confocal images of HEK293 cells cotransfected with K_V_1.5 and K_V_β1.2 in the absence (upper panels) or the presence of Lgi4 (lower panels), and stained against Rab5, K_V_1.5 and Lgi4, with the PCC between K_V_1.5 and Rab5 shown in the right bar graph. Data represent mean ± SEM of n = 15–21. Scale bar: 20 µm. *B*) Representative normalized current traces recorded after application of 250 ms depolarising pulses to +60 (black) or +140 mV (grey) in HEK293 cells transfected with K_V_1.5/K_V_β1.3 (upper panel) or K_V_1.5/K_V_β1.2 (lower panel) in the absence and in the presence of Lgi3 or Lgi4. Scale bar: 50 ms. *C*) A double exponential was fitted to the data to obtain the inactivation kinetics measured at +140 mV. The left graphs show the time constant of inactivation, and the right graphs show the contribution of the slow inactivation to the whole inactivation process (n = 8–18). *D*) Degree of inactivation of K_V_1.5 with (+) or without (−) K_V_β1.3, K_V_β1.2, Lgi3 and/or Lgi4 currents at +60 mV (left graph, n = 12–23) and +140 mV (right graph, n = 8–18). Values represent mean ± SEM. A non-paired *t*-test was performed comparing each condition with its corresponding control. *P < 0.05. *E*) HEK293 cells were cotransfected with (+) or without (−) K_V_1.5, K_V_β1.2, Lgi3 and Lgi4. Total lysates were immunoprecipitated (IP) against K_V_β1.2 and immunoblotted (IB) against K_V_1.5, FLAG (Lgi3-4) and K_V_β1.2 (n = 3). *F*) Quantification of K_V_1.5 coimmunoprecipitation with K_V_β1.2 measured as K_V_1.5 expression in the IP+ divided by its corresponding SM expression. Note that K_V_1.5 colocalisation with K_V_β1.2 decreases when Lgi3-4 are coexpressed. *G*) Quantification of Lgi3-4 (FLAG) coimmunoprecipitation with K_V_β1.2 measured as FLAG expression in the IP + divided by its corresponding SM expression. Values represent mean ± SEM of the n indicated above. Non-paired *t*-tewasere performed comparing each condition with its corresponding control (*P < 0.05). SM: starting material, IP+: IP in the presence of antibody, IP-: IP in the absence of antibody.

To investigate the molecular mechanism by which Lgi3-4 proteins modulate *I*_Kur_, we conducted experiments in the simplified HEK293 cell model, expressing Lgi3-4 and co-transfected with K_V_1.5 alone or in the presence of different cardiac K_V_β subunits (K_V_β2.1, K_V_β1.3, and K_V_β1.2). Lgi3 and Lgi4 had no significant effect on the K_V_1.5 current amplitude (*Figure 5A* and *B*). However, when K_V_1.5 channels were assembled with K_V_β2.1, K_V_β1.2 or K_V_β1.3 subunits, Lgi3-4 exerted two striking effects on K_V_1.5 currents: 1) the effects of K_V_β2.1 on C-type inactivation and those of K_V_β1 on N-type inactivation were almost abolished in the presence of Lgi3-4 (see Supplementary material online, *Table S1*, *Figure 5C, E* and *G*), resembling the current elicited by K_V_1.5 channels alone; and 2) the current amplitude of K_V_1.5 coexpressed with K_V_β2.1, K_V_β1.3 and K_V_β1.2 was reduced when Lgi3-4 were expressed, both at the end of the depolarising pulse and at the peak amplitude (*Figure 5D, F* and *H*). The effects on current amplitude could be due to changes in the trafficking of K_V_1.5 channels. Flow cytometry experiments showed that Lgi3-4 decreases the membrane expression of K_V_1.5 in the presence of K_V_β2.1, K_V_β1.2, or K_V_β1.3 (*Figure 5I*), but not in the absence of K_V_β subunits. To explore the underlying mechanism, we studied the colocalisation of K_V_1.5 with different trafficking markers in the presence of K_V_β1.2, with or without Lgi3-4. K_V_1.5 colocalisation with DsRed (ER) (see Supplementary material online, *Figure S10A*) and Rab8 (see Supplementary material online, *Figure S10B*) were not modified by Lgi3-4, indicating that the ER retention and forward trafficking, respectively, remain unaffected.^60^ In contrast, Lgi3-4 increased the colocalisation of K_V_1.5 and the early endocytosis marker Rab5^60^ (*Figure 6A*), thus suggesting that Lgi3-4 prompted endocytosis of K_V_1.5 channels. Notably, colocalisation of K_V_1.5 with the recycling endosome marker Rab11^60^ was unaltered (see Supplementary material online, *Figure S10C*), indicating that the recycling efficiency was not impaired by Lgi3-4. Consequently, the ratio of endocytosis to recycling increased in the presence of Lgi3-4, consistent with enhanced internalisation of K_V_1.5 channels. These results could partially explain the reduction in the current amplitude. Another possibility could be a shift in the voltage dependence of activation towards more positive potentials. Lgi4, but not Lgi3, shifted K_V_1.5 V_1/2_ of activation towards more negative potentials. For K_V_1.5/K_V_β2.1 currents, either Lgi3-4 shifted the V_1/2_ towards more positive potentials, which is in agreement with a reduction in the current amplitude. Neither Lgi3 nor Lgi4 changed the V_1/2_ of K_V_1.5/K_V_β1.3 and K_V_1.5/K_V_β1.2 currents (see Supplementary material online, *Table S2*).

### 3.6 Lgi3-4 reduce K_V_β-induced inactivation through competitive binding

As stated above, Lgi3-4 reduce the effects of K_V_β subunits on the inactivation of K_V_1.5 channels. To better understand, we employed various electrophysiological protocols, taking into account that N-type inactivation is highly voltage-dependent.^14,61^ First, we applied depolarising pulses at +60 and at +140 mV (*Figure 6B*). The traces elicited at +140 mV enabled us to analyse the kinetics of inactivation, obtaining the time constants (τ_fast_ and τ_slow_, for the fast or N-type and slow or C-type inactivation, respectively), and the relative contribution of the slow component of inactivation (A_slow_), which corresponds to the A_slow_/(A_slow_ + A_fast_).

We tested whether Lgi3 and Lgi4 decrease the contribution of the N-type inactivation either by: 1) slowing τ_fast_ or 2) increasing the A_slow_ contribution. Neither Lgi3 nor Lgi4 modified the τ_slow_ values in any experimental condition (*Figure 6C*). Lgi3-4 clearly reduced the degree of inactivation of K_V_1.5/K_V_β1.3 and K_V_1.5/K_V_β1.2 currents at either membrane potential (*Figure 6D*). These effects can be explained if: 1) Lgi3-4 shift the voltage dependence of N-type inactivation to more positive potentials, or 2) Lgi3-4 hinder the interaction of K_V_1.5 with K_v_β1. To test the first hypothesis, we analysed the effects of Lgi3-4 on the voltage-dependence of N-type inactivation induced by K_V_β1.3 or K_V_β1.2, which was only slightly modified (see Supplementary material online, *Table S3*, Supplementary material online, *Figure S11*). The degree of inactivation accumulated during the 10-ms prepulse decreased strongly in the presence of Lgi3 or Lgi4, which was consistent with the above-mentioned reduced degree of N-type inactivation. Next, we conducted co-immunoprecipitation experiments to explore whether the effects of Lgi3-4 on K_V_1.5/K_V_β1 currents were due to competition between these distinct proteins. As shown in *Figure 6E* and *F*, K_V_1.5 coimmunoprecipitation with K_V_β1.2 was dramatically reduced in the presence of Lgi3-4. Additionally, Lgi3-4 interacted with K_V_β1,.2 and the presence or absence of K_V_1.5 did not alter this interaction (*Figure 6E* and *G*). Finally, the presence of K_V_β1.2 did not modify the degree of co-immunoprecipitation between K_V_1.5 and Lgi3-4 FLAG-tagged (see Supplementary material online, *Figure S11C* and *D*). The above results suggest that Lgi3-4 compete with K_V_β1.2 for its binding to K_V_1.5 channels; and also, that Lgi3-4 bind to both K_V_1.5 and K_V_β1.2.

Additionally, as Lgi’s effects in neurons are mainly mediated by ADAM receptors, we studied the effects of Lgi3-4 on K_V_1.5/K_V_β1.3 currents in the presence of ADAM23, which is expressed in cardiomyocytes. We obtained similar effects to those in the absence of ADAM23, thus suggesting that the effects of Lgi3-4 in cardiac K_V_1.5 channelosome are independent of ADAM23 (see Supplementary material online, *Figure S12*).

### 3.7 The K_V_1.5 channelosome is altered in atrial samples from patients with atrial fibrillation

**Figure 7:**
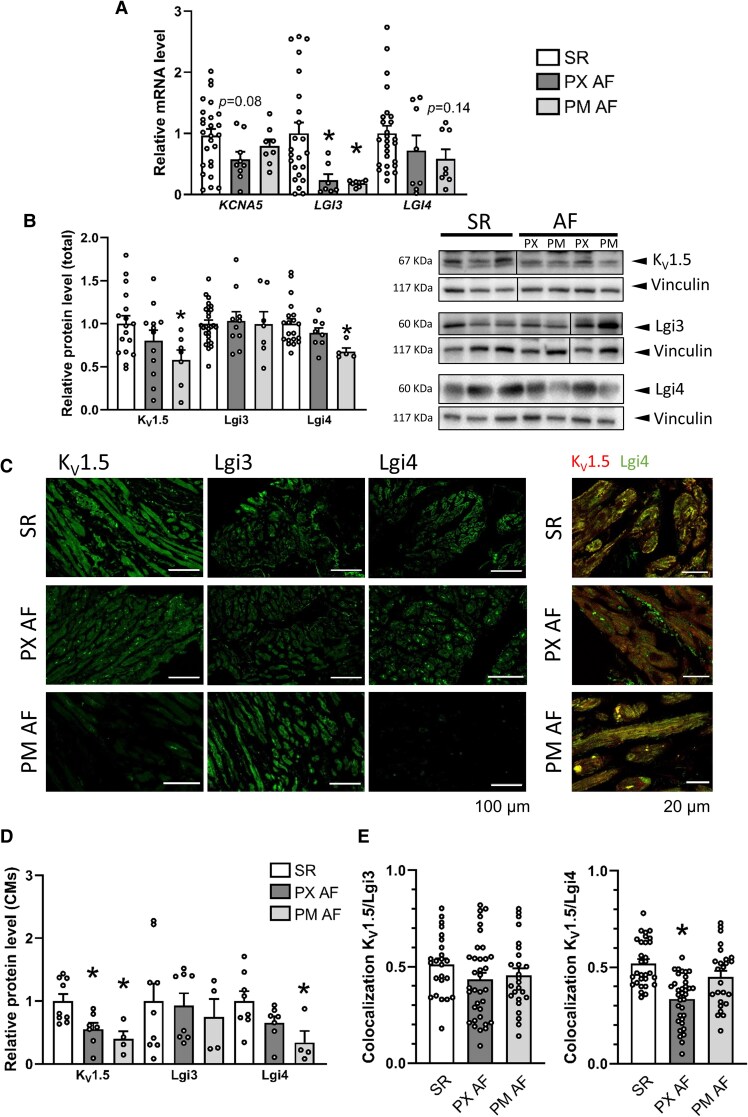
Changes in the expression of the K_V_1.5 channelosome in samples from patients in atrial fibrillation (AF) compared to those in sinus rhythm (SR). *A*) Relative mRNA expression of *KCNA5*, *LGI3* and *LGI4* in right atrial tissue from patients in SR and with AF determined with qPCR. AF patients are segregated on account of the progression of AF into paroxysmal (PX) and permanent (PM) AF. *B*) Relative protein expression of K_V_1.5, Lgi3 and Lgi4 in right atrial tissue from patients in SR and with PX or PM AF probed in western-blot with anti-K_V_1.5, anti-Lgi3 or anti-Lgi4 antibodies and normalized by vinculin expression. Representative immunoblots using anti-K_V_1.5, anti-Lgi3, anti-Lgi4 or anti-Vinculin are shown on the right. Note that the lanes were run on the same gel but were noncontiguous. *C*) *Left*- Representative confocal images of the immunodetection of K_V_1.5, Lgi3 and Lgi4 in the right atria of patients in SR (upper panels) and with AF (lower panels). Scale bar: 100 µm. *Right-* Representative double immunofluorescence images of K_V_1.5 and Lgi4 in SR and PX AF samples. Scale bar: 20 µm. *D*) Relative protein expression of K_V_1.5, Lgi3 and Lgi4 in atrial myocytes from patients in SR and with AF measured in individual cardiomyocytes from confocal images obtained at 40X. Each point represents the mean of 50–100 CMs from two independent IF for each patient. *E*) Colocalisation of K_V_1.5 and Lgi3 (left) or Lgi4 (right) in atrial myocytes from patients in SR, PX AF or PM AF measured in individual cardiomyocytes from confocal images obtained at 63X. One-way ANOVA with Tukey test was performed when comparing more than two groups. *P < 0.05.

To study if the K_V_1.5 channelosome was dysregulated in AF, human samples from the right atrial appendage were collected from patients in SR (control) and patients with PX and PM AF. Supplementary material online, *Table S4* shows the information on the patients included in the study. *KCNA5* mRNA levels were decreased in both groups of AF patients (see Supplementary material online, *Figure S14A*), but the reduction was greater in PX than PM AF patients (*Figure 7A*). Compared with SR, *LGI3* mRNA levels were low in PX and PM AF patients, but *LGI4* mRNA showed only a tendency to reduction in both AF groups (*Figure 7A*). A positive correlation between *LGI3*/*KCNA5*, *LGI4*/*KCNA5* and *LGI3*/*LGI4* mRNA expressions was observed (see Supplementary material online, *Figure S14D*). Either the total protein expression of K_V_1.5, Lgi3 and Lgi4 in atrial homogenates from patients in SR or AF (*Figure 7B*, Supplementary material online, *Figure S13B*) or the expression of these proteins in cardiomyocytes (*Figure 7C left*, *7D*, Supplementary material online, *Figure S13C*) followed the same trend. K_V_1.5 and Lgi4 protein expression was reduced in atrial homogenates from patients in PM AF, but not or only slightly in PX AF; whereas Lgi3 protein expression was not modified in any case (*Figure 7B*). Both Lgi3 and Lgi4 protein expression exhibited a correlation trend with K_V_1.5 protein expression (see Supplementary material online, *Figure S13E*). Unexpectedly, Lgi3 and Lgi4 had a positive correlation (see Supplementary material online, *Figure S13E*). More surprisingly, compared to control, the colocalisation between K_V_1.5 and Lgi4 was reduced in atrial samples from PX but not PM AF patients, which might contribute to fine-tune the mechanisms involved in the AF electrical remodelling (*Figure 7C right*, *7E*).

## 4. Discussion

This is the first study to examine Lgi protein expression and function in the cardiovascular system. We identified Lgi3-4 as new K_V_1.5 channelosome members that modulate its trafficking and biophysical properties, influencing cardiac electrophysiology. These findings expand our understanding of the physiological roles of these proteins and the macromolecular complexes that regulate *I*_Kur_, a key atrial repolarising current involved in diseases such as atrial fibrillation.

The association of Lgi proteins with K_V_1 and K_V_4 channels in neurons is well established, and it has been shown that Lgi3 is linked to the trafficking of K_V_1 channels in these cells.^24,27–29,38^ We demonstrate that Lgi3 and Lgi4 are the only members of the Lgi family that are significantly expressed in human right atrial and ventricular CMs. This finding follows a previous proteomic analysis that identified Lgi1, Lgi2, and Lgi4 among the 75% most abundant proteins in the human heart.^62^ However, in that study, Lgi1 and Lgi2 were detected only in the left atrium, but not in the right atrium or ventricles. Additionally, despite proteomics being a sensitive technique, it can lead to false positives; thus, findings from proteomic studies often require further validation with techniques that use specific antibodies. Furthermore, we found that Lgi3 and Lgi4 interact with K_V_1.5 and K_V_4.3 channels in both human atria and mouse ventricles, as well as in heterologous systems. These interactions result in a reduction of K_V_1.5 current amplitude and membrane expression when K_V_β subunits are present, observed in both atrial and ventricular mouse CMs and in HEK293 cells.

Analysis of lead-II ECGs in anaesthetized Lgi4 mice revealed a lengthening of the QRS complex while other parameters (P wave, RR, PR, and QTc duration) were unaltered when compared to WT. In humans, the QRS complex duration depends on the rate of intraventricular conduction, primarily determined by the upstroke velocity of the AP (mainly due to the *I*_Na_ density). However, in mice with much shorter APDs, the QRS interval encompasses membrane depolarisation (*I*_Na_) and part of the early repolarisation (*I*_Kur_ and *I*_to_) of the ventricle.^51^ Therefore, the slower early repolarisation observed in isolated Lgi4 CMs may, at least in part, explain the longer QRS interval on the surface ECG.

Although the average RR interval was unaltered at baseline, it was more variable in Lgi4 mice than in control mice, suggesting a possible alteration at the level of the SAN. In addition, the increased SNRT that followed right atrial PES suggested that SAN function was modified by Lgi4 expression, or that the atrial refractory period was prolonged, potentially due to the prolongation of the atrial APD. Moreover, PR interval prolongation and concurrent intermittent AV block after high-frequency ventricular stimulation revealed a conduction alteration either at the AV node and/or the His-Purkinje system that was not present at baseline. SAN and AVN cells express K_V_1.5,^63–65^ and a reduction in *I*_Kur_ in SAN and AVN decreases the firing frequency of these structures,^63^ which explains, at least in part, why Lgi4-induced *I*_Kur_ reduction may have an impact on sinoatrial and AV nodal conduction, while no atrial or ventricular tachyarrhythmias were reported when expressing Lgi4.

AP repolarisation in atrial Lgi4 cardiomyocytes (CMs) was significantly slower than in controls. In ventricular CMs, Lgi4 expression only mildly slowed early repolarisation. In both cell types, the slowing increased progressively with higher stimulation frequencies, resulting in gradual action potential (AP) prolongation when pulse trains above 4 Hz were applied. In many CMs, this excessive AP prolongation led to the formation of early afterdepolarisations (EADs) in both the atria and ventricles. In mouse cardiomyocytes, AP repolarisation depends on the balance of outward and inward currents, primarily established by *I*_to_, *I*_Kur_, and *I*_Ca, L_ at each phase.^66,67^ In our experiments, *I*_to_, which is conducted by K_V_4.3/K_V_4.2 heterotetramers, was not significantly altered in Lgi4 CMs. This may be due to the predominance of K_V_4.2 channels in mouse CMs, which, unlike K_V_4.3, are not regulated by Lgi3-4. Other modulatory subunits of K_V_4.3/K_V_4.2, such as KChIP2, DPP6, K_V_β2.1, and/or KCNE2,^68,69^ which are expressed in CMs but absent in CHO cells, might also contribute. The differential modulation of K_V_4.3 and K_V_4.2 by Lgi3-4 may involve distinct mechanisms: (1) direct modulation of the ion channels, or (2) alternative regulatory pathways, such as CaMKII, which modulates K_V_4.3 but not K_V_4.2.^70^ However, these possibilities are beyond the scope of this study.

The slower repolarisation and depolarized AP plateau potential in Lgi4 CMs can be attributed to the reduction in *I*_Kur_, which is more pronounced in atrial than ventricular CMs, consistent with the greater atrial APD prolongations. These results align with the heterologous expression experiments, as mouse CMs express the K_V_β2.1 and K_V_β1.2 subunits.^16,71^ The depolarized plateau potential can be exacerbated by a predominance of depolarising *I*_Ca, L_ secondary to *I*_Kur_ block, as previously described by Wettwer *et al*. (2004) for K_V_1.5 blockers, which do not directly affect *I*_Ca, L_.^67^

Isoprenaline activates β-adrenergic receptors, boosting systolic calcium and K^+^ currents to strengthen heart contractions while limiting the duration of the Aps.^59^ In human atria, isoprenaline increases *I*_Kur_,^54–57^ similarly to when K_V_1.5 is coexpressed with K_V_β1.3. Mouse cardiomyocytes, however, lack K_V_β1.3, which may explain why isoprenaline does not affect *I*_Kur_ in WT cells.^72^ In ventricular Lgi4 CMs, isoprenaline increased I_Kur_, likely by moving K_V_1.5 channels to the plasma membrane. This is similar to the rapid translocation of K_V_7.1 seen with isoprenaline in human induced-pluripotent CMs.^59^ This mechanism may explain why isoprenaline reverses Lgi4-induced reduction in K_V_1.5 current, restoring *I*_Kur_ and balancing the isoprenaline-driven increase in *I*_CaL_.^59^ As a result, total AP duration (APD) does not change, but the AP plateau potential becomes more negative. In control CMs, isoprenaline does not increase *I*_Kur_, so *I*_CaL_ effects are unopposed, and AP duration is prolonged. Thus, AP morphology is similar between control and Lgi4 CMs only when isoprenaline is present.

Lgis reduce K_V_1.5 membrane expression in both cardiomyocytes and HEK293 cells. Ion channel trafficking is tightly modulated to ensure proper channel function. K_V_1 channels are folded, glycosylated and assembled in the ER, and then translocated to the Golgi apparatus, where they mature before being transported to the sarcolemma.^60,73,74^ This process represents the primary limiting step for trafficking/expression of most membrane proteins, and it is enhanced by K_V_β subunits for some K_V_1 channels.^15,75,76^ Recent research demonstrated that K_V_β2 subunits increase *I*_Kur_ and K_V_1.5 membrane expression in native cardiomyocytes,^16^ similarly to K_V_β1.2 in coronary arterial myocytes.^77^ However, Lgi3-4 did not produce changes in the forward trafficking of K_V_1.5 channels in the presence of K_V_β1.2 subunits. Also, the membrane expression of K_V_1 channels is modulated by internalisation and intracellular retention.^74,78^ On this basis, Lgi3-4 enhanced the endocytosis of plasmalemmal K_V_1.5 channels, decreasing their membrane expression and current amplitude. These channels were probably accumulated in early endosomes, as their recycling was not increased, nor was their degradation, as the total K_V_1.5 expression was not altered. Nevertheless, further research is needed to decipher how Lgi3-4 influences these processes.

Additionally, the reduced K_V_1.5/K_V_β interaction produced by Lgi3-4 also explains the abolishment of the N-type inactivation induced by K_V_β1 subunits on K_V_1.5, as previously reported for K_V_1.1/K_V_β1.^27^ However, Lgi3-4 does not alter the K_V_β1-induced changes in the voltage dependence of K_V_1.5 activation and deactivation kinetics. This indicates that the interaction between K_V_1.5/K_V_β1 is sufficient to produce those effects exerted through the C-terminus of the K_V_β1, but not those induced through the K_V_β1 N-terminus. Similar patterns were observed with K_V_β1.3 N-terminus mutants and mutants in the S6 of K_V_1.5. Those variants retained their ability to cause negative shifts in K_V_1.5 activation and inactivation, even when the degree of inactivation was modified, suggesting that these gating effects were mediated by interactions other than those affecting N-type inactivation.^79,80^ Perhaps, in addition to a decreased interaction between K_V_1.5 and K_V_β subunits, the interaction of Lgi proteins with K_V_1.5 and/or K_V_β subunits hinders the introduction of the K_V_β1 N-terminus into its binding site in the K_V_1.5 pore. Similarly, in the presence of Lgi3-4, K_V_1.5/K_V_β2.1 currents are closer to those of K_V_1.5 alone, decreasing the C-type inactivation and shifting the midpoint of the voltage-dependence of activation towards more positive potentials. This reverses part of the effects induced by K_V_β2.1, but not those on the deactivation kinetics or the voltage dependence of inactivation.

As previously discussed, Lgi3-4 interact with cytoplasmic K_V_β subunits, modulating their effects on K_V_1.5 channels intracellularly. This is similar to findings by Schulte *et al*., who demonstrated that Lgi1 abolishes the N-type inactivation induced by the K_V_β1 subunit on K_V_1.1, but not the intrinsic inactivation of K_V_1.4.^27^ In contrast to many studies in the nervous system, in which secreted Lgis mediate their effects primarily via interaction with members of the ADAM family (ADAM11, 22 and 23),^23,24,28,34,81,82^ we found no evidence that cardiac Lgi3-4 modulation of K_V_1.5/K_V_β currents involved ADAM23 despite its cardiac expression. Interestingly, ADAM23 and K_V_β proteins share some structural hallmarks, both being globular proteins with α-helical structures. This may explain the interaction of Lgi proteins with K_V_β subunits, presumably through the EPTP domain of Lgi similar to ADAM23. It also explains why ADAM23 does not induce changes in K_V_1.5/K_V_β currents produced by intracellular Lgi protein binding to K_V_1.5 and K_V_β.

Numerous ion channels and modulatory subunits are dysregulated in AF, contributing to the electrical remodelling of the atria, which includes shortening of the APD and effective refractory period, and a lack of adaptation of APD to fast heart rates.^1,3,5,11,83–85^ We studied the expression of members of the K_V_1.5 channelosome in right atrial appendages of patients in SR (control) and two stages of AF progression (PX and PM). *KCNA5* mRNA and K_V_1.5 protein levels were reduced in atrial appendages from AF vs. SR patients,^8,86^ with a more pronounced decrease in PM than PX AF, as previously reported.^5,8,87^ This follows the same trend as the reported reduction in *I*_Kur_ in human atrial CMs from patients in chronic AF.^9^  *LGI3* mRNA expression is decreased in AF in comparison to SR, but Lgi3 protein levels are not modified at any AF stage. *LGI4* mRNA is reduced in AF, and this downregulation translated into decreased Lgi4 protein levels in PM -but not in PX- AF. This discrepancy between mRNA and protein contents, especially in the case of Lgi3, may reflect post-transcriptional regulatory mechanisms. Protein levels are usually more conserved than mRNA amount, and changes in transcription can be associated with translational changes with opposite effects, producing a buffering effect.^88^ Moreover, the positive correlation between Lgi3 and Lgi4 may suggest that they do not compensate for each other when one of them is downregulated. In addition, we observed a reduced Lgi4-K_V_1.5 colocalisation in PX AF, but not in PM AF. This might reflect either a reduction in their interaction or changes in the abundance of one of these proteins. It is notable that despite reduced K_V_1.5 protein expression in PX AF, there is no electrical remodelling at early stages of the disease.^89^ This could be explained by a reduction in Lgi4-K_V_1.5 interaction, which would lead to more K_V_1.5 available at the membrane not interacting with Lgi4, partially compensating for the lower total K_V_1.5 expression. In contrast, in PM AF, both K_V_1.5 and Lgi4 are reduced, but their interaction remains similar to that in SR, leading to a net reduction in *I*_Kur_.

AF is a complex and multifactorial disease in which several genes and signalling pathways are altered; therefore, it is challenging to determine whether the regulation of K_V_1.5 and Lgi4 is a cause, a consequence, a compensatory mechanism, or even unrelated to AF. We can analyse the results and speculate. Our data show that Lgi4 decreases *I*_Kur_ without affecting total K_V_1.5 expression. Thus, a reduction of Lgi4 could theoretically increase *I*_Kur_ as a compensatory mechanism in AF to counter the downregulation of K_V_1.5. However, this compensatory response appears insufficient, as *I*_Kur_ is decreased in chronic AF.^9^ Moreover, this compensation might ultimately be maladaptive, contributing further to APD shortening and atrial arrhythmogenicity.

From a therapeutic standpoint, enhancing Lgi4 expression or function may represent a novel strategy to selectively further reduce *I*_Kur_ and prolong atrial APD. Given that Lgi4 has greater effects on atrial than ventricular repolarisation in mice, and that human ventricle lacks *I*_Kur_, such an approach could provide atrium-specific modulation. We expect that increasing Lgi4 expression or function mimics K_V_1.5 channel blockade. In remodelled (PM) atria, it would increase the APD_90_; whereas in non-remodelled (PX) atria, it would prolong the AP at higher rates (4 Hz and higher), thus suppressing APs and reducing the heart rate.^11,89,90^ However, this is speculative and would require demonstration.

Taken together, our findings identify Lgi3-4 as novel modulatory subunits of the K_V_1.5 channelosome, with key roles in shaping cardiac repolarisation. Their differential expression and interaction with K_V_1.5 in AF suggest a potential for atrial-selective therapeutic targeting. The modulation of K_V_1.5 by Lgi proteins opens up new pharmacological avenues that warrant further investigation, both for understanding atrial remodelling and for developing more precise treatments for atrial fibrillation.

## 5. Limitations

While our study provides novel insights into the electrophysiological role of Lgi4 in the heart, several potential limitations should be considered. First, we studied Lgi4 function in mouse cardiomyocytes. Mouse AP characteristics are different from those in humans, and they exhibit a large *I*_Kur_.^91,92^ In addition, the role of Lgi4 may vary between mammalian species. Thus, our results may not accurately reflect the physiological interactions of Lgi4 in human. Regarding the use of human samples, we only had samples from the right atrium and ventricle, and we did not have access to fresh human tissue to characterize the electrophysiological properties of isolated cardiomyocytes. Additionally, further experiments will be necessary to determine whether the Lgi4-induced increase in K_V_1.5 at the plasmalemma was due to a rise in forward trafficking or a decrease in the recycling of the channels. This study is well beyond the scope of this study. Lastly, the isoprenaline experiments were performed only in ventricular cardiomyocytes because they exhibit *I*_Kur_ and are easier to obtain than atrial cardiomyocytes. Although no extrapolations would be possible, it would be more rigorous to perform the experiments in atrial cardiomyocytes, as human *I*_Kur_ is present only in the atria. However, the effects of Lgi4 on *I*_Kur_ in atria and ventricles go in the same direction, even though they are greater in the atria. Despite the above limitations, our findings establish that Lgi proteins interact with cardiac K_V_1.5 and alter *I*_Kur_ properties, which might have important clinical implications for the physiopathology cardiac arrhythmias like AF and may have therapeutic implications.

Translational perspective Our findings identify Lgi3-4 as previously unrecognized modulatory subunits of the K_V_1.5 channelosome, suggesting a potential as atrial-selective therapeutic targets. Lgi3-4 decreases *I*_Kur_ by prompting endocytosis of K_V_1.5, and modulates its inactivation by hindering K_V_1.5/K_V_β interaction, shaping atrial repolarisation—an electrophysiological substrate central to AF. The altered expression of Lgi4 in AF suggests that targeting this protein could enable the modulation of atrial electrophysiology, while minimising ventricular effects, addressing a major limitation of current antiarrhythmic therapies. These results open up novel strategies that warrant further investigation, both for understanding atrial remodelling and for developing more precise treatments for AF.

## Supplementary Material

cvag049_Supplementary_Data
