# Supplementary material for: Lgi3-4 proteins modulate the KV1.5 channelosome and are potential therapeutic targets for atrial fibrillation
Source: Cardiovasc Res. 2026 Mar 19;122(7):874–91. doi: 10.1093/cvr/cvag049 (PMC13196873; doi:10.1093/cvr/cvag049)
Supplement: cvag049_Supplementary_Data [file cvag049_supplementary_data.docx]

**Supplementary information**

**Novel insights into *I*_Kur_ modulation by Lgi3-4: Implications in atrial fibrillation**

Paula G. Socuéllamos^1,2*^, Álvaro Macías^2^, Ángela de Benito-Bueno^1^, Francisco M. Cruz^2^, María Redondo-Moya^1^, María José Coronado^3^, Elvira Ramil^3^, Silvia Rosado^3^, Elsa Carolina Rios-Rosado^4^, María Valencia-Avezuela^1^, Laura Andrés-Delgado^5^, José Antonio Blázquez^6,7^, Alberto Forteza-Gil^4^, Marta Gutiérrez-Rodríguez^8^, José Jalife^2,9,10^ & Carmen Valenzuela^1,10*^.

^1^ Instituto de Investigaciones Biomédicas Sols-Morreale CSIC-UAM, Madrid, Spain.

^2^ Centro Nacional de Investigaciones Cardiovasculares (CNIC), Madrid, Spain.

^3^ Institute of Health Research Puerta de Hierro- Segovia de Arana, IDIPHISA, Madrid, Spain.

^4^ Hospital Universitario Puerta de Hierro, Madrid, Spain.

^5^ Departamento de Anatomía, Histología y Neurociencias, Facultad de Medicina, Universidad Autónoma de Madrid, Madrid, Spain.

^6^ Departamento de Cirugía Cardiaca, Hospital Universitario La Paz, Madrid, Spain.

^7^ Complejo Asistencial Universitario de Salamanca, Salamanca, Spain.

^8^ Instituto de Química Médica (IQM-CSIC), Madrid, Spain.

^9^ Departments of Internal Medicine and Molecular and Integrative Physiology, University of Michigan, Ann Arbor, MI, USA.

^10^ Centro de Investigación Biomédica en Red de Enfermedades Cardiovasculares (CIBERCV).

**Short title:** Lgi proteins’ role in the pathophysiology of the heart.

*** Corresponding authors:**

Carmen Valenzuela, PhD.

Instituto de Investigaciones Biomédicas Sols-Morreale CSIC-UAM, Madrid, Spain.

Arturo Duperier, 4, 28029 Madrid, Spain

Email: cvalenzuela@iib.uam.es

Paula G. Socuéllamos, PhD.

Centro Nacional de Investigaciones Cardiovasculares (CNIC)

Melchor Fernández Almagro 3, 28029 Madrid, Spain

Email: paula.garcia@cnic.es

DETAILED METHODS

Sex as a biological variable

Human cardiac samples from patients in SR, AF PX and AF PM were included from both sexes, but we did not include sex as a variable in the analysis because we could not assure an equal distribution due to the small number of samples in each group. Regarding mouse models, our study examined male mice because male animals exhibited less variability in phenotype.

Cell culture, plasmids and transfection

All cell lines were obtained from the American Type Culture Collection (Rockville, MD, US) and they were growth at 37 ºC in different culture mediums under a 5% CO_2_ humidified atmosphere. The cell culture medium was changed every 2-3 days and the cells were treated to be reseed (TrypLE^TM^ Express; Life Technologies) every 4-5 days. HEK293, COS7 and *Ltk*- cells were cultured in supplemented DMEM (with 10% foetal bovine serum [FBS] and antibiotics [100 U/ml penicillin and 100 μg/ml streptomycin; all from Gibco, Paisley, UK]). HEK293 cells were used in electrophysiology experiments for K_V_1.5 channels due to its lack of K_V_β subunits ^1^, COS-7 cells were used for immunofluorescence assays, as they constitute an stablished model in cellular biology. The *Ltk^−^* cell line stably transfected with the gene encoding K_V_1.5 channel were cultured in supplemented DMEM with 0.25 mg/ml G418 (Roche, 04727878001), and K_V_1.5 expression was induced with 2 µM dexamethasone (Sigma-Aldrich) for 24 h ^1^. This cell line was used for the recording of K_V_1.5/K_V_β2.1 currents experiments. The Chinese Hamster Ovary CHO-K1 cells (CHO) were cultured in Iscove’s modified Eagle’s medium supplemented with 10% (v/v) FBS, 1% (v/v) L-Glutamine, and antibiotics (100 U/ml penicillin and 100 μg/ml streptomycin; all from Gibco, Paisley, UK). CHO-K1 cells were used in experiments with K_V_4.3 and K_V_4.2 channels due to its lack of endogenous potassium currents.

For electrophysiological and flow cytometry experiments, the following constructions were used: Kv1.5 cloned in pRRL CMV-IRES-GFP ^2^; the bicistronic K_V_β1.3 IRES K_V_1.5 construction cloned in pBK-CMV ^3^; K_V_β1.2-pBK-CMV; K_V_β1.3-pCMV-Tag5A ^4^; K_V_4.3-pBK-CMV vector ^5^; the K_V_4.2-GFP plasmid; and the K_V_1.5-HA ^6^. Lgi3 (NM_139278) and Lgi4 (NM_139284) were subcloned in the bicistronic vector pRR-CMV-IRES-mCherry by introducing *XhoI* and *XbaI* restriction sites using the primers described in Table 1; ADAM23 was cloned into pRR-CMV-IRES-GFP by introducing *XbaI* and *SmaI* restriction sites with the primers described in Table 1.

For immunofluorescence and coimmunoprecipitation experiments: K_V_1.5-GFP pBK vector; K_V_1.5 in pcDNA3.1 vector; K_V_4.3 pmCherry-C1^2^; DsRed in pCAG; Lgi3 pCMV6-Myc-FLAG; Lgi4 pCMV6 Myc-FLAG generated by subcloning Lgi4 into FLAG Myc pCMV6 by introducing *AsiS* and *MluI* sites with the primers described in Table 1. To introduce Lgi4 in adenoassociated viruses (AAVs), Lgi4 was cloned into pAAV-cTnT-IRES-Tomato, and ITR sequences flanking the sequences of interest to enable the packaging in AAV9 ^7^. The primers described in Table 1 were used in order to introduce the necessary restriction sites (*BamHI* and *NotI*). The presence of ITR was checked out by digestion with *AhdI* and *BglI*. All cDNA constructions were checked by sequencing.

Table 1. Primers used to generate the constructs. Restriction sites are shown in bold.

| **Construction** | **Fw (5’ – 3’)** | **Rv (5’ – 3’)** |
| --- | --- | --- |
| Lgi3 IRES mCherry | ATA**TCTAGA**ATGGCGGGGCTGCGGGC | CC**CTCGAG**CTAGGCACTGAGATCCACCACAAT |
| Lgi4 IRES mCherry | CGC**TCTAGA**ATGGGAGGGGCAGGCATTC | ATA**CTCGAG**AGTTCAGGCACTGAGGTCGATCTC |
| Lgi4 FLAG Myc pCMV6 | GCCGCC**GCGATCGC**CATGGGAGGGGCAGG | CGGCCGCGT**ACGCGT**GGCACTGAGGTCGATCTC |
| Lgi4pAAV IRES Tomato | TTGCTA**GGATCC**CCCATGGGAGGGGCAGG | CCGTCGA**GCGGCCGC**AGTCAGGCACTGAGG |
| ADAM23 IRES GFP | ATA**TCTAGA**ACCATGAAGCCGCCCGGCGC | **CCCGGG**TCAGATGGGGCCTTGCTGAGTAGG |

All transient transfections in HEK293, *Ltk*^-^ and COS7 were carried out using Lipofectamine 2000® (11668027, Invitrogen). CHO-K1 cells were transfected with Fugene® 6 (E2691, Promega). The transfections were carried out following manufacturer’s instructions and the cDNA amount and transfection conditions are specified in each technique (see below).

Mice

5-6-weeks-old C57BL/6J mice were obtained from the Charles River Laboratories. Mice were reared and housed in accordance with institutional guidelines and regulations. The mice had free access to food and water. Animal protocols were carried out in accordance with the Centro Nacional de Investigaciones Cardiovasculares (CNIC) Institutional Ethics Committee recommendations and were approved by the Animal Experimentation Committee (Scientific Procedures) of Comunidad de Madrid (PROEX 111.4/20 and PROEX 226.5/23).

Human samples

The investigation conforms to the World Medical Association Declaration of Helsinki principles of medical research involving human subjects.

Cardiac samples of individuals without cardiovascular histories were obtained from autopsy procedures at Hospital Universitario la Paz (Madrid). Fully written informed consents were obtained from the families of the donors. The paraffin-embedded tissue sections were kindly provided by Dr. J. Regadera (Universidad Autónoma de Madrid). The study was approved by the respective institutional review boards and ethic committees.

Human right atrial appendage cardiac samples were provided by the Cardiac Surgery Service of University Hospital La Paz-IdiPAZ and University Hospital Puerta de Hierro. We obtained these samples from patients under sinus rhythm (SR) and from patients with atrial fibrillation (AF). All samples involved in this study were obtained with the appropriate informed consent and their use was approved by the Ethics Committee of the University Hospital La Paz (PI-2550), the Ethics Committee of the University Hospital Puerta de Hierro (PI-158-22) and the Ethics Committee in Human and Animal experimentation CEEHA and the Biosecurity committee of the CSIC (PI-2550 and 046/2023). In addition, all procedures were done accordingly with the 1527/2010 (November 15th, 2010) and the 1716/2011 (November 18th, 2011) Royal Decrees, as well as the 9/2014 (July 4th, 2014) Royal Decree-Law. These cardiac samples were used for mRNA and protein quantification, as well as for immunofluorescence and confocal imaging.

Adeno-associated virus (AAV) vector production and purification

The corresponding plasmids were packed in AAV9 capsides given its proved efficacy for the release of genes in mice cardiomyocytes ^8–10^. AAV vectors were produced by the triple transfection method, using HEK293T cells by the CNIC’s Viral Vectors Unit as described previously ^10,11^. AAV plasmids were cloned and propagated in the Stbl3 *Escherichia coli* strain (Life Technologies). Shuttle plasmids pAAV-Tomato and pAAV-Lgi4-IRES-Tomato were packaged into AAV-9 capsids with the use of helper plasmids pAdDF6 (providing the three adenoviral helper genes) and pAAV2/9 (providing rep and cap viral genes), obtained from PennVector.

The AAV shuttle and helper plasmids were transfected into HEK293T cells by calcium-phosphate co-precipitation. A total of 840 µg of plasmid DNA (mixed in an equimolar ratio) was used per HYPERFlask (Corning) seeded with 1.2 × 10^8^ cells the day before. Seventy-two hours after transfection, the cells were collected by centrifugation, and the cell pellet was resuspended in TMS (50 mM Tris HCl, 150 mM NaCl and 2 mM MgCl_2_) on ice before digestion with DNase I and RNaseA (0.1 mg/ml each; Roche) at 37 °C for 60 minutes. Clarified supernatant containing the viral particles was obtained by iodixanol gradient centrifugation ^12^. Gradient fractions containing virus were concentrated using Amicon UltraCel columns (Millipore) and stored at −70 °C. AAV vector titter, as viral genome per ml (vg/ml), was measured by quantitative real-time PCR as described ^13^. Known copy numbers (10^5^–10^8^) of the respective plasmid (pAAV-Tomato and pAAV-Lgi4) carrying the appropriate complementary DNA were used to construct standard curves.

Mice infection

5-6-weeks-old mice were anesthetized by intraperitoneal injection of 100 μl ketamine (60 mg/kg), xylazine (20 mg/kg) and atropine (9 mg/kg). Once asleep, animals were placed on a heated pad at 37 ± 0.5 ºC to prevent hypothermia. A ~ 4-mm incision was made in the skin to expose the right femoral vein. To increase vessel diameter and facilitate infusion, blood flow was interrupted with a cotton bud for a couple of seconds. Thereafter, 3.5 × 10^10^ virus particles were inoculated through the femoral vein in a final volume of 50 μl, taking care to prevent the introduction of air bubbles.

Based on previous work, 8 weeks after infection the protein is stably and specifically expressed in the cardiomyocytes ^7,10^.

Surface electrocardiographic (ECG) recording

From 8 weeks after infection, mice were anesthetized using isoflurane inhalation (0.8 ­ 1.0% volume in oxygen), and efficacy of the anaesthesia was monitored through breathing rate. Four-lead surface ECGs were recorded for 5 min, from subcutaneous 23-gauge needle electrodes attached to each limb and connected to an MP36R amplifier unit (BIOPAC Systems).

Lead-II was used to analyse the P, PR, QRS, QT and RR duration using AcqKnowledge 4.1 analysis software. QRS duration (before and after isoprenaline or carbachol) was measured as the time interval between the earliest moment of deviation from baseline and the moment when the S wave returned to the isoelectric line. QT duration was measured when the recording returned to the isoelectric line after T wave and was corrected by the Framingham equation ^14^.

Programmed Electrical Stimulation

From 8-weeks post-infection, mice were anesthetized by intraperitoneal injection of ketamine (60 mg/kg) and xylazine (20 mg/kg). Once anesthetized, the mouse was placed in a supine position and ECG electrodes were taped in its limbs. An octapolar catheter (Science) was inserted through the jugular vein and advanced into the right atrium (RA) and ventricle as previously described ^15^ and shown in Figure 12. Briefly, the right jugular vein was exposed and its proximal end was tied with a 6-0 suture. Another suture was placed at the distal end and it was tied once the catheter is properly placed. To insert the catheter, a small incision was made in the longitudinal direction of the vein. Proper catheter position was verified by visualization of the waveforms after stimulating the atria or the ventricle.

Atrial and ventricular arrhythmia inducibility was assessed by application of 10 atrial or ventricular bursts. These bursts consisted in 10 pulses of 5 ms duration each at 6 V at a frequency of 10 Hz followed by another 10 pulses at 33 Hz. Sinus node recovery time (SNRT), defined as the interval between the last stimulus in the pacing train and the onset of first spontaneous sinus beat, was analysed. PR duration was measured after each programmed electrical stimulation (PES) during the following 20 beats. Data were captured and analysed with LabScribe4, iWork.

Isolation of adult mice ventricular cardiomyocytes

Mouse ventricular cardiomyocytes (CMs) were isolated as previously described ^16,17^. From 8 weeks post infection, mice were euthanized in a CO_2_ chamber. The heart was quickly removed and placed in room temperature (RT) Ca^2+^-free perfusion buffer (PB, Table 2). Fat was removed and hearts were cannulated through the ascending aorta and mounted on a modified Langerdoff-perfusion apparatus. The heart was perfused with PB (1 ml/min) for 5 min at 37 ºC.

Subsequently, the tissue was enzymatically digested with digestion buffer (DB), which consisted in PB supplemented with 0.2 mg/ml Liberase^TM^, 5.5 mM trypsin 2.5% and 12.5 µM CaCl_2_ for 15 min at 37 ºC. At the end of the enzymatic digestion, atria tissue was discarded and both ventricles were isolated and physically disaggregated in 3 ml of DB. The resulting cell suspension was filtered through a 200 µm sterile mesh (SEFAR-Nitex) and enzymes were inactivated by adding up to 10 ml of stopping-buffer-1 (SB-1), which consists of PB supplemented with 7.5% v/v foetal bovine serum (FBS) and 12.5 µM CaCl_2_. After gravity sedimentation for 30 min, supernatant was removed and cardiomyocytes were gently resuspended and incubated for 20 min in stopping-buffer-2 (SB-2): PB containing 5% FBS and 12.5 µM CaCl_2_. Cardiomyocytes were reloaded with Ca^2+^ by incubation in SB-2 containing two progressively increased CaCl_2_ concentrations (0.112 and 1 mM). Cells were resuspended and allowed to settle for 15 min before decanting the supernatant between each step, contributing to the purification of the cardiomyocyte suspension. The rod-shaped cardiomyocytes homogenously resuspended in SB-2 with 1 mM CaCl_2_ were used during the same day of the isolation for electrophysiological experiments or fixed in PFA 4% for immunofluorescence assays.

# Table 2. Composition of the Ca^2+^-free perfusion buffer (PB) for the isolation of mouse ventricular cardiomyocytes.

| **Reagent** | **Final concentration (mM)** |
| --- | --- |
| NaCl | 113 |
| KCl | 4.7 |
| KH_2_PO_4_ | 0.6 |
| Na_2_HPO_4_ | 0.6 |
| MgSO_4_ • 7H_2_0 | 1.2 |
| NaHCO_3_ | 12 |
| KHCO_3_ | 10 |
| Phenol Red | 0.032 |
| HEPES | 9.9874 |
| Taurine | 30 |
| 2,3-Butanedione monoxime (BDM) | 10 |
| Glucose monohydrate | 5.5 |
| Dissolved in H_2_O and pH adjusted to 7.4 with NaOH | |

Electrophysiological recordings

For K_V_1.5 current recordings, HEK293 (or *Ltk^-^* cells for K_V_1.5/K_V_β2.1 currents) were plated in 60 mm culture dishes (Falcon) with 4 ml of complete DMEM. Cells were transfected at 60% confluence with the cDNA described in Table 3 using Lipofectamine® 2000 following manufacturer instructions. Lipofectamine 2000® Reagent was used 1:3 ratio (µg:µl) and a volume of transfection mix of 300 µl.

# Table 3. cDNA amount used for each transfection in patch-clamp experiments.

| **cDNA (µg)** | **K_V_1.5 currents** | **K_V_1.5/K_V_β1.3 currents** | **K_V_1.5/K_V_β1.2 currents** | **K_V_1.5/K_V_β2.1 currents** |
| --- | --- | --- | --- | --- |
| K_V_1.5 IRES GFP | 0.2 | - | 0.1 | - |
| K_V_1.5 IRES K_V_β1.3 | - | 2 | - | - |
| K_V_β1.2 | - | - | 2 | - |
| Empty, Lgi3 or Lgi4 IRES mCherry | 2 | 2 | 2 | 2 |

For K_V_4.3 and K_V_4.2 current recordings, CHO cells were plated in 60 mm culture dishes (Falcon) with 4 ml of complete Iscove. Cells were transfected at 60% confluence with 2 µg of K_V_4.3 pBK or K_V_4.2 GFP and 2 µg of empty IRES mCherry, Lgi3 IRES mCherry or Lgi4 IRES mCherry using Fugene® following manufacturer instructions. Fugene® Reagent was used in 1:3 (µg:µl) ratio and in a volume of transfection mix of 200 µl.

Solutions for electrophysiology experiments

The composition of the internal and external solutions used in HEK293, *Ltk^-^* and CHO cells are shown in Table 4.

# Table 4. Internal and external solutions used for patch-clamp recording in cell lines.

| Internal solution (mM) | | External solution (mM) | |
| --- | --- | --- | --- |
| K-Aspartate | 80 | NaCl | 145 |
| KCl | 42 | KCl | 4 |
| Phosphocreatine | 3 | CaCl_2_ | 1.8 |
| KH_2_PO_4_ | 10 | MgCl_2_ | 1 |
| Mg-ATP | 3 | HEPES | 10 |
| HEPES | 5 | Glucose | 10 |
| EGTA | 5 |  |  |
| pH adjusted to 7.25 with KOH | | pH adjusted to 7.4 with NaOH | |

To record K^+^ currents in isolated ventricular mouse cardiomyocytes, the solutions shown in Table 5 were used. The composition of the internal and external solutions used in isolated ventricular mouse cardiomyocytes cells (CMs) are shown in Table 5.

# Table 5. Internal and external solutions used for patch-clamp in mice isolated cardiomyocytes (CMs).

| Internal solution (mM) | | External solution (mM) | |
| --- | --- | --- | --- |
| K-Aspartate | 110 | NaCl | 136 |
| KCl | 20 | KCl | 5.4 |
| CaCl_2_ | 1 | CaCl_2_ | 0.9 |
| K_2_ATP | 4 | KH_2_PO_4_ | 1.2 |
| MgCl_2_ | 1 | HEPES | 5 |
| NaCl | 8 | Glucose | 10 |
| HEPES | 5 |  |  |
| EGTA | 10 |  |  |
| pH adjusted to 7.2 with KOH | | pH adjusted to 7.4 with NaOH | |

Ion currents and action potential acquisition, and data analysis

Before performing patch-clamp experiments in HEK293, *Ltk^-^* and CHO cells, fluorescent cells (mCherry and/or GFP positive cells) were isolated by using Fluorescence-Activated Cell Sorting (FACSVantage SE; BD Biosciences, Franklin Lakes, NK, USA) and preserved in complete DMEM or Iscove until being used. Cells aliquots were placed in the perfusion chamber on the stage of an inverted microscope (Eclipse TS-200, Nikon). The corresponding potassium currents elicited were recorded using the whole-cell configuration of the patch-clamp technique with an Axopatch-200B amplifier (Molecular Devices Co., USA) and these currents were low-pass filtered at 2 kHz and sampled at 4 kHz with an analogue to digital converter (Digidata 1322A, Molecular Devices). Experiments were performed at RT (22 ± 2 ºC).

Following cardiomyocyte’s isolation and Ca^2+^ re-introduction, cells were placed in a perfusion chamber (RC-26, Warner instruments) mounted on the stage of an inverted microscope (DMi8, Leica) and were allowed to settle on the bottom before being superfused with the external solution. Infected cells (expressing tdTomato) were selected with fluorescence. Whole-cell clamp recordings were made using an Axopatch-200B amplifier (Molecular Devices Co., USA). Voltage-clamp currents were low-pass filtered at 2 kHz and sampled at 4-10 kHz with an analogue to digital converter (Digidata 1550B, Molecular Devices, Foster City, USA). The experiments were performed at 32 ºC (TC324 temperature controller, Warner instruments).

In both cases (cell lines and CMs), pipettes were made from borosilicate glass capillaries (GD-1, Narishige, OD: 1 mm; ID 0.6 mm) with a programmable horizontal puller (P-87 or P-1000, Sutter Instruments) and polished with a microforge for cell lines recordings (MF-83, Narishige). The average pipette tip resistance, previously filled with the internal solution, ranged between 2.5 and 4 MΩ. GigaΩ seal formation was achieved by suction (2-5 GΩ). After seal formation, the whole-cell configuration was achieved by an additional suction. A series resistance compensation of 80-90% was reached. Data were recorded using pClamp 11.0 within clampex 11.0 and analysed with clampfit 11.1 (Molecular Devices, Foster City, USA). In addition, Origin 2020 (Origin-Lab Co) and clampfit 11.1 were used to perform least squares fitting as well as in data presentation. GraphPad Prism 9 was used for data presentation. In CMs, current amplitudes were normalized to the cell capacitance and expressed as current densities (pA/pF).

Experimental protocols

## HEK293 and CHO recordings

For K_V_1.5 currents, current-voltage (I-V) relationships were obtained after applying a 250 ms pulse in 10 mV increments from -80 to +60 mV from a -80 mV holding potential followed by a 250 ms pulse at -40 mV in which the closing of the channels can be measured at a frequency of 0.1 Hz. For K_V_4.3 currents, current-voltage (I-V) recordings were obtained following the application of a 250 ms pulse in 10 mV increments from -80 to +60 mV from a -80 mV holding potential preceded by a 250 ms pulse at -100 mV in order to prevent the run-down of the current ^18^ at a frequency of 0.1 Hz. I-V relationships represent the current amplitude at the end of the depolarizing pulse (K_V_1.5) or at the maximum peak (K_V_4.x) at each membrane potential tested.

Activation curves for K_V_1.5 channels were obtained by representing the normalized maximum amplitude of the tail current versus the previous depolarizing membrane potential applied. For K_V_4.x currents, the activation curve was obtained after representing the conductance of K_V_4.x channels (G_Kv4.x_) at each membrane potential. G_Kv4.x_ was calculated by the Ohm’s law modified for ion channels (G_Kv4.x_ = I_Kv4.x_/(E_m_ – E_rev_), where I_Kv4.x_ is the current at the maximum peak, E_m_ is the membrane potential applied and E_rev_ is the reversal potential (fixed at -85mV according to Chae et al., 2014 ^19^.

In both cases, a Boltzmann equation was fitted to obtain the voltage dependence of activation:

$$y=1/[1+\exp\left( -{(E_{m}-V_{1/2}}/s) \right)]$$

in which s represents the slope factor, E_m_ represents the membrane potential, and V_mid_ represents the voltage at which 50% of the channels are open.

Time constants of activation, inactivation and deactivation were obtained by fitting a single or double exponential function to the current traces, depending on its characteristics:

$$y=A_{s}\exp\left( -t/{\tau_{s}} \right)+A_{f}\exp\left( -t/{\tau_{f}} \right)+C$$

where τ_s_ and τ_f_ are the system time constants (slow and fast, respectively), A_s_ and A_f_ are the amplitudes of each component of the exponential, and C is the baseline value.

In order to study the effects of Lgi3-4 in the inactivation of K_V_1.5 in the presence of K_V_β1.x subunits, different approaches were carried out. Firstly, 250 ms depolarizing pulses at 140 mV were applied in order to measure whether K_V_β1.x subunits were still bound to K_V_1.5 channels in those cases in which the ß subunit-mediated inactivation properties were dimished. Secondly, to study the voltage-dependent effects on the N-type inactivation, a 10 ms pulse in 10 mV increments from -80 to +150 mV from a holding potential of -80 mV followed by a 250 ms at 50 mV. A Boltzmann equation was fitted to obtain the voltage dependence of inactivation, as previously described ^20^.

## Mouse ventricular isolated cardiomyocytes recordings

For action potential (AP) recordings, the threshold current was determined using 1-ms pulses at increasing amplitudes (0.2 nA/pulse) and at a frequency of 1 Hz. Thereafter, action potentials (APs) were evoked by the injection of 1-ms pulses of constant amplitude at frequencies ranging from 1 to 10 Hz. AP duration (APD) was measured at 20, 50, 70, and 90% repolarization of the AP.

For the current-voltage (I-V) recordings different approaches were carried out to separate the different K^+^ currents, as previously reported ^17^. Total K^+^ currents in mice cardiomyocytes are composed of the transient outward K^+^ current (*I*_to_), the rapidly activating and slowly inactivating outward K^+^ current (*I*_Kur_), the slowly activating and slowly inactivating steady-state K^+^ current (*I*_ss_), and the inwardly rectifying K^+^ current (*I*_K1_). I-V relationships of the outward and inward potassium currents were constructed from the current changes produced by a 500 ms voltage-clamp step applied in 10 mV increments from -140 to +50 mV from a -80 mV holding potential. The isolation of each of these currents is well-stablished ^17,21,22^ and it was carried out as follows: *I*_to_ was measured as the difference of the current obtained by subtracting records with an inactivating pre-pulse voltage-clamp protocol (100 ms at -20 mV) from those without the inactivating pre-pulse (Figure S5A). *I*_Kur_ was calculated by subtracting the currents recorded with the inactivating pre-pulse in the presence of 200 μM 4-aminopyridine (4-AP) from those recorded in its absence (Figure S8B). Thus, the remaining 4-AP-resistant current corresponds to *I*_ss_ (Figure S8C). All these outward K^+^ currents were measured at the maximum peak current. For inward currents, 500 ms voltage-clamp step were applied in 10 mV increments from -140 to +20 mV from a -80 mV holding potential. These currents were measured at the end of the 500 ms pulses. Current recordings were all obtained at 1 Hz.

Protein extraction

HEK293 cells at 80% confluence in 100 mm culture dishes (353003 Corning, Corning, NY, USA) were transfected with 4 μg of each cDNA in different combinations, by using Lipofectamine 2000 following the manufacturer instructions in a final volume of 1400 μl. After 24 h, cells were washed twice with cold PBS and scrapped in 0.5 ml of Protein Lysis Buffer 1 (150 mM NaCl, 50 mM HEPES, 10% glycerol (G5516, Sigma Aldrich), 1% Triton X-100 (T8787, Sigma Aldrich), pH=7.2 with NaOH) supplemented with protease inhibitors (0.1% v/v Protease Inhibitor Cocktail [PIC, P8340, Sigma Aldrich], 1 mM PMSF). The homogenates were incubated in orbital agitation at 4 ºC 30 min and then centrifuged at 13000 × g for 15 min. The supernatants were transferred to new 1.5 ml tubes and the total protein content of each supernatant was determined using a BCA Pierce Kit.

For native cardiac tissue, it was preserved at -80 ºC until being homogenized. After defrosting the tissue, it was chopped with PBS and tubed in a 2 ml eppendorf with 300 μl of Lysis Buffer 2 (50 mM Tris-HCl, 320 mM sucrose (1076541000, Sigma Aldrich), 0.2% v/v Igepal (56741, Sigma Aldrich), 1 mM DTT (43819, Sigma Aldrich), supplemented with 0.1% v/v, Phosphatase Inhibitor Cocktail 2 (P4726, Sigma Aldrich), 0.1% v/v Phosphatase Inhibitor Cocktail 3 (P0044, Sigma Aldrich), 0.1% v/v PIC and 1% (v/v) Trypsin Inhibitor (10109886001, Roche) at pH 8.0). Samples were then mechanically homogenized with a Polytron (ULTRA-TURRAX^®^ T10 Basic Disperser, IKA^®^ Works) while maintained in ice to avoid sample heating. Once homogenized, samples were centrifuged at 13000 rpm 15 min at 4 ºC and supernatants were transferred to clean 1.5 ml tubes. Lastly, samples were vortexed for 45 min at 4 ºC. Finally, protein content was determined by using BCA Pierce Kit (Pierce® BCA Protein Assay, 23227, ThermoFisher Scientific).

Western-blot

Samples were resuspended in Laemmli buffer with 5% β-mercaptoethanol and boiled for 5 min at 95 ºC. Then, they were centrifuged for 3 min at 5000 × g and 30 μl of protein extract was separated by SDS-PAGE (8% acrylamide/bisacrylamide) gels. The proteins were transferred to PVDF membranes and blocked with BSA 5% in TBST during 2 h. After washing the membranes three times with TBST, 10 min each, they were incubated with the corresponding primary antibody (Supplementary Table 6) diluted in TBST overnight at 4 ºC. Then, the membranes were washed three times and incubated with the secondary antibody conjugated with peroxidase (anti-mouse IgG-HRP, sc-516102, Santa Cruz; anti-rabbit IgG-HRP, A130-108P, Bethyl) 1:10000 to 1:20000 diluted in TBST 2.5% BSA. Secondary antibodies were revealed by using Clarity Western ECL Substrate (1705061, Bio-Rad).

Coimmunoprecipitation assays

Immunoprecipitation (IP) was performed with Protein A (for rabbit antibodies ) or G (for mouse ones). Briefly, Protein A or G Sepharose® beads were incubated with anti-K_V_1.5 antibodies (APC-004, rabbit polyclonal, Alomone), anti-FLAG antibodies (F7425, rabbit polyclonal, Sigma) or anti K_V_β1.2 antibodies (K47/42, Neuromab) in a concentration of 4 ng antibody/μg protein for the positive IP, and without antibodies for the negative IP, for 2h at 4 ºC. In other tubes, 400-1000 µg of total protein lysate were incubated with Protein A/G 2h at 4 ºC, and then centrifuged at 5000 × g 30’’ to remove those protein A/G unspecific binding. Afterwards, the supernatant containing the protein lysate was incubated with Protein A/G with or without antibody overnight at 4 ºC, for the positive and negative IP, respectively. The samples were centrifugated at 5000 × g 30’’ and the supernatant was prepared to be loaded in the western-blot, whereas the precipitate was washed twice with washing buffer (150 mM NaCl, 50 mM HEPES, 10% glycerol (G5516, Sigma Aldrich), 0.1% Triton X-100 (T8787, Sigma Aldrich), pH=7.4) and the proteins are eluted with Laemmli buffer with 2% β-mercaptoethanol and boiled for 5 min at 95 ºC.

Immunofluorescence

COS7 cells: they were plated in coverslips with DMEM supplemented with 10% FBS and 1% penicillin-streptomycin (PS). At ~40% confluence, cells were transfected with 0.5 μg of each DNA with Lipofectamine® 2000 following manufacturer’s instructions. 48 h post-transfection, cells were washed with PBS and fixed with PFA 4% 15 min at RT. After three washes with PBS, cells were permeabilized with 0.2% Triton X-100 (T8787, Sigma-Aldrich) in PBS 5 min and washed again three times. To avoid nonspecific unions, cells were incubated with blocking buffer (10% goat serum (GS) in PBS, 16210-064, Gibco) 15 min at RT. The coverslips were washed three times with PBS and incubated with the corresponding primary antibodies diluted in blocking buffer 1 h at 37 ºC in a wet chamber. After three washes with PBS, cells were incubated with the corresponding secondary antibodies diluted at 1:500 in blocking buffer for 1 h at 37 ºC. Cells were washed and nucleus were stained with DAPI (D1306, Thermo Fisher Scientific, 1:1000 in blocking buffer) for 10 min at RT. The coverslips were washed with PBS and mounted with Prolong Diamond Antifade Reagent (P36970, Molecular Probes), observed under confocal microscopy (LSM710, Zeiss) and processed with FIJI and ImageJ programs.

Isolated mice cardiomyocytes: previously fixed cells were washed in PBS and centrifuged at 100 × g for 4 min at RT. From now on, all the centrifugations were carried out at 100 × g during 4 min at RT. 15 μl of the sample were added to a well in a conical V bottom polystyrene 96-well non-treated plates (277143, Nunc, Thermo Fisher Scientific) and washed with PBS. Then, were blocked in Triton-X-100 0.3%, BSA 5% in PBS 90 min at RT. In those cases in which the plasmalemma needs to be intact the same procedure was used but lacking Triton-X-100. Cells were incubated with the corresponding primary antibodies in BSA 5%, GS 2% with or without Triton-X-100 0.3% in PBS overnight at 4 ºC. Cardiomyocytes were three times washed with PBS prior to its incubation with the corresponding secondary antibodies diluted in BSA 5%, GS 2% with or without Triton-X-100 0.3% in PBS 60 min at RT. Cells were washed with PBS before its incubation with DAPI (1:1000) in Triton-X-100 0.3%, BSA 5%, GS 2% in PBS during 10 min at RT. Cells were washed again and resuspended in 15 μl of PBS, the drop was placed in a microscope slide and, when dried, mounted (Prolong Diamond Antifade Reagent, P36970, Molecular Probes) and covered with a coverslip. Labelled cells were observed under confocal microscopy (LSM710, Zeiss) and processed with FIJI and ImageJ programs as follows:

- To measure membrane K_V_1.5 expression in non-permeabilized cells, different values (area, mean intensity and integrated density) of positive signal were measured from previously thresholded cells.
- To analyse the colocalization, we measured the Pearson coefficiens by using the JaCoP plugin of FIJI.

Human hearts: 5 μm sections of paraffin embedded cardiac tissue mounted on slides coated with 3-aminopropyltriethoxysilane (Sigma) were used. The tissue was dewaxed at 60 ºC during 30 min and washed with xylol (twice, 5 min). Then, the tissue was rehydrated by using decreasing alcohol concentrations (100% - 96% - 70% - distilled water, 5 min in each). Afterwards, the tissue was incubated with citrate buffer (citric acid 10 mM, sodium citrate 10 mM, pH 6.0), microwaved at maximum heat for 3 min and rest for 15 min. Then, it was washed with PBS during 5 min and blocked with GS 5% (16210-064, Gibco). Primary antibodies were diluted in 1% BSA PBS (see Table 6) and incubated overnight at 4 ºC in a wet chamber. After washing with PBS (5 min), the tissue was incubated with the corresponding secondary antibody (goat anti-Rabbit IgG Alexa Fluor 488, A11034, Molecular Probes) diluted at 1:100 in 1% BSA PBS during 25 min at RT. The tissue was washed with PBS (5 min) and dried. Then, the sample was incubated with DAPI 1:500 in PBS for 5 min, washed with PBS and mounted with Prolong Diamond Antifade Mounting (P36970, Molecular Probes). Labelled cells were observed under confocal microscopy (LSM710, Zeiss) and processed with FIJI and Imaris software programs. Z-stacks were taken every 1 μm and maximal projections of images were reconstructed using Imaris software (Bitplane Scientific Software) using 3 z planes. At least 4 regions were quantified per porta using Imaris-Integrated Optical Density. Mean Gray Value (intensity mean) was used to determine the fluorescence intensity.

Atrial human tissue tissue was fixed in 4% PFA 1h at RT and washed three times with PBS. Then, a sucrose gradient was performed as follows: sucrose 10% in PBS 1h RT, sucrose 20% in PBS 3h in cold agitation and sucrose 30% in PBS 3h in cold agitation. During the next day, samples were frozen in OCT. 6 μm sections of OCT-embedded tissue were sliced. The tissue was washed with PBS and incubated with 50 mM NH_4_Cl during 10 min to decrease the background fluorescence. Then, it was permeabilized with 0.2% Triton X-100 in PBS 10 min and blocked with 5% BSA in PBS during 30 min. Tissue was incubated with the primary antibodies at its corresponding concentration in 1% BSA overnight at 4 ºC, washed with PBS and incubated with secondary goat anti rabbit AF488 antibody (1:500 in PBS 1% BSA, Invitrogen) during 1h at RT. Then, To-pro (T3605, Invitrogen) was added to dye the nuclei (1:500 in PBS). Samples were washed in PBS and in distilled water, and mounted in aqueous medium (50% PBS, 50% glycerol). Labelled cells were observed under confocal microscopy (Leica SP5, Leica) and processed with FIJI and ImageJ programs. Pearson correlation coefficient was calculated with Leica ASF afterselecting individual cells in each image.

# Table 6. Primary antibodies used for western blot and immunofluorescence.

| Antibody | Type and host | Dilution WB | Dilution IF | Reference and origin |
| --- | --- | --- | --- | --- |
| Anti-K_V_1.5 | Polyclonal Rabbit | 1:1000 | 1:200 | APC004, Alomone |
| Anti-K_V_1.5_ext_ | Polyclonal Rabbit | - | 1:50 | APC150, Alomone |
| Anti-K_V_4.3 | Polyclonal Rabbit | - | 1:200 | APC017, Alomone |
| Anti-Lgi3 | Polyclonal Rabbit | 1:1000 | 1:200 | ab113950, Abcam |
| Anti-Lgi3 | Polyclonal Rabbit | 1:500 | 1:100 | 21919-1-AP, Proteintech |
| Anti-Lgi4 | Polyclonal Rabbit | ~~-~~ | 1:50 | HPA043556, Sigma |
| Anti-Lgi4 | Monoclonal Rat | 1:300 | - | 011311H12, Absea Biotechnology |
| Anti-Lgi1 | Polyclonal Goat | - | 1:100 | Sc9581, Santa Cruz Biotech |
| Anti-Lgi2 | Polyclonal Rabbit | - | 1:100 | HPA017140, Sigma |
| Anti-FLAG | Polyclonal Rabbit | 1 μg/ml | 1:1000 | F7425, Sigma |
| Anti-FLAG | Monoclonal Mouse | 1 μg/ml | 1:1000 | F3165, Sigma |
| Anti-GFP | Monoclonal Mouse | 1:400 | - | 118144600001, Roche |
| Anti-K_V_β1.2 | Monoclonal Mouse | 1:500 | - | K47/42, Neuromab |
| Anti-Vinculin | Monoclonal Mouse | 1:80000 | - | Sc73614, Santa Cruz Biotech |
| Anti-Rab8A | Polyclonal Rabbit | - | 1:200 | 55296-1-AP, Proteintech |
| Anti-Rab5 | Monoclonal Mouse | - | 1:100 | Sc-46692, Santa Cruz Biotech |
| Anti-Rab11A | Monoclonal Mouse | - | 1:100 | Sc-166912, Santa Cruz Biotech |

Membrane protein expression with Flow Cytometry

HEK293 cells were plated in 6-well multiwell cell plates were transfected at 80% confluence with K_V_1.5-HA (0.2 μg) + IRESmCherry/Lgi3-IRESmCherry or Lgi4-IRESmCherry (2 μg) + K_V_β1.3/ K_V_β1.2/K_V_β2.1 or empty pBK (2 μg) using Lipofectamine 2000 following manufacturer’s instructions.

48h post-transfection, cells were washed with PBS without K^+^ and briefly detached from the bottom (TrypLE^TM^ Express). To stop the enzyme digestion, cold supplemented DMEM was added and placed on ice to prevent the internalization of surface agents. Tubes were centrifuged at 400 × g for 5 min at 4 ºC, the supernatant was discarded and the cells were resuspended in PBS without K^+^ by vortex. This wash step was repeated twice and1 × 10^6^ cells were used to the cell surface staining. Anti-HA (1:500, Santa Cruz Biotechnology) was added and cells were vortexed and incubated for 45 min at 4 ºC in orbital agitation. PBS without K^+^ were added before centrifuging at 400 × g for 5 min at 4 °C, the supernatant was discarded and the cells were twice resuspended in PBS without K^+^. to remove any unbound antibody and, after the last centrifugation, cells were resuspended in PBS without K^+^. Then, the secondary antibody, goat anti-mouse AF488 (1:500) was added, cells were vortexed and incubated for 45 min at 4 ºC in orbital agitation. PBS without K^+^ were added before centrifuging at 400 × g for 5 min at 4 °C, the supernatant was discarded and the cells were twice resuspended in PBS without K^+^. Finally, cells were resuspended in PBS without K^+^ and transferred to flow cytometry tubes and kept in the dark at 4 ºC until running the sample.

Cells were analysed by using FACSCANTO II. Right before running each sample, DAPI (1:20000) was added to the cell suspension in order to select only the intact cells (with no DAPI staining). Cell debris and cell aggregates were also excluded. AF488 signal was measured in mCherry positive and DAPI negative cells in at least 20000 events per sample.

RNA determination

Total RNA was extracted from human atrial tissue by using the kit Maxwll 16. Reverse transcription of RNA was performed with AffinityScript Multiple Temperature cDNA Synthesis Kit (600107, Agilent) and Taqman Real Time PCR (Applied Biosystems) was performed on a StepOnePlus Real Time qPCR system (Applied Biosystems) and data were processed with the namesake program. All procedures were conducted following manufacturer’s instructions.

# Table 7. List of TaqMan used to determine mRNA expression in human heart.

| Protein | Gene | Reference |
| --- | --- | --- |
| K_V_1.5 | *KCNA5* | Hs00969279_s1 |
| Lgi3 | *LGI3* | Hs00373763_m1 |
| Lgi4 | *LGI4* | Hs00373801_m1 |
| GAPDH | *GAPDH* | H02758991_g1 |

Statistical analysis

Data are expressed as mean ± SEM where N represents the number of patients or animals, and n represents the number of individual cells. Comparisons were performed between different experimental groups by an unpaired two-tailed Student’s t-test or with multiple comparison Mann-Whitney’s test. When more than two experimental groups were compared, one-way or two-way ANOVA with Tukey’s or Šídák’s multiple comparison test, respectively, were used. Contingency analysis was performed with chi-square test. Differences were considered significant when p<0.05.

SUPPLEMENTARY REFERENCES

1. Uebele, V. N., England, S. K., Chaudhary, A., Tamkun, M. M. & Snyders, D. J. Functional Differences in Kv1.5 Currents Expressed in Mammalian Cell Lines Are Due to the Presence of Endogenous Kvβ2.1 Subunits. *J. Biol. Chem.* **271**, 2406–2412 (1996).

2. de Benito-Bueno, Á. Characterization of the human cardiac KV1.5 channelosome: role of KChIP2. (Universidad Autónoma de Madrid, 2022).

3. Kwak, Y.-G., Navarro-Polanco, R. A., Grobaski, T., Gallagher, D. J. & Tamkun, M. M. Phosphorylation Is Required for Alteration of Kv1.5 K+ Channel Function by the Kvβ1.3 Subunit. *J. Biol. Chem.* **274**, 25355–25361 (1999b).

4. David, M. *et al.* Protein Kinase C (PKC) Activity Regulates Functional Effects of Kvβ1.3 Subunit on KV1.5 Channels. *J. Biol. Chem.* **287**, 21416–21428 (2012).

5. Franqueza, L. Functional expression of an inactivating potassium channel (Kv4.3) in a mammalian cell line. *Cardiovasc. Res.* **41**, 212–219 (1999).

6. Zadeh, A. D. *et al.* Internalized Kv1.5 traffics via Rab-dependent pathways: Rab-dependent trafficking of Kv1.5. *J. Physiol.* **586**, 4793–4813 (2008).

7. Macías, Á. *et al.* Kir2.1 dysfunction at the sarcolemma and the sarcoplasmic reticulum causes arrhythmias in a mouse model of Andersen–Tawil syndrome type 1. *Nat. Cardiovasc. Res.* **1**, 900–917 (2022).

8. Prasad, K.-M. R., Xu, Y., Yang, Z., Acton, S. T. & French, B. A. Robust cardiomyocyte-specific gene expression following systemic injection of AAV: in vivo gene delivery follows a Poisson distribution. *Gene Ther.* **18**, 43–52 (2011).

9. Bezzerides, V. J. *et al.* Gene Therapy for Catecholaminergic Polymorphic Ventricular Tachycardia by Inhibition of Ca ^2+^ /Calmodulin-Dependent Kinase II. *Circulation* **140**, 405–419 (2019).

10. Cruz, F. M. *et al.* Exercise Triggers ARVC Phenotype in Mice Expressing a Disease-Causing Mutated Version of Human Plakophilin-2. *J. Am. Coll. Cardiol.* **65**, 1438–1450 (2015).

11. Xiao, X., Li, J. & Samulski, R. J. Production of High-Titer Recombinant Adeno-Associated Virus Vectors in the Absence of Helper Adenovirus. *J. Virol.* **72**, 2224–2232 (1998).

12. Hauswirth, W. W., Lewin, A. S., Zolotukhin, S. & Muzyczka, N. [48] Production and purification of recombinant adeno-associated virus. in *Methods in Enzymology* vol. 316 743–761 (Elsevier, 2000).

13. Prasad, K.-M. R. *et al.* Topoisomerase Inhibition Accelerates Gene Expression after Adeno-associated Virus-mediated Gene Transfer to the Mammalian Heart. *Mol. Ther.* **15**, 764–771 (2007).

14. Sagie, A., Larson, M. G., Goldberg, R. J., Bengtson, J. R. & Levy, D. An improved method for adjusting the QT interval for heart rate (the Framingham Heart Study). *Am. J. Cardiol.* **70**, 797–801 (1992).

15. Li, N. & Wehrens, X. H. T. Programmed Electrical Stimulation in Mice. *J. Vis. Exp.* 1730 (2010) doi:10.3791/1730.

16. García-Prieto, J. *et al.* β3 adrenergic receptor selective stimulation during ischemia/reperfusion improves cardiac function in translational models through inhibition of mPTP opening in cardiomyocytes. *Basic Res. Cardiol.* **109**, 422 (2014).

17. Macías, Á. *et al.* Paclitaxel mitigates structural alterations and cardiac conduction system defects in a mouse model of Hutchinson–Gilford progeria syndrome. *Cardiovasc. Res.* **118**, 503–516 (2022).

18. Hattori, S., Murakami, F. & Song, W.-J. Rundown of a transient potassium current is attributable to changes in channel voltage dependence. *Synapse* **48**, 57–65 (2003).

19. Chae, Y. J., Choi, B. H., Choi, J.-S. & Hahn, S. J. Block of Kv4.3 potassium channel by trifluoperazine independent of CaMKII. *Neurosci. Lett.* **578**, 159–164 (2014).

20. Macías, A. *et al.* PKC inhibition results in a K _v_ 1.5 + K _v_ β1.3 pharmacology closer to K _v_ 1.5 channels: K _v_ 1.5 + K _v_ β1.3 pharmacology and PKC. *Br. J. Pharmacol.* **171**, 4914–4926 (2014).

21. Fiset, C., Clark, R. B., Larsen, T. S. & Giles, W. R. A rapidly activating sustained K ^+^ current modulates repolarization and excitation-contraction coupling in adult mouse ventricle. *J. Physiol.* **504**, 557–563 (1997).

22. Trépanier-Boulay, V., St-Michel, C., Tremblay, A. & Fiset, C. Gender-Based Differences in Cardiac Repolarization in Mouse Ventricle. *Circ. Res.* **89**, 437–444 (2001).

SUPPLEMENTARY FIGURES


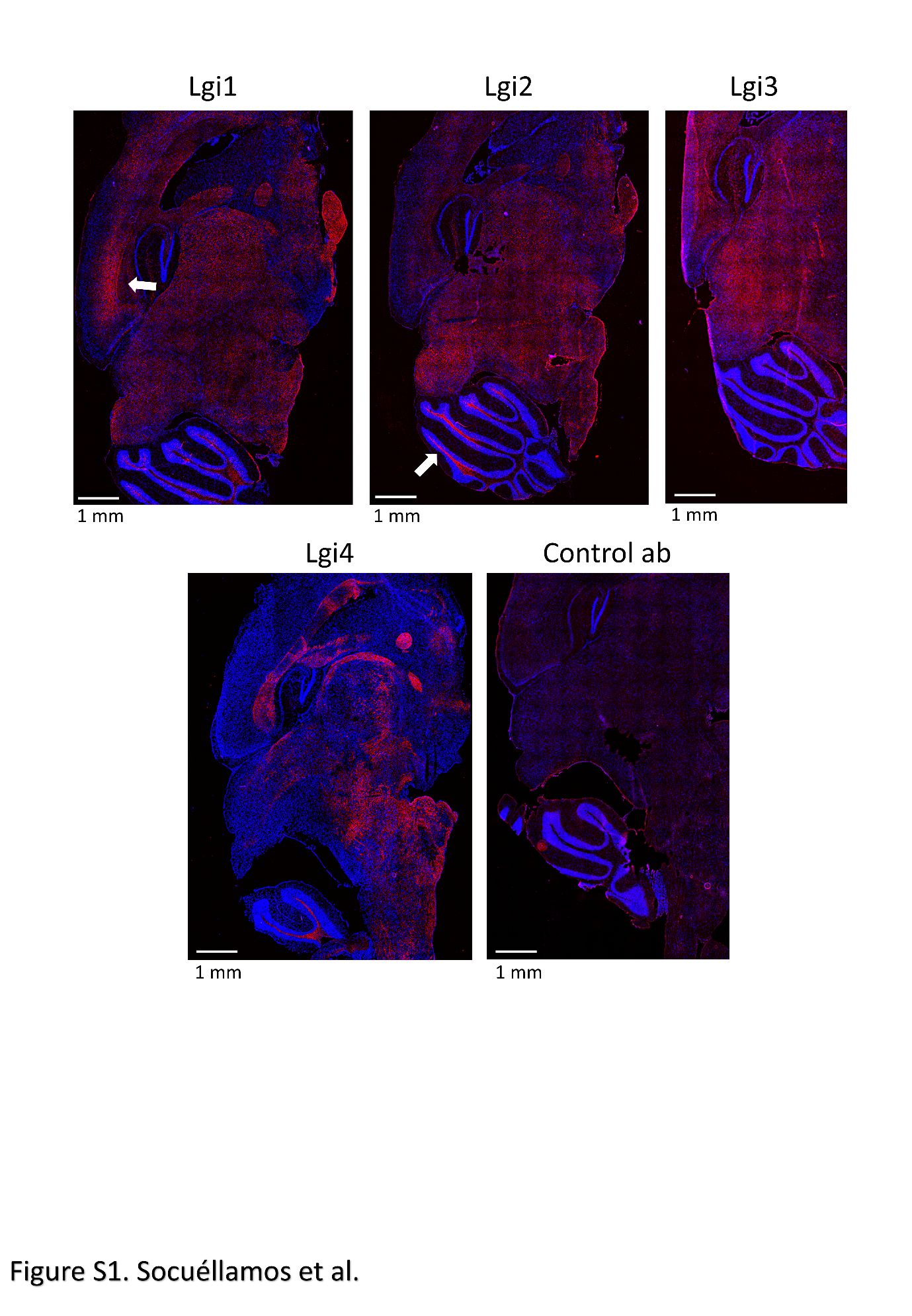


**Figure S1. Positive control for Lgi1-4 antibodies.** Representative images of the immunodetection of Lgi1-4 in brain slices from mice, demonstrating that Lgi1-4 antibodies are specific, as shown by their distinct expression patterns. Scale bar: 1 mm.


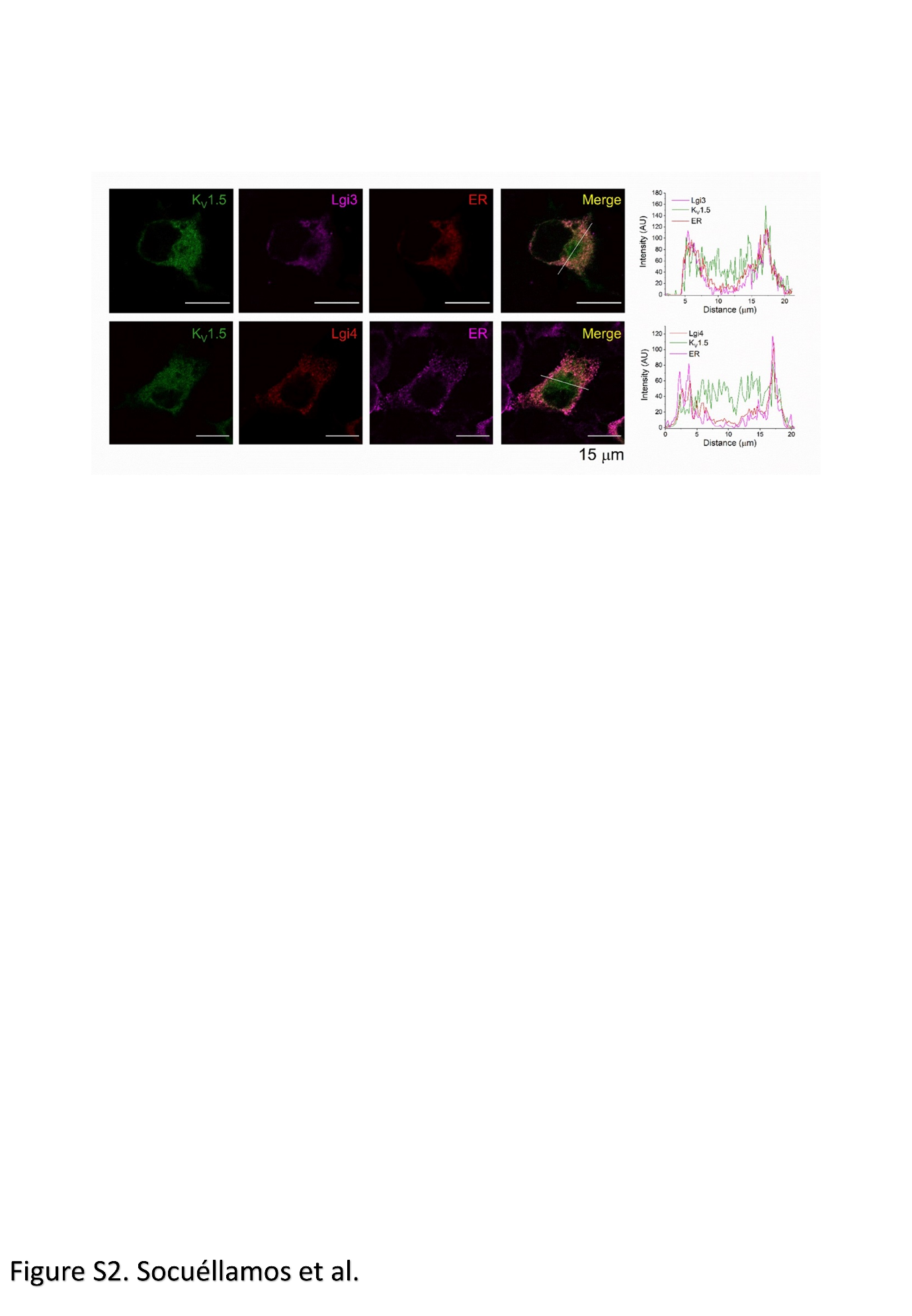
**Figure S2. Interaction of Lgi3-4 with K_V_1.5 in the ER in heterologous systems.** Representative confocal images of COS-7 cotransfected with K_V_1.5 and Lgi3 (upper panels) or Lgi4 (lower panels) and the ER marker DsRed. Scale bar: 15 µm.


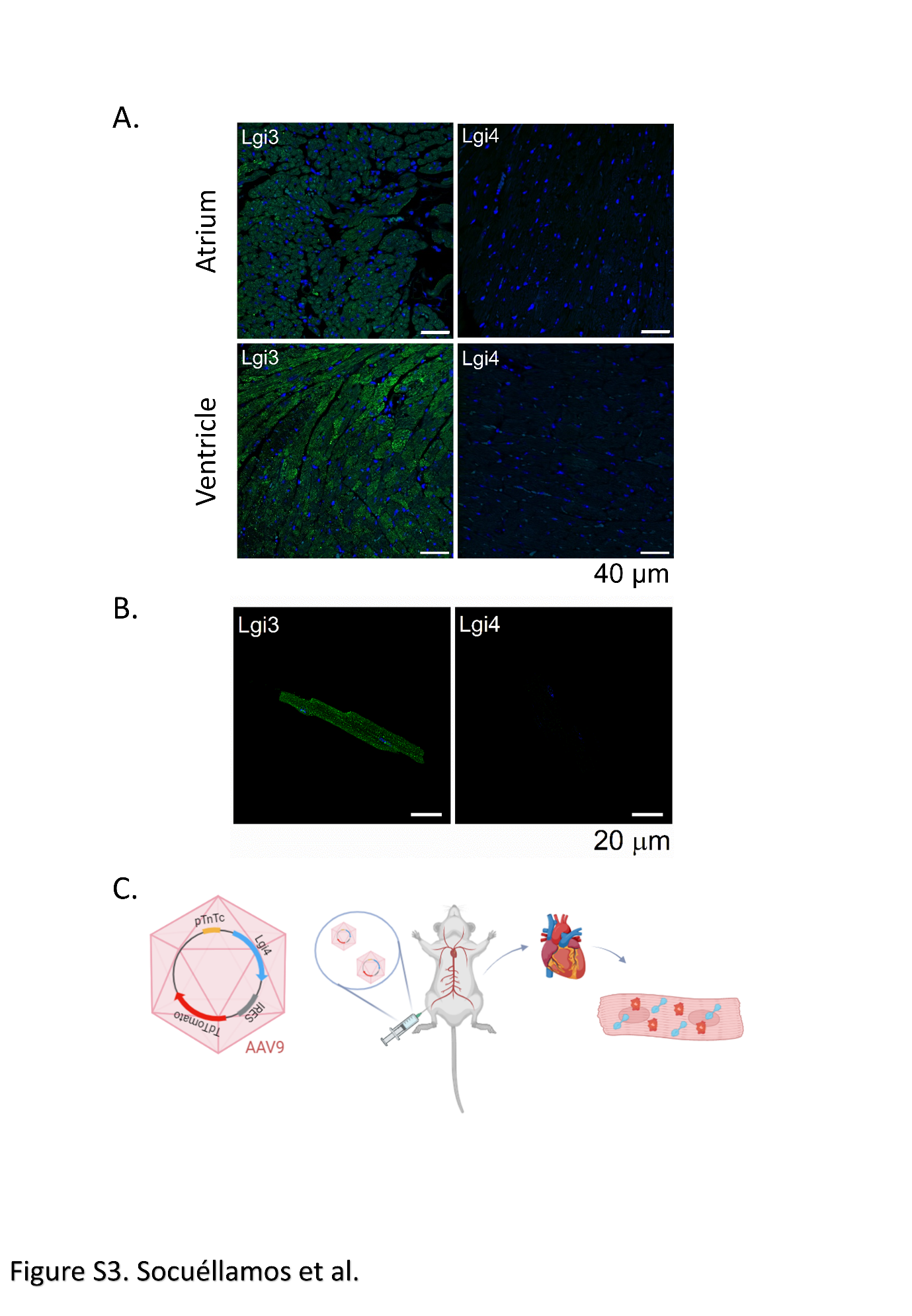
**Figure S3. Lgi4 is not expressed in mice atria or ventricle.** **A)** Representative confocal images of Lgi3 and Lgi4 immunostaining in atria and ventricle. Scale bar: 40 µm. **B)** Representative images of the immunodetection of Lgi3 and Lgi4 in permeabilized non-infected mice ventricular CMs. Note that Lgi4 is not expressed in atria nor ventricle (N=3, n=30). Scale bar: 20 µm. **C)** Schematic representation of the generation of the mice model expressing Lgi4 in cardiac myocytes. AAV9 carrying Lgi4 or empty AAV-vector were introduced through the femoral vein of the mice, being transduced only in cardiomyocytes due to its cTnT promoter. Scheme created with BioRender.


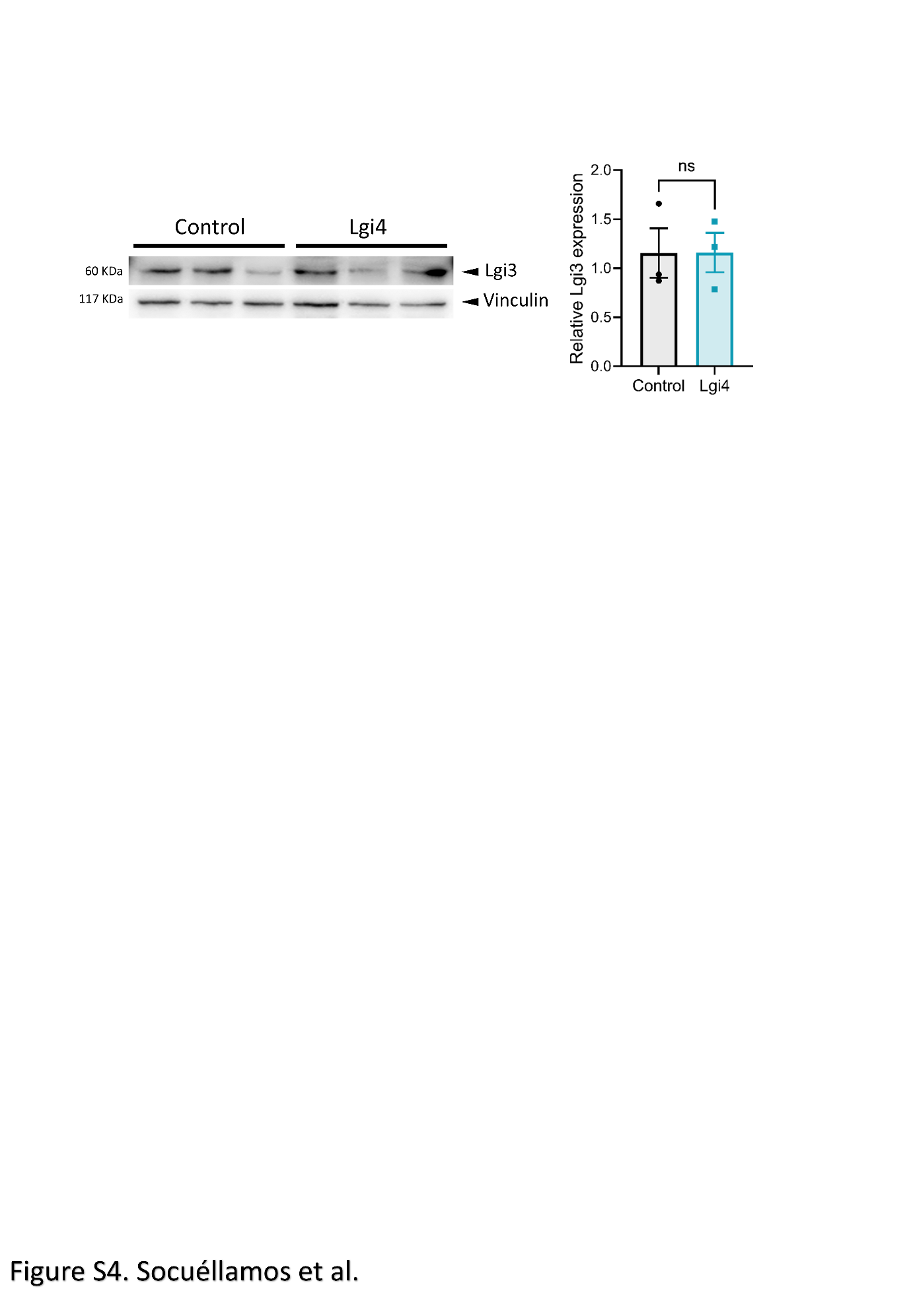
**Figure S4. Lgi3 expression is not altered in cardiomyocytes from Lgi4-cardiac expressing mice.** Representative immunoblots of anti-Lgi3 and anti-Vinculin in ventricular tissue from Control and Lgi4-cardiac expressing mice. Quantification of the relative protein expression of Lgi3 normalized by vinculin expression is shown at the right.


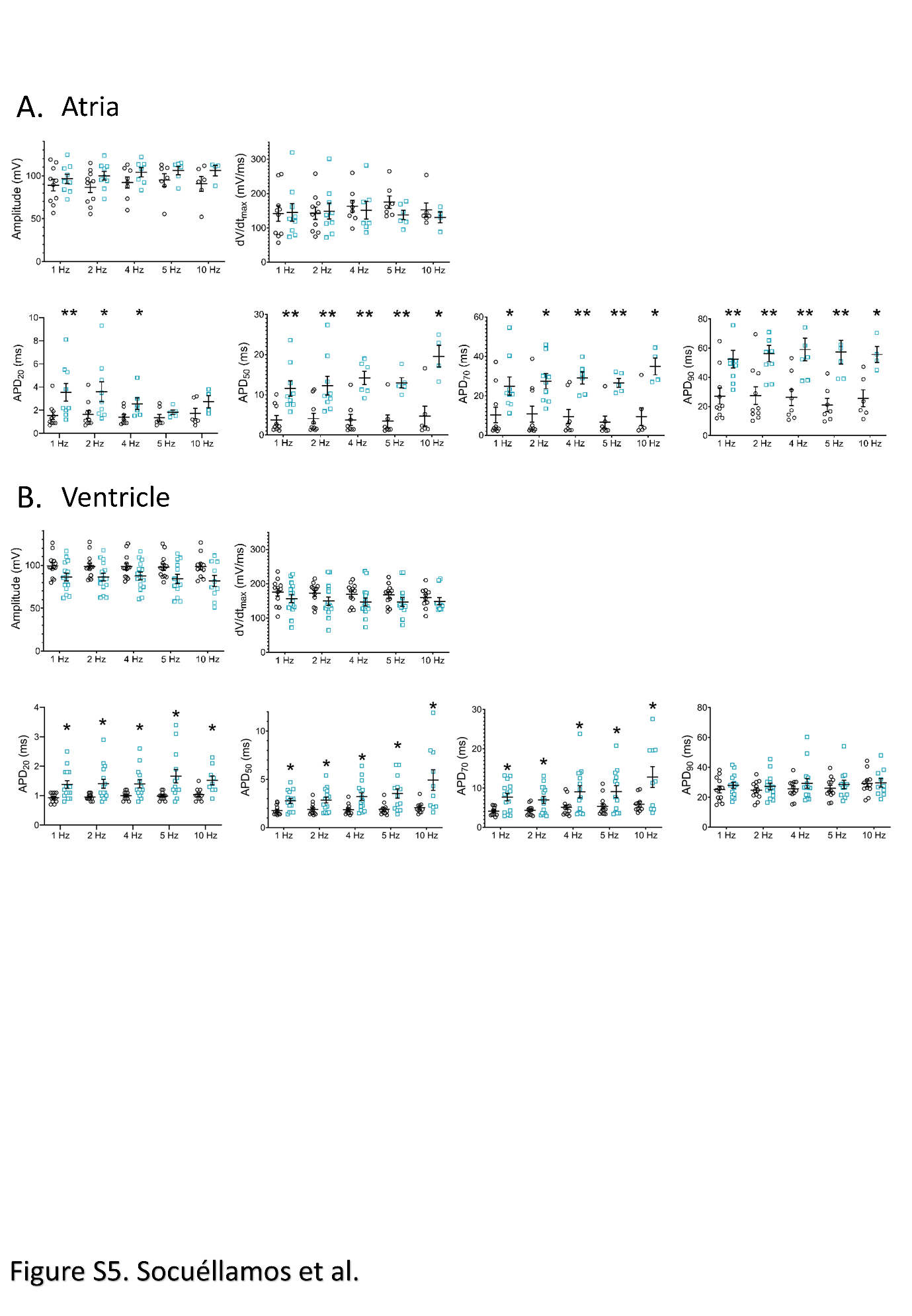
**Figure S5. Electrophysiological parameters of the cardiac AP in atrial and ventricular cardiomyocytes stimulated at 1-10 Hz.** In the upper graphs, the maximal amplitude of the AP (Amplitude) and the maximum upstroke velocity (dV/dt_max_) are represented at different frequencies of stimulation in atrial (**A**) and ventricular (**B**) cardiomyocytes. In the lower graphs, APD measured at the 20, 50, 70 and 90% of repolarization is represented in CMs from the atria (**A**) and ventricle (**B**) stimulated at frequencies from 1 to 10 Hz. Note that APD_20_ to APD_70_ are longer in ventricular CMs from Lgi4 mice than in Control ones, especially at higher stimulating frequencies. In atrial CMs from Lgi4-mice, the AP is dramatically prolonged at all the stimulating frequencies.


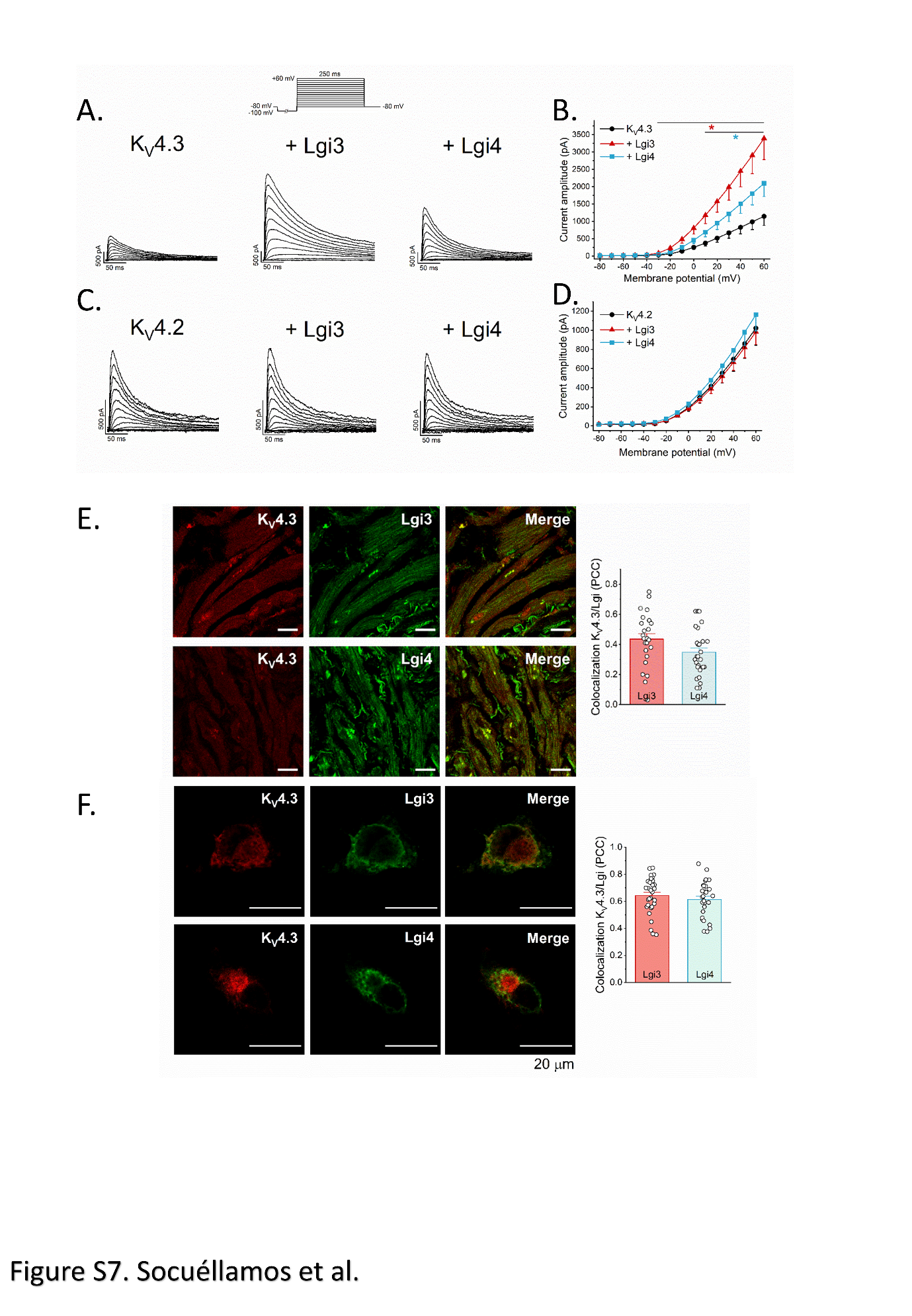
**Figure S6. Lgi3-4 modulate the current amplitude of K_V_4.3 but not that of K_V_4.2.** The upper panel shows the I-V protocol used to record K_V_4 currents **A)** Original current recordings of K_V_4.3 in the absence and in the presence of Lgi3-4 in CHO cells. Scale bar: 50 ms in the *x* axis and 500 pA in the *y* axis. **B)** Current-voltage relationship measured at the peak of the 250 ms depolarizing-pulse (n=18-24). **C)** Original current traces of K_V_4.2 in the absence and in the presence of Lgi3-4 in CHO cells. Scale bar: 50 ms in the *x* axis and 500 pA in the *y* axis. **D)** I-V measured at the maximum peak of the depolarizing-pulses (n=12-16). Data represent mean±SEM. **E)** Representative confocal images of the immunodetection of K_V_4.3 with Lgi3 (upper panels) and Lgi4 (lower panels) in human atrium slices from patients in SR (N=5-6, n=25-30). Scale bar: 20 µm. PCC between either Lgi3-4 and K_V_4.3 are shown on the right graph. Values represent mean±SEM. **F)** Representative confocal images of COS7 cells cotransfected with K_V_4.3 and Lgi3 (upper panels) or Lgi4 (lower panels). Scale bar: 20 µm. Quantification of the colocalization between K_V_4.3 and Lgi3 or Lgi4 (n=28-30). Data represent mean±SEM of the indicated n.


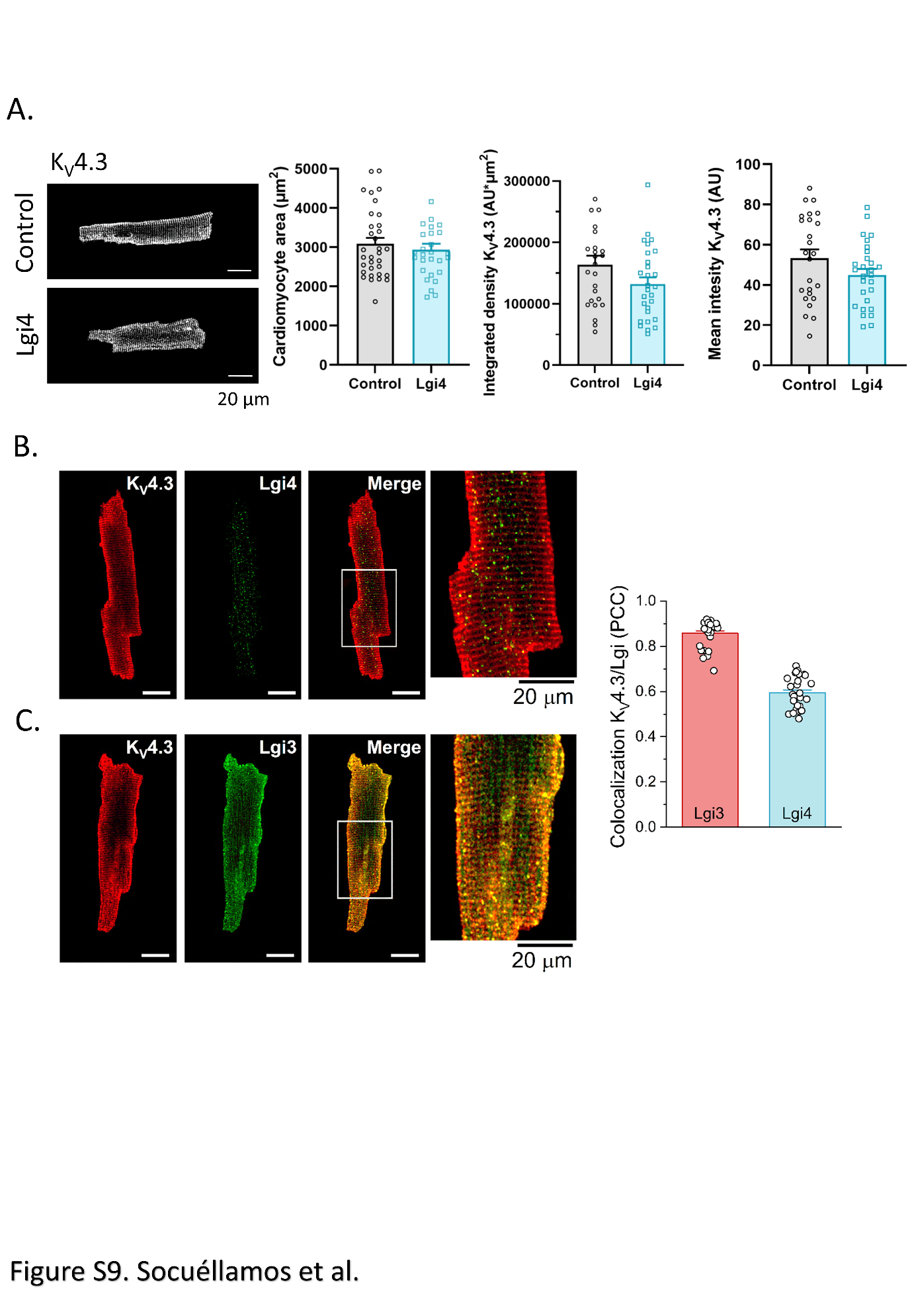
**Figure S7. Lgi4-3 colocalize with K_V_4.3 but Lgi4 does not alter its expression. A)** Left panel shows representative confocal images of the immunodetection of K_V_4.3 in permeabilized mice ventricular Control (up) and Lgi4 (down) CMs. Scale bar: 20 µm. Bar graphs show the quantification of the CMs area (left), the integrated density of K_V_1.5_ext_, measured as the mean intensity multiplied for the CM area (middle) and the mean signal intensity (right). Data are represented as the mean±SEM (N=3, n=38-39). **B)** Representative double immunofluorescence images of K_V_4.3 and Lgi4 in Lgi4 expressing mice ventricular CMs (above) and K_V_4.3 and Lgi3 in Control mice ventricular CMs (below). A zoom of the Merge image is represented on the right, followed by the quantification of the colocalization and overlapping between K_V_4.3 and Lgi4 or Lgi3. **C)** Representative double immunofluorescence images of K_V_4.3 and Lgi3 in Control mice ventricular CMs. A zoom of the Merge image is represented on the right, followed by the quantification of the colocalization and overlapping between K_V_4.3 and Lgi3 is represented at the right. Scale bar: 20 µm. Values are represented as mean±SEM (N=3, n=38-39).


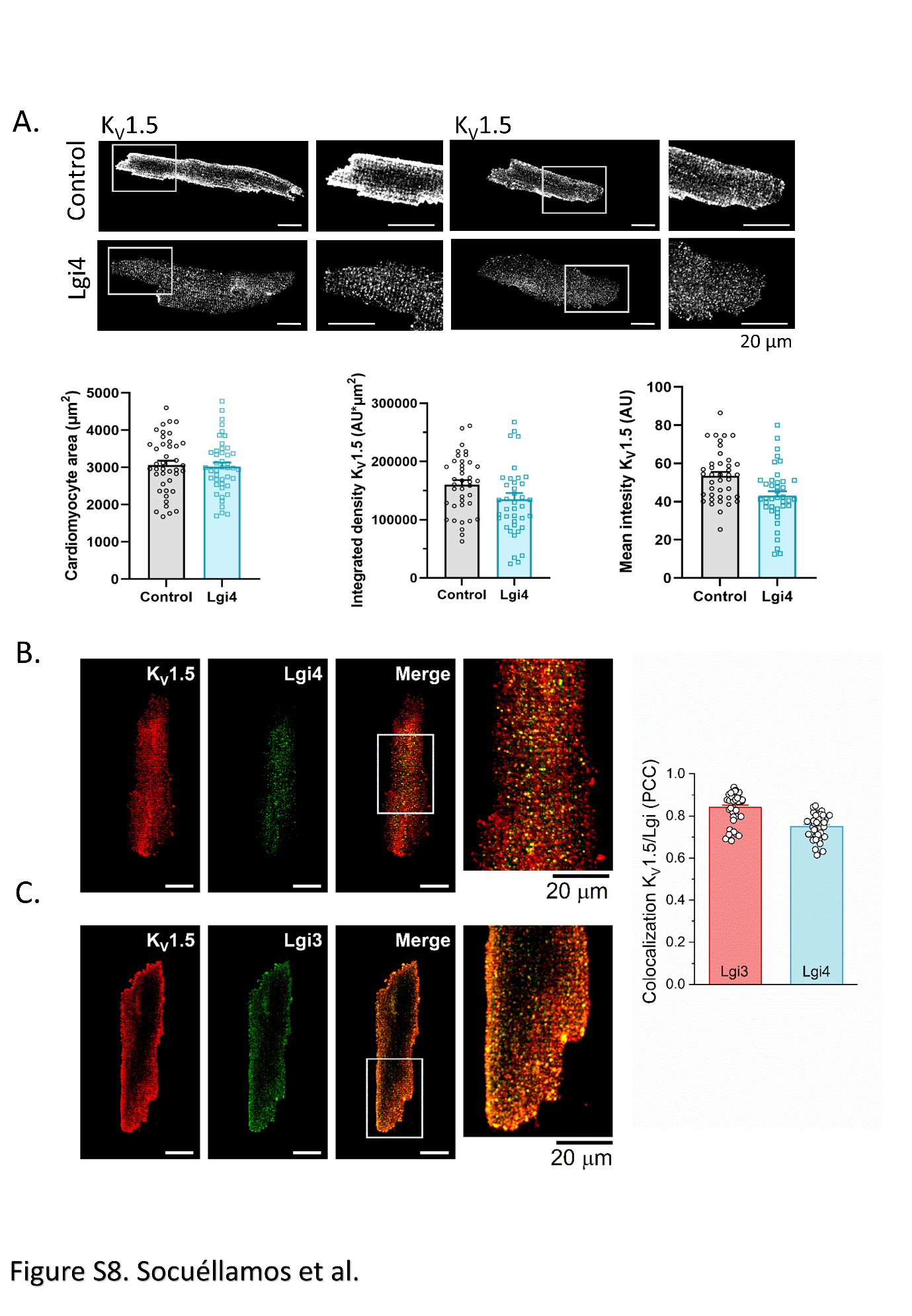
**Figure S8. Lgi4-3 colocalize with K_V_1.5 in mice ventricular CMs, but Lgi4 does not alter total K_V_1.5 expression. A)** Representative images of the immunodetection of K_V_1.5 in permeabilized mice ventricular Control (upper panels) and Lgi4 (lower panels) CMs. A zoom of the selected region is shown next to each immunostaining. Scale bar: 20 µm. The quantification of the CMs area (left), the integrated density of K_V_1.5_ext_, measured as the mean intensity multiplied for the CM area (middle) and the mean signal intensity (right) are shown below. K_V_1.5 membrane expression is not altered in either of the two measurements when comparing Lgi4 CMs to Control ones. Values are represented as mean±SEM (N=3, n=39-40). **B)** Representative double immunofluorescence images of K_V_1.5 and Lgi4 in Lgi4 expressing mice ventricular CMs (above) and K_V_1.5 and Lgi3 in Control mice ventricular CMs (below). A zoom of the Merge image is represented on the right, followed by the quantification of the colocalization and overlapping between K_V_1.5 and Lgi4 or Lgi3. Scale bar: 20 µm. Values are represented as mean±SEM (N=3, n=39-40).


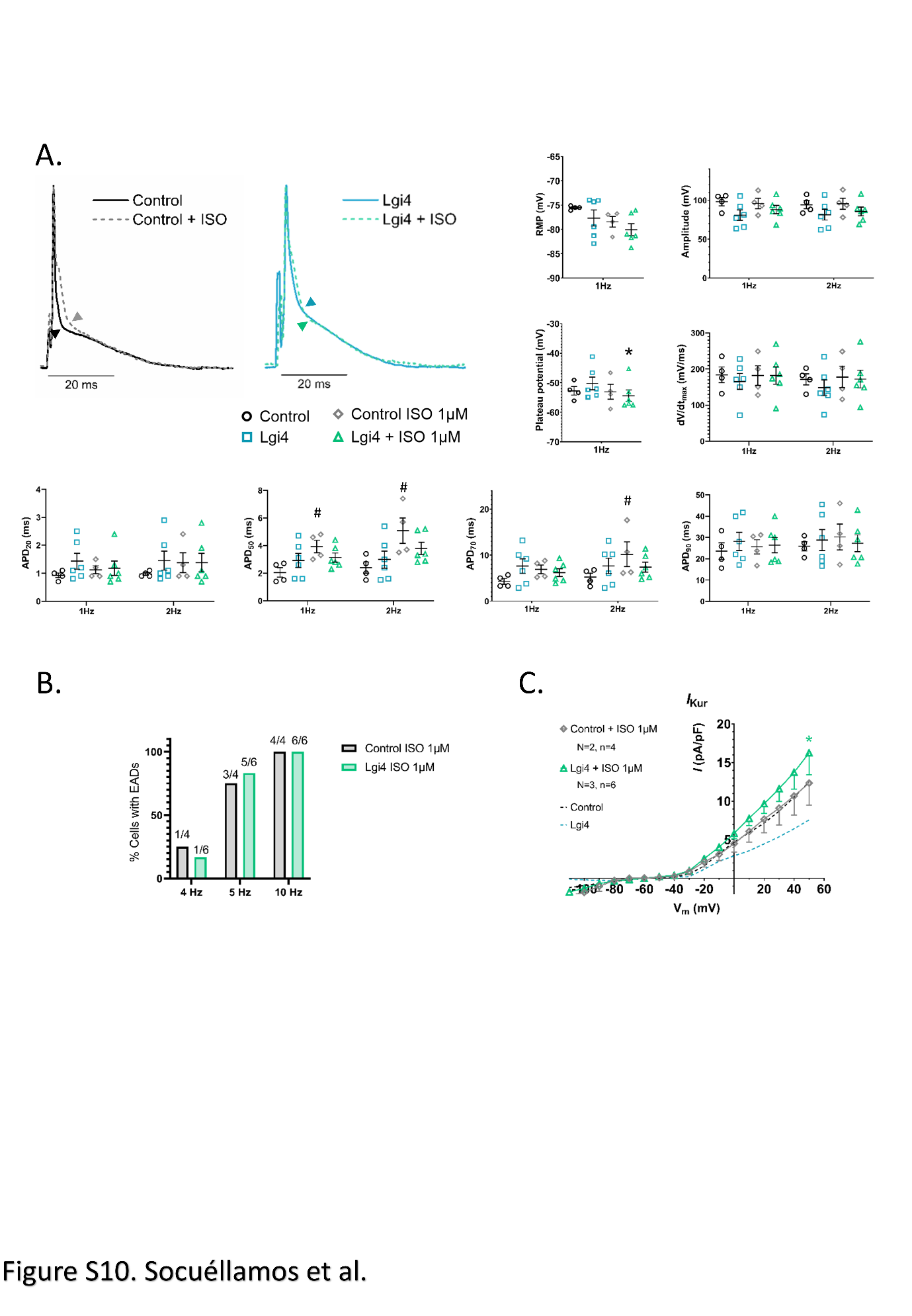
**Figure S9. Electrophysiological effects of isoprenaline on mice ventricular CMs. A)** The upper left panel shows representative normalized recordings of APs registered at 2 Hz of stimulation in Control (black) and Lgi4 (blue) mice ventricular CMs in the absence (continuous lines) and in the presence of ISO (1 µM, dash lines). The upper right panel shows the resting membrane potential (RMP), maximal amplitude of the AP (Amplitude) and upstroke velocity (dV/dt_max_). Lower graphs show the APD at the 20, 50, 70 and 90% level of repolarization. The CMs were stimulated at frequencies from 1 Hz to 10 Hz. Scale bar: 20 ms. **B)** Graph showing the percentage of cells exhibiting EADs when stimulated at 4, 5 and 10 Hz in Control and Lgi4 CMs in the presence of ISO. Data are represented as mean±SEM (N=2-3, n=4-6). **C)** Current-voltage relationship of *I*_Kur_ in the absence and in the presence of ISO (1 µM). Values are represented as mean±SEM (N=2-3, n=4-6). (Paired t-test were performed when comparing the absence and the presence of ISO in each group (#*p* < 0.05) and Two-way ANOVA with Sidaks multiple comparison when comparing all the groups between each other. Non-paired t-test were performed to compare *I*_Kur_ in the presence and the absence of ISO (C, **p* < 0.05).


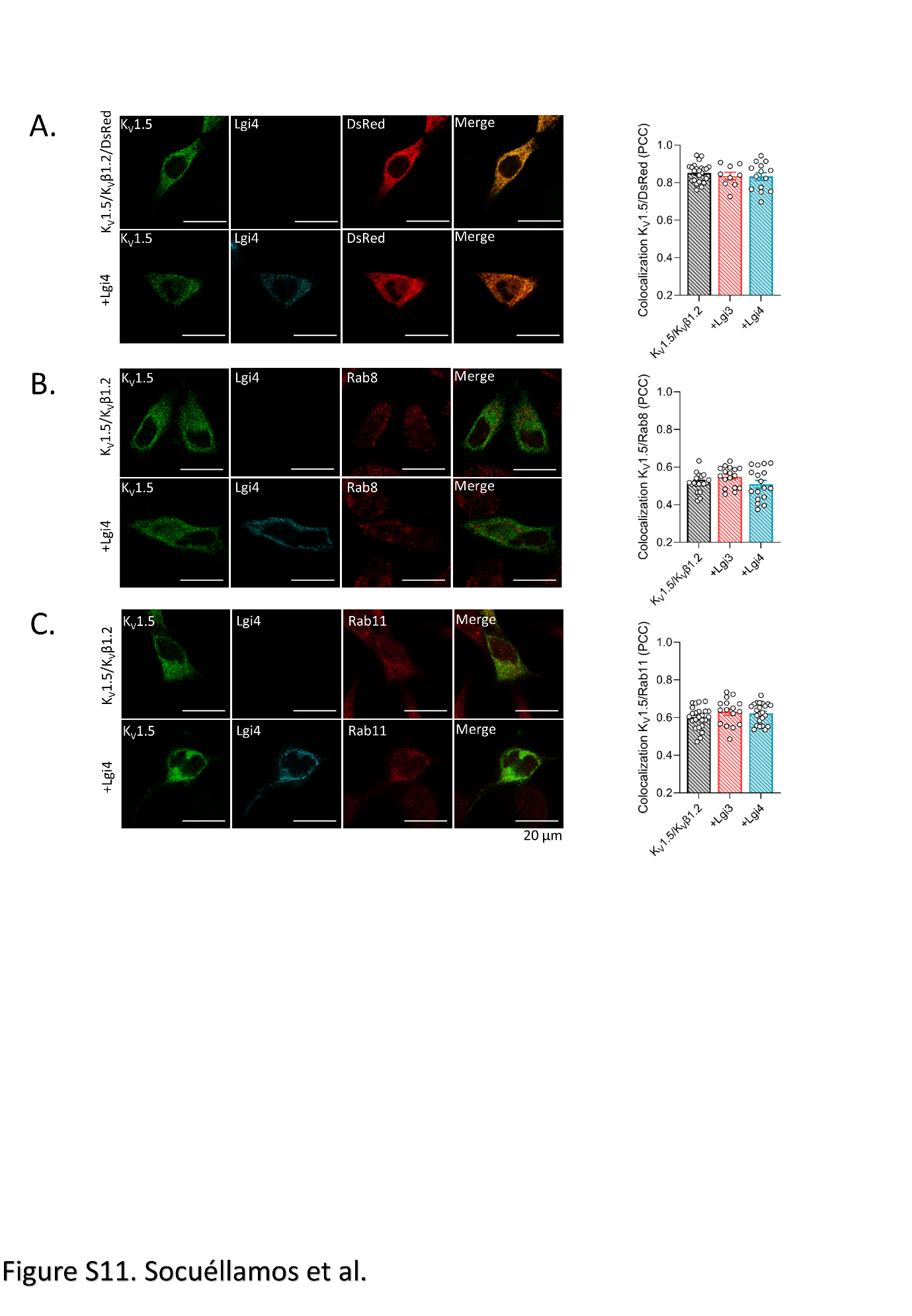
**Figure S10. Lgi3-4 do not modify ER retention, forward trafficking or recycling of K_V_1.5.** Representative confocal images of HEK293 cells cotransfected with K_V_1.5 and K_V_β1.2 in the absence (upper panels) or the presence of Lgi4 (lower panels), and stained against K_V_1.5, Lgi4 and DsRed (**A**), Rab8 (**B**) and Rab11 (**C**) with the PCC between K_V_1.5 and the corresponding marker shown in the right bar graph. Data represent mean±SEM of n=9-24. Scale bar: 20 µm.


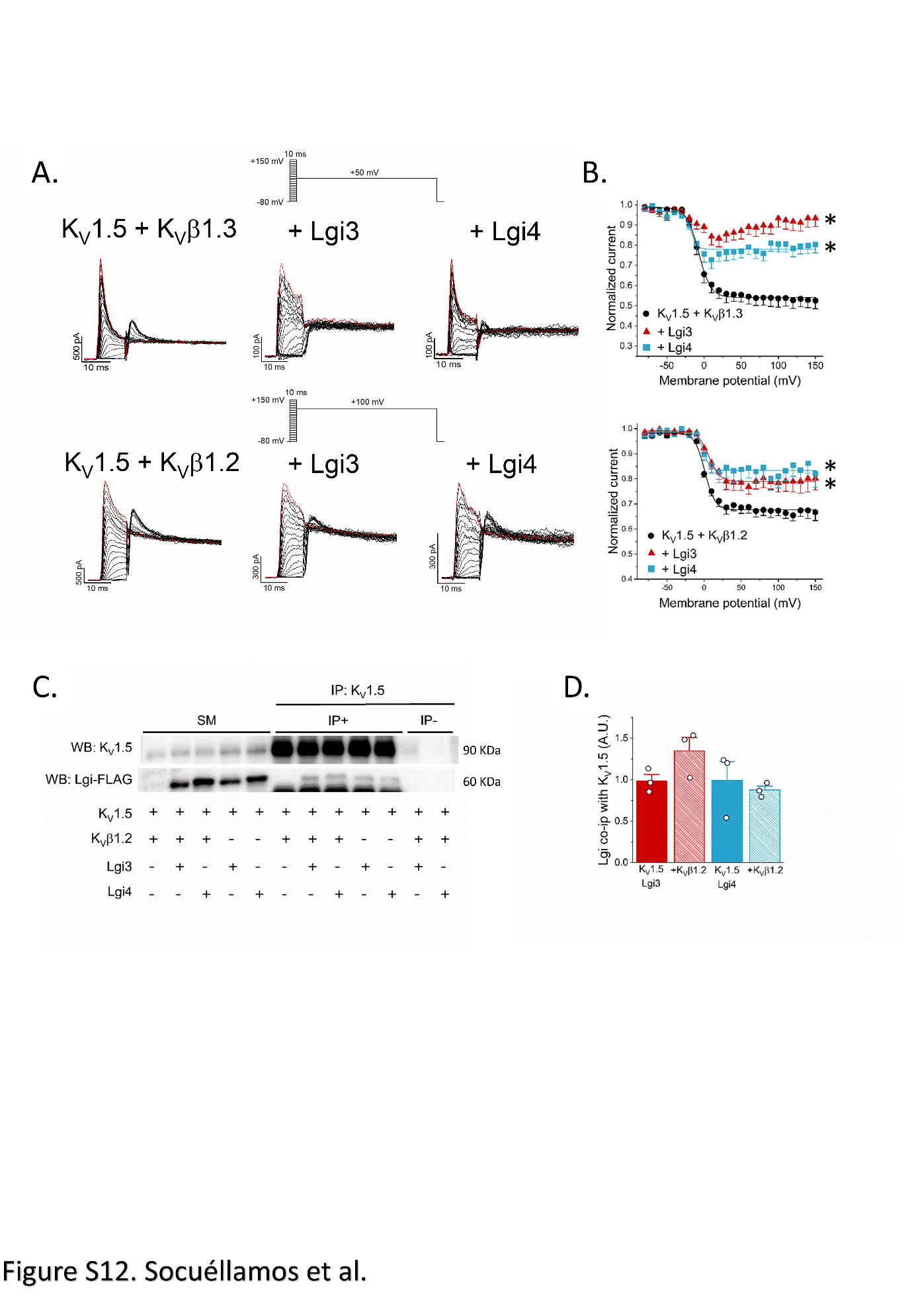
**Figure S11. Lgi3-4 do not modify the voltage-dependence of the N-type inactivation induced by K_V_β1 on K_V_1.5 channels. A)** Representative traces of the voltage-dependence of inactivation after applying a 10 ms pulse in K_V_1.5/K_V_β1.3 (upper panel, n=11-13) or K_V_1.5/K_V_β1.2 (bottom panel, n=10-11) in the absence and the presence of Lgi3 or Lgi4. Current was measured at the +50 (K_V_β1.3) or +100 mV (K_V_β1.2) pulse that follows the prepulse. Scale bar: 10 ms in the *x* axis and 500 pA in the *y* axis. **B)** Voltage-dependence of N-type inactivation after fitting a Boltzmann equation to the data. Data represent the values of the mean±SEM of the indicated n. **C**) Total lysates were immunoprecipitated (IP) against K_V_1.5 and immunoblotted (IB) versus K_V_1.5 and FLAG (Lgi3-4) (n=3). (**D**) Quantification of FLAG coimmunoprecipitation with K_V_1.5 measured as FLAG expression in the IP+ divided by its corresponding SM expression. Values represent mean±SEM of the n indicated above. SM: starting material, IP+: IP in the presence of antibody, IP-: IP in the absence of antibody. Non-paired t-test were performed comparing each condition with its corresponding control (**p* < 0.05).


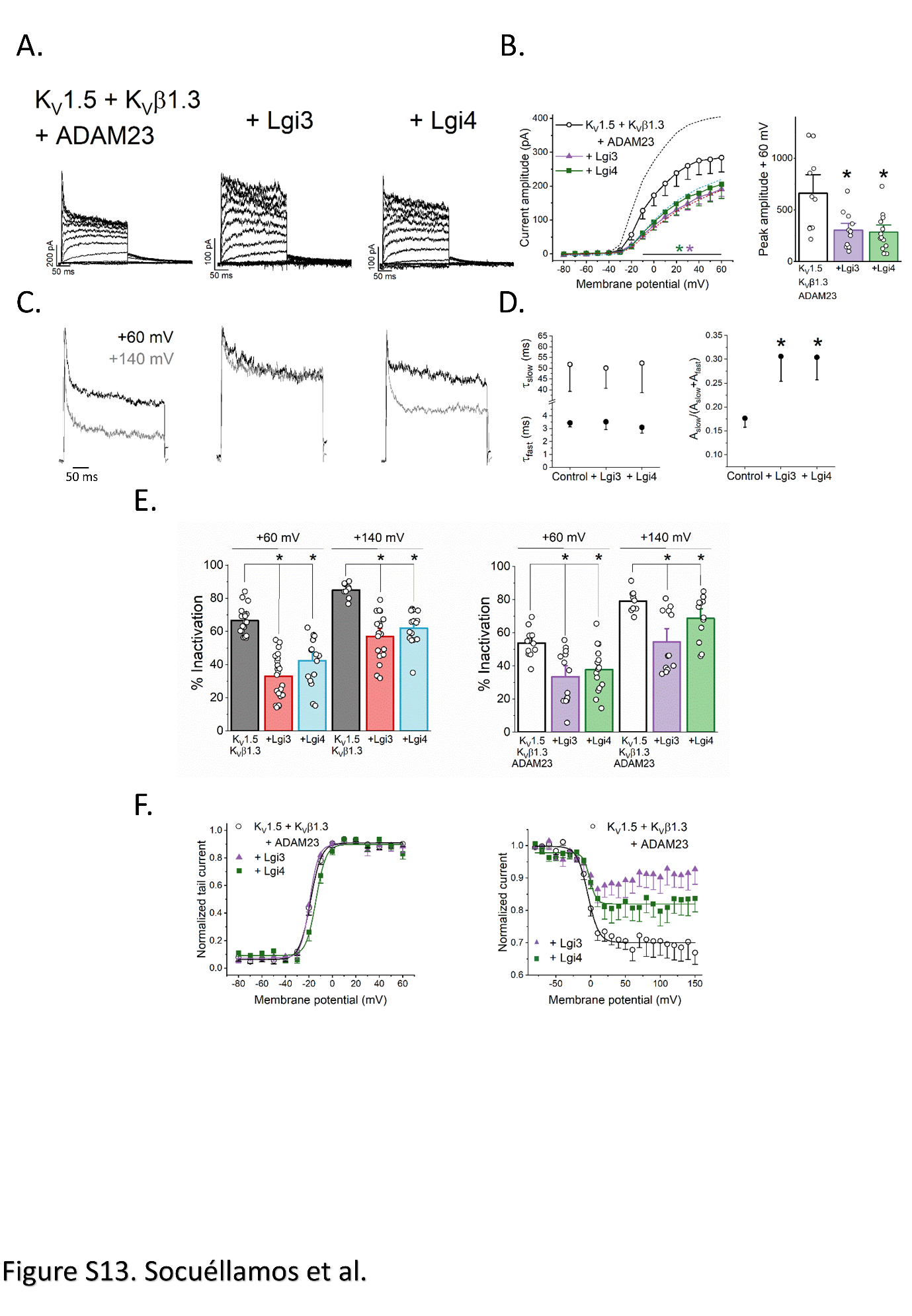
**Figure S12. Electrophysiological effects of Lgi3-4 on K_V_1.5 are not mediated through ADAM23. A)** Representative current traces elicited after application of the I-V protocol shown in the upper panel in HEK293 cells transfected with K_V_1.5/K_V_β1.3/ADAM23 with or without Lgi3 or Lgi4. The current traces are very similar to those in the absence of ADAM23 (Figure 2E). Scale bar: 50 ms in the *x* axis and the corresponding pA in each *y* axis. **B)** Current-voltage relationships measured at the end of the 250 ms depolarizing pulses (left) and current amplitude at +60 mV measured at the peak in K_V_1.5/K_V_β1.3/ADAM23 with or without Lgi3 (purple) or Lgi4 (green) (n=10-14) (right). **C)** Representative normalized current traces recorded after the application of 250 ms depolarizing pulses at +60 (black) or +140 mV (grey) in HEK293 cells transfected with K_V_1.5/K_V_β1.3/ADAM23 in the absence and the presence of Lgi3 or Lgi4 (n=9-15). Scale bar: 50 ms. **D)** Inactivation kinetics measured at +140 mV fitted to a double exponential (n=9-12). **E)** Percentage of inactivation of the currents generated in HEK293 cells cotransfected with K_V_1.5/K_V_β1.3 with (+) or without (-) ADAM23, Lgi3 and/or Lgi4 at +60 mV (left, n=11-15) and +140 mV (right, n=9-12). **F)** Voltage-dependence of activation (left, n=10-11) and N-type inactivation (right, n=7-8) after fitting a Boltzmann equation to the data. Values are represented as mean±SEM. Non-paired t-test. Data represent the values of the mean±SEM of the indicated n. **p* < 0.05 of the n indicated above.


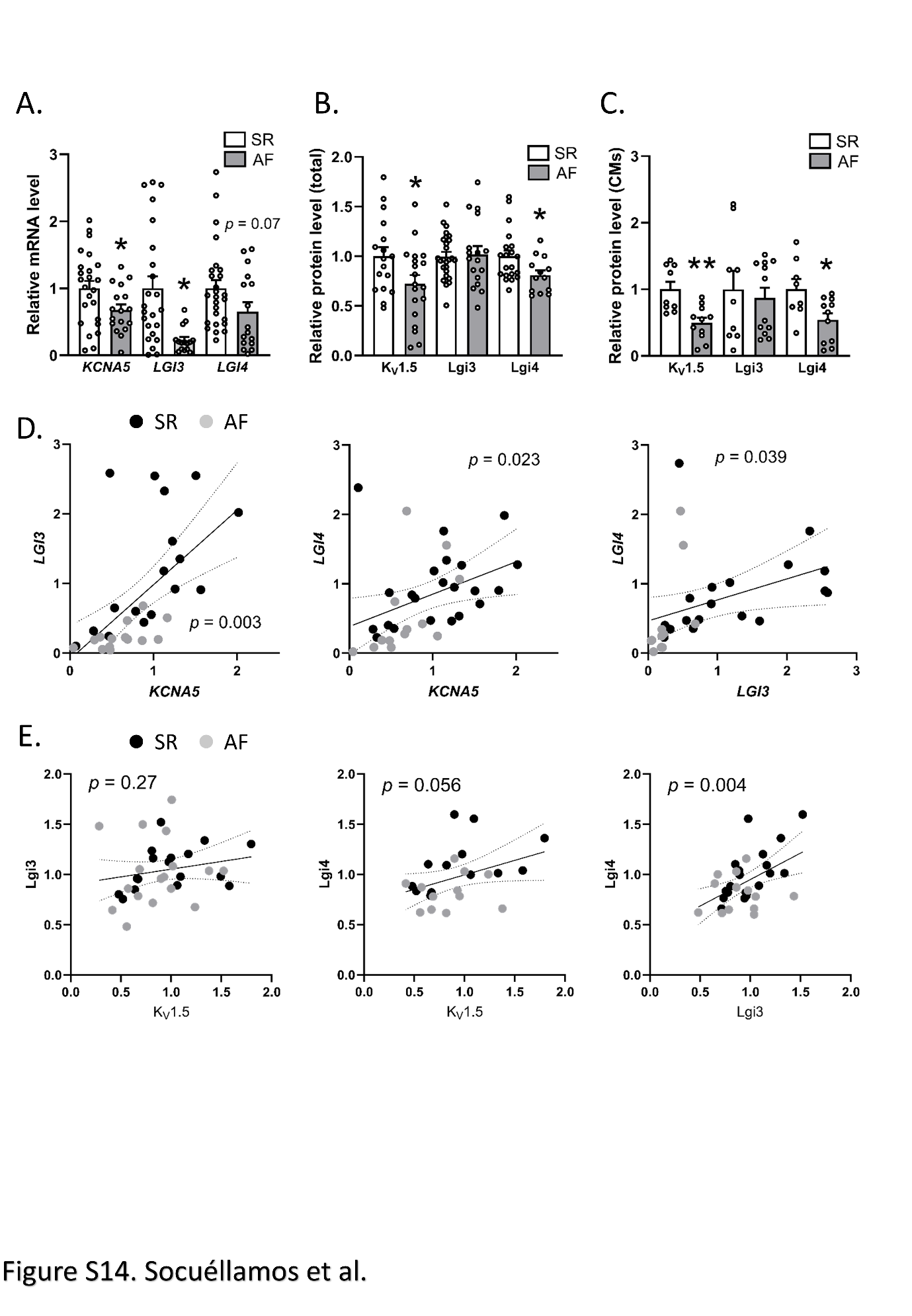
**Figure S13. Expression of K_V_1.5 channelosome in samples from patients in atrial fibrillation (AF) and in sinus rhythm (SR).** **A)** Relative mRNA expression of *KCNA5*, *LGI3* and *LGI4* in right atria tissue from patients in SR and with AF determined with qPCR. **B)** Relative protein expression of K_V_1.5, Lgi3 and Lgi4 in right atria tissue from patients in SR and with AF probed in western-blot with anti-K_V_1.5, anti-Lgi3 or anti-Lgi4 antibodies and normalized by vinculin expression. **C)** Relative protein expression of K_V_1.5, Lgi3 and Lgi4 in atrial myocytes from patients in SR and with AF measured in individual cardiomyocytes from confocal images obtained at 40X. Each point represents the mean of 50-100 CMs from two independent IF in each patient. (Non-paired t-test **p* < 0.05). **D)** Correlation between *LGI3* and *KCNA5* (left), *LGI4* and *KCNA5* (middle) and *LGI3* and *LGI4* (right) mRNA expression. The regression line is shown in black and the 95 % confidence interval in grey. **D)** Correlation between Lgi3 and K_V_1.5 (left), Lgi4 and K_V_1.5 (middle) and Lgi3 and Lgi4 (right) protein expression measured with western-blot. The regression line is shown in black and the 95 % confidence interval in grey.


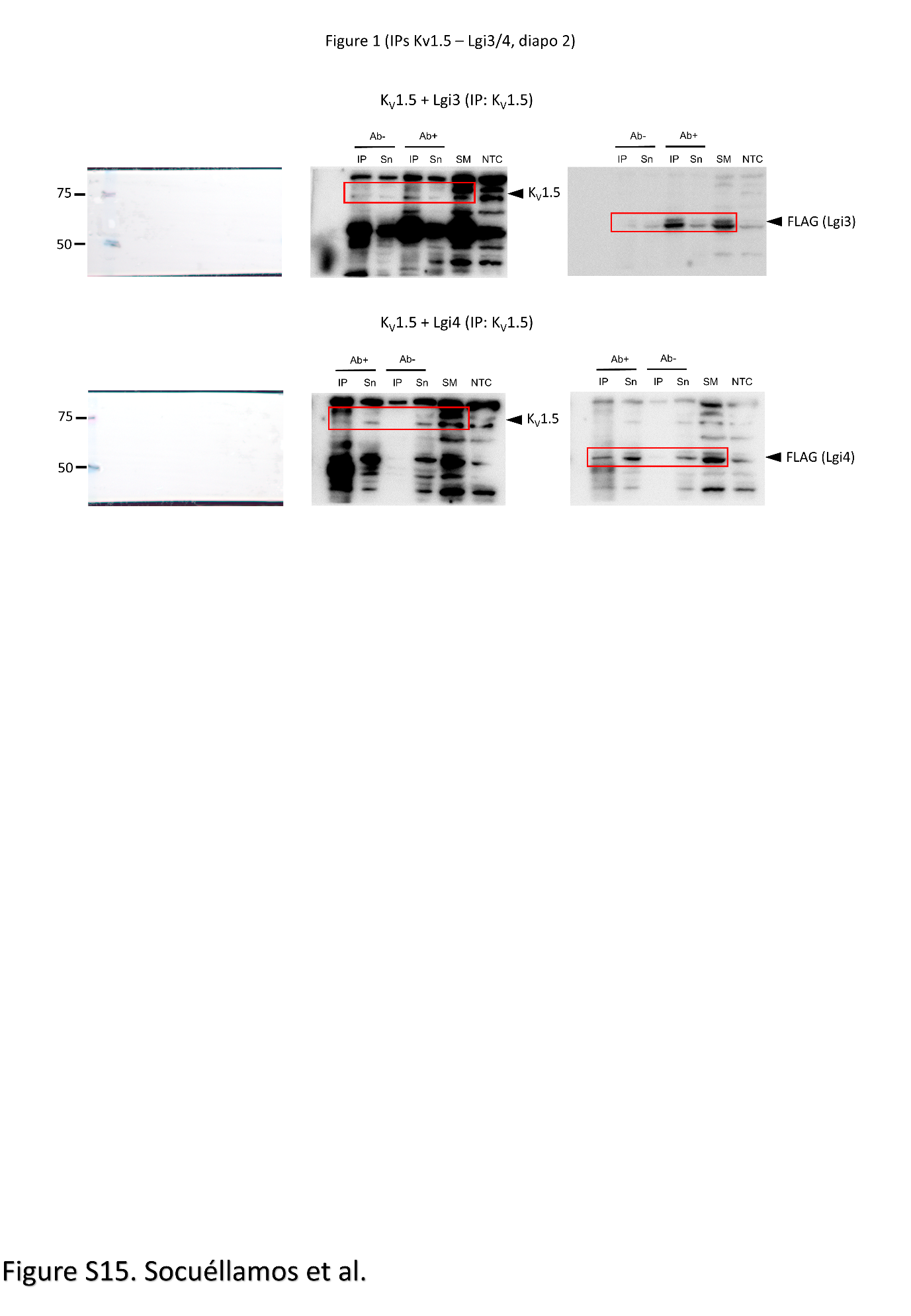


**Figure S14. Uncropped images for western blot gels presented in Figure 1. Red boxes mark the borders of the final cropped images.**


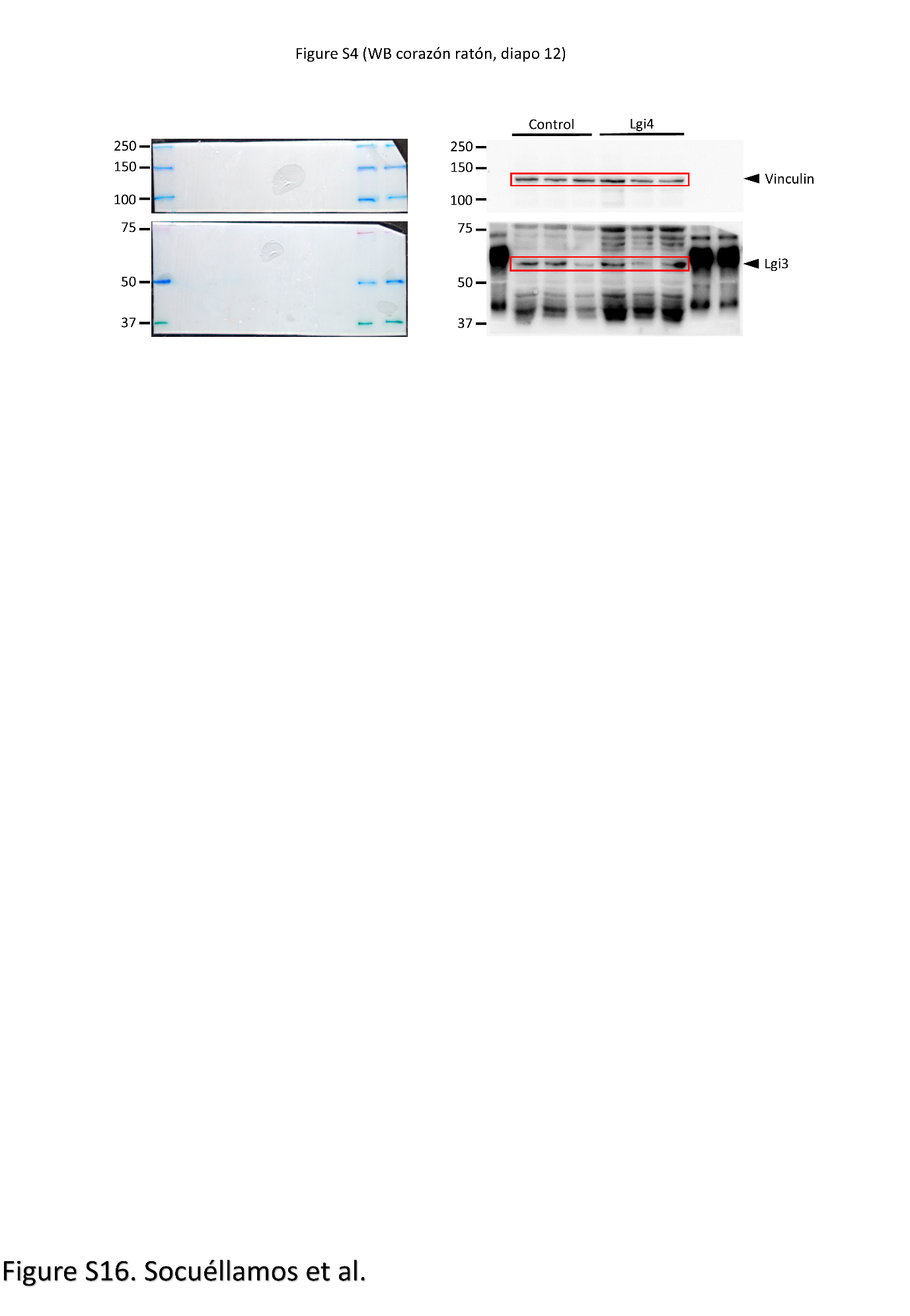


**Figure S15. Uncropped images for western blot gels presented in Figure S4. Red boxes mark the borders of the final cropped images.**

**
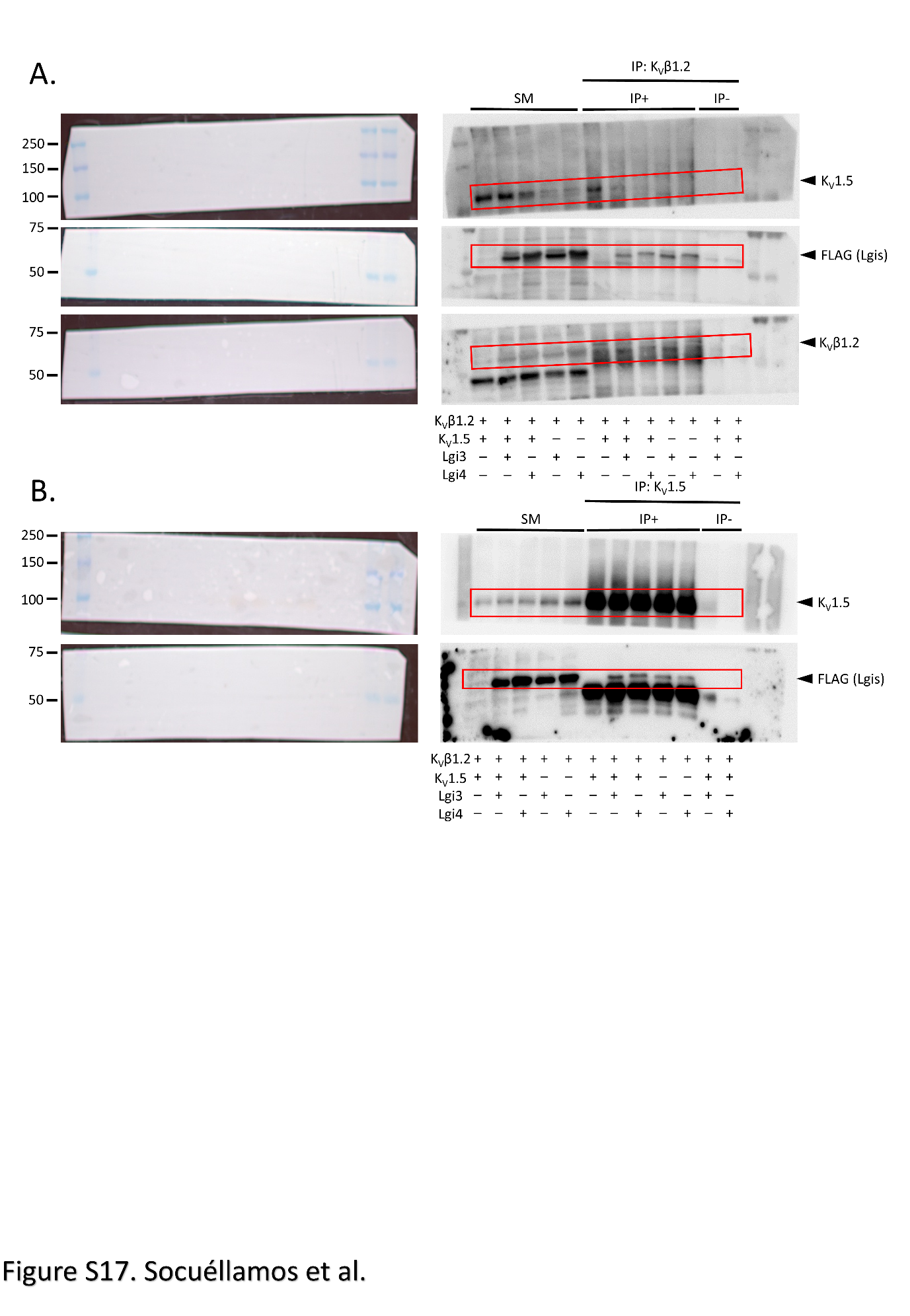
**

**Figure S16. Uncropped images for western blot gels presented in Figure 6 (A) and S12 (B). Red boxes mark the borders of the final cropped images.**


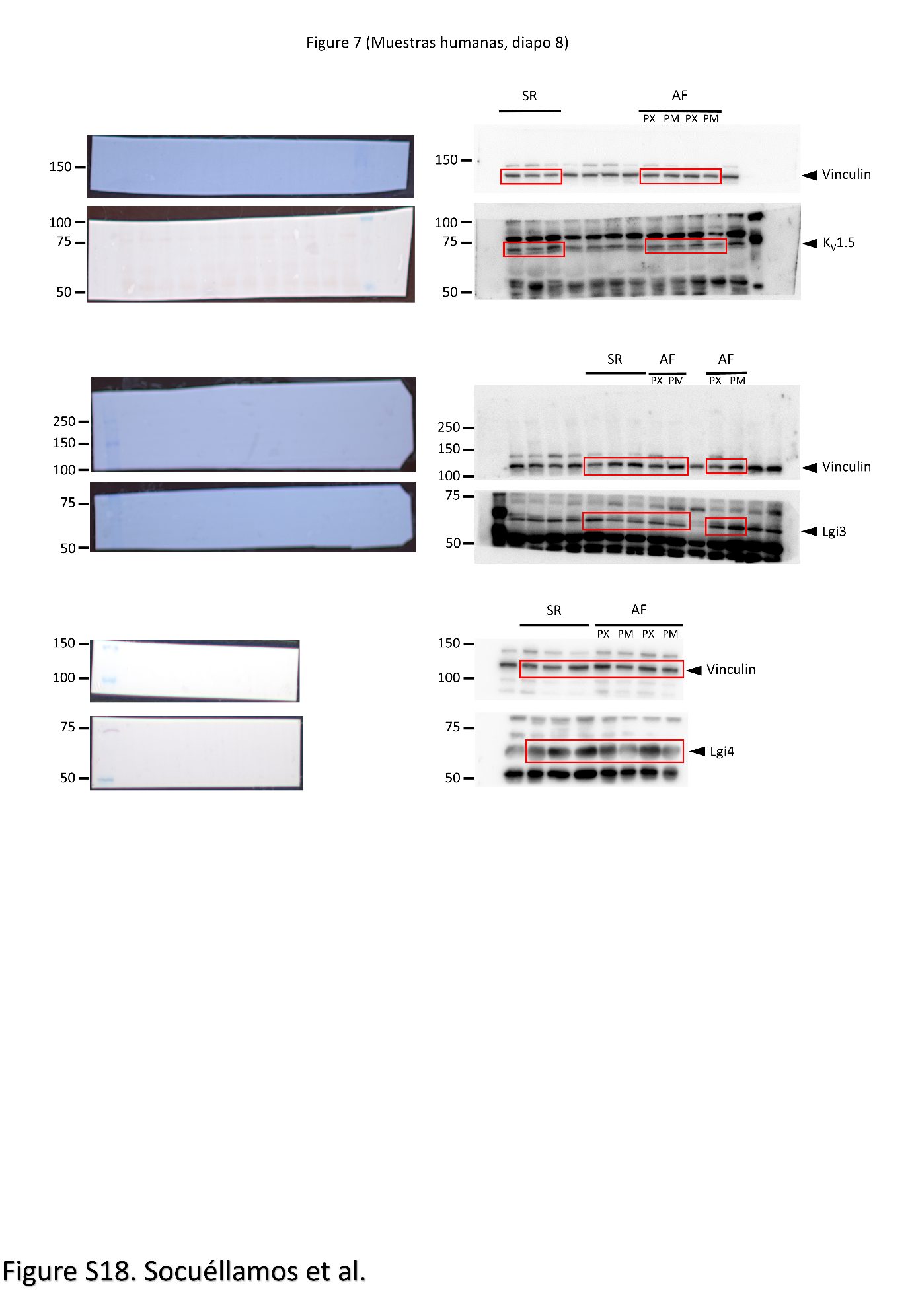


**Figure S17. Uncropped images for western blot gels presented in Figure 7. Red boxes mark the borders of the final cropped images.**

SUPPLEMENTARY TABLES

**Table S1.** Effects of Lgi3-4 on the percentage of inactivation in K_V_1.5 and K_V_1.5/K_V_β2.1 currents

|  | **% Inactivation** | **n** |
| --- | --- | --- |
| K_V_1.5 | 12.1 ± 1.0 | 11 |
| K_V_1.5 + Lgi3 | 12.3 ± 1.1 | 11 |
| K_V_1.5 + Lgi4 | 14.5 ± 1.3 | 14 |
| K_V_1.5/K_V_β2.1 | 14.9 ± 1.2 | 10 |
| K_V_1.5/K_V_β2.1 + Lgi3 | 7.8 ± 1.2 *** | 10 |
| K_V_1.5/K_V_β2.1 + Lgi4 | 8.6 ± 0.5 *** | 9 |
| K_V_1.5/K_V_β1.3 | 66.6 ± 2.0 | 18 |
| K_V_1.5/ K_V_β1.3 + Lgi3 | 33.0 ± 2.7 *** | 23 |
| K_V_1.5/ K_V_β1.3 + Lgi4 | 42.4 ± 3.7 *** | 16 |
| K_V_1.5/K_V_β1.2 | 55.7 ± 1.9 | 14 |
| K_V_1.5/ K_V_β1.2 + Lgi3 | 44.0 ± 2.9 ** | 12 |
| K_V_1.5/ K_V_β1.2 + Lgi4 | 42.6 ± 2.9 *** | 13 |
| ***p* < 0.01, ****p* < 0.001, unpaired Student’s t-test vs. its corresponding control | | |

**Table S2**. Voltage dependence of activation of K_V_1.5 in the absence and the presence of K_V_β subunits (K_V_β2.1, K_V_β1.3 or K_V_β1.2) and Lgis (Lgi3 or Lgi4).

|  | **V_1/2_ (mV)** | **s (mV)** | **n** |
| --- | --- | --- | --- |
| K_V_1.5 | -3.3 ± 0.8 | 6.2 ± 0.5 | 12 |
| K_V_1.5 + Lgi3 | -3.2 ± 0.9 | 6.6 ± 0.5 | 11 |
| K_V_1.5 + Lgi4 | -8.9 ± 0.7 ** | 6.1 ± 0.5 | 12 |
| K_V_1.5/K_V_β2.1 | -10.6 ± 1.6 | 6.9 ± 0.5 | 9 |
| K_V_1.5/K_V_β2.1 + Lgi3 | -4.1 ± 1.5 * | 7.7 ± 0.7 | 8 |
| K_V_1.5/K_V_β2.1 + Lgi4 | -4.9 ± 0.7** | 7.5 ± 0.4 | 9 |
| K_V_1.5/K_V_β1.3 | -21.8 ± 1.3 | 3.2 ± 0.2 | 15 |
| K_V_1.5/K_V_β1.3 + Lgi3 | -18.7 ± 1.4 | 5.5 ± 0.8 * | 17 |
| K_V_1.5/K_V_β1.3 + Lgi4 | -19.6 ± 0.8 | 3.5 ± 0.3 | 12 |
| K_V_1.5/K_V_β1.2 | -18.2 ± 1.4 | 4.6 ± 0.4 | 13 |
| K_V_1.5/K_V_β1.2 + Lgi3 | -14.8 ± 1.4 | 5.4 ± 0.5 | 10 |
| K_V_1.5/K_V_β1.2 + Lgi4 | -14.3 ± 1.0 | 4.3 ± 0.6 | 12 |
| **p* <0.05, ***p* <0.01, unpaired Student’s t-test vs. its corresponding control | | | |

**Table S3.** Voltage-dependence of inactivation of K_V_1.5 currents in the absence and the presence of K_V_β and/or Lgi proteins after fitting a Boltzmann equation to the data.

|  |  | **V_1/2_ (mV)** | **s (mV)** | **n** |
| --- | --- | --- | --- | --- |
| Prepulse 250 ms | K_V_1.5/K_V_β2.1 | -10.8 ± 1.6 | 6.2 ± 0.7 | 10 |
|  | K_V_1.5/K_V_β2.1 + Lgi3 | -7.5 ± 2.9 | 8.8 ± 1.4 | 5 |
|  | K_V_1.5/K_V_β2.1 + Lgi4 | -11.4 ± 1.7 | 7.0 ± 1.0 | 5 |
| Prepulse 10 ms | K_V_1.5/K_V_β1.3 | -6.8 ± 1.6 | 7.0 ± 0.6 | 10 |
|  | K_V_1.5/K_V_β1.3 + Lgi3 | -9.3 ± 2.5 | 5.5 ± 0.8 * | 4 |
|  | K_V_1.5/K_V_β1.3 + Lgi4 | -12.8 ± 0.8* | 5.2 ± 1.0 | 8 |
|  | K_V_1.5/K_V_β1.2 | -3.7 ± 1.5 | 5.4 ± 0.4 | 9 |
|  | K_V_1.5/K_V_β1.2 + Lgi3 | -3.1 ± 1.4 | 4.0 ± 1.0 | 5 |
|  | K_V_1.5/K_V_β1.2 + Lgi4 | -1.6 ± 1.6 | 6.5 ± 2.3 | 4 |
| * p<0.05, ** p<0.01, unpaired Student’s t-test vs its corresponding control | | | | |

**Table S4.** Patient characteristics.

|  | **SR** | **PX AF** | **PM AF** | **AF ablation** |
| --- | --- | --- | --- | --- |
| Patients (*n*) | 33 | 15 | 22 | 2 |
| Mean age (*y)* | 61±13 | 69±8 | 74±7 | 69±2 |
| Male (*n*) | 26 | 9 | 14 | 1 |
| Hypertension (*n*) | 15 | 8 | 13 | 1 |
| Diabetes mellitus (*n*) | 5 | 3 | 4 | 1 |
| Dyslipidemia (*n*) | 10 | 11 | 16 | 2 |
| Stroke *(n*) | 1 | 1 | 6 | 0 |
| Myocardial infarction *(n*) | 5 | 2 | 1 | 1 |
|  |  |  |  |  |
| Smoking habit |  |  |  |  |
| No (*n*) | 16 | 9 | 15 | 1 |
| Ex-smoker *(n*) | 14 | 5 | 5 | 1 |
| Current smoker (*n*) | 3 | 1 | 2 | 0 |
|  |  |  |  |  |
| Alcoholism |  |  |  |  |
| No (*n)* | 33 | 15 | 21 | 2 |
| Ex-alcoholism (n) | 0 | 0 | 1 | 0 |
|  |  |  |  |  |
| Treatment |  |  |  |  |
| Beta blockers (*n*) | 12 | 11 | 16 | 2 |
| ACE inhibitors (*n*) | 10 | 4 | 5 | 1 |
| ARBs *(n)* | 7 | 3 | 8 | 0 |
| Statins (*n*) | 14 | 9 | 17 | 2 |
| Acetylsalicylic acid (*n*) | 11 | 4 | 3 | 0 |
| Digoxin (*n*) | 0 | 0 | 5 | 0 |
| Ivabradine (*n)* | 1 | 0 | 0 | 0 |
| Calcium antagonists (*n*) | 2 | 3 | 8 | 1 |
| Loop diuretics (*n*) | 4 | 6 | 16 | 1 |
| Spironolactone (*n*) | 1 | 4 | 7 | 1 |
| P_2_Y_12_ receptor blockers (*n*) | 3 | 0 | 0 | 0 |
| Oral anticoagulants (*n*) | 0 | 12 | 22 | 2 |
| Amiodarone (*n*) | 0 | 2 | 0 | 1 |
| Flecainide (*n*) | 0 | 1 | 0 | 0 |
|  |  |  |  |  |
| Creatinine (*mg/dL)* | 1.1±0.4 | 1.8±2.7 | 1.1±0.4 | 1.2±0.1 |
| Left atrial diameter *(cm)* | 3.96±0.76 | 4.46±0.78 | 5.0±1.16 | 4.2±0.14 |
| Left atrial volume *(mL)* | 73.8±36 | 85.3±32 | 216±390 | 125.8±228 |
| Ejection fraction *(%)* | 54.1±13 | 61±15 | 59.9±8.8 | 64.9±7 |
|  |  |  |  |  |
| NYHA functional class |  |  |  |  |
| I *(n)* | 12 | 14 | 7 | 0 |
| II *(n)* | 1 | 6 | 8 | 0 |
| III *(n)* | 0 | 9 | 12 | 1 |
| IV *(n)* | 1 | 0 | 1 | 0 |
|  |  |  |  |  |
| Surgery |  |  |  |  |
| Valve surgery (*n*) | 7 | 5 | 5 | 0 |
| CABG surgery (*n*) | 8 | 1 | 1 | 1 |
| Aorta surgery (*n*) | 3 | 3 | 1 | 0 |
| Septal myectomy (*n)* | 1 | 1 | 1 | 0 |
| Atrial septal defect (*n)* | 1 | 0 | 0 | 0 |
| LVAD *(n*) | 1 | 0 | 0 | 0 |
| Combined (*n*) | 12 | 5 | 14 | 1 |

SR, sinus rhythm; PX AF, paroxysmal atrial fibrillation; PM AF, persistent/permanent atrial fibrillation; AF, atrial fibrillation; ACE, angiotensin converting enzyme; ARBs, angiotensin II type 1 receptor blockers; CABG, coronary artery bypass grafting; LVAD, left ventricular assist device. AF ablation patients were included in PX AF group.
